# Demography of a forest elephant population

**DOI:** 10.1371/journal.pone.0192777

**Published:** 2018-02-15

**Authors:** Andrea K. Turkalo, Peter H. Wrege, George Wittemyer

**Affiliations:** 1 The Wildlife Conservation Society, B.P., Bangui, Central African Republic; 2 The Elephant Listening Project, Bioacoustics Research Program, Cornell Lab of Ornithology, Ithaca, New York, United States of America; 3 Department of Fish, Wildlife and Conservation Biology, Colorado State University, Fort Collins, Colorado, United States of America; 4 Save The Elephants, Nairobi, Kenya; University of Illinois at Urbana-Champaign, UNITED STATES

## Abstract

African forest elephants face severe threats from illegal killing for ivory and bushmeat and habitat conversion. Due to their cryptic nature and inaccessible range, little information on the biology of this species has been collected despite its iconic status. Compiling individual based monitoring data collected over 20 years from the Dzanga Bai population in Central African Republic, we summarize sex and age specific survivorship and female age specific fecundity for a cohort of 1625 individually identified elephants. Annual mortality (average = 3.5%) and natality (average = 5.3%) were lower and markedly less variable relative to rates reported for savanna elephant populations. New individuals consistently entered the study system, leading to a 2.5% average annual increase in the registered population. Calf sex ratios among known birth did not differ from parity. A weak seasonal signal in births was detected suggesting increased conceptions during the wet season. Inter-calf intervals and age of primiparity were longer relative to savanna elephant populations. Within the population, females between the ages of 25–39 demonstrated the shortest inter-calf intervals and highest fecundity, and previous calf sex had no influence on the interval. Calf survivorship was high (97%) the first two years after birth and did not differ by sex. Male and female survival began to differ by the age of 13 years, and males demonstrated significantly lower survival relative to females by the age of 20. It is suspected these differences are driven by human selection for ivory. Forest elephants were found to have one of the longest generation times recorded for any species at 31 years. These data provide fundamental understanding of forest elephant demography, providing baseline data for projecting population status and trends.

## Introduction

Information on the reproductive and mortality schedules of species is fundamental for investigation of life history strategies [1, 2] as well as for diagnosing the conseravtion status of populations [3]. Such information is notoriously difficult to collect and, therefore, available only for a select number of species and populations, disporportionately represented by species of economic interest [4]. While use of data from closely related species can serve as a useful proxy for demographic modeling, such approaches can be misleading or provide weak inference regarding the drivers of actual population change [5, 6]. As such, compiling demographic information on species of interest is of high priority, particularly in respect to the development of population monitoring and conservation intervention programs for at risk species.

Collection of high resolution demographic data is particularly challenging for rare or cryptic species that utilize large ranges. While non-invasive methods for modeling spatial distribution and occupancy can provide insight into densities and population trends, such approaches often offer limited insight into the processes driving change or for identifying segments of a population experiencing the greatest threat [7]. To determine such information, individual based monitoring using remote telemetry (e.g. mark-recapture approaches using radio collars *sensu* [8]), genetic screening [9], or individual identification and monitoring projects [10] are powerful approaches. Longitudinal sampling of known cohorts of individuals, in particular, can provide rich information on population processes, that can serve to identify underlying mechanisms of change and greater insight to demography, life history and behavior (e.g., [11]). While highly valuable for these reasons, few long term, individually based studies exist given logistical difficulties of conducting such work in wild settings [10].

The forest elephants (*Loxodonta cyclotis*) of Central Africa are of high conservation concern due to their ecological importance in forest ecosystems [12], evolutionary distinctiveness [13, 14], and large scale range and catastrophic decline [15, 16]. Forest elephants fulfill fundamental ecological roles structuring vegetation in central African humid forests, the second largest contiguous forest block on earth and an important carbon sequestration zone [17]. Forest elephants are a dominant engineer of their forest landscapes, being a primary seed disperser [18, 19] and generating clearings and trails relied on by many forest animals [20]. Across their range in the Congo Basin, forest elephants are threatened primarily by poaching and increasing competition from an expanding human population [15, 16]. A recent analysis of forest elephant census data across their range shows a 62% decrease in their numbers for the period of 2002–2011 coupled with a loss of 30% of their geographical range [15].

Despite the severe threats faced by forest elephants and recent work providing insight to the bai use patterns and basic demography [6, 21], knowledge gaps of the behavior and age specific demography of this species remain. These gaps limit our understanding of its conservation status and ability to develop comprehensive conservation strategies and management programs. Our attempts to predict forest elephant demographic response to large scale decline are modeled off information generated from a handful of intensively studied populations of savannah elephants (*Loxodonta africana*) [11, 22–25]. Its cryptic nature, dense habitat, and logistical constraints for research projects greatly limit our ability to study the forest species [26]. As such, the accrual of demographic information on forest elephants is of high conservation value.

The Dzanga Forest Elephant Study, initiated in 1990 at the Dzanga Bai, Central African Republic, is the longest running individual based study on African forest elephants [21]. The study site is of high conservation value, serving as a site in the CITES MIKE programme (Convention on International Trade in Endangered Species Monitoring in the Illegal Killing of Elephants) and as part of the UNESCO World Heritage Sangha Tri-national area declared in 2012. While recognizing that caution is necessary when broadly applying measures based on a single population of elephants, the data collected in Dzanga represent unique information for this poorly characterized species. Here, we leverage this extensive dataset to augment previous work regarding bai visitation [21] and general demography [6] by summarizing age specific demographic rates, including survivorship and reproductive schedules, providing detailed information on sex and age classes most at risk in the population.

## Methods

### Study site and individual identification

This study was conducted in Dzanga Bai, a natural forest clearing (approximately 10 hectares in size) located in southwestern Central African Republic in the Dzanga-Ndoki National Park (2.963^o^ N, 16.365^o^ E). Data compiled and analyzed were collected over the 20-year period from 1990–2010 during daily observations of individually identified elephants (an average of 203 observation days/yr). Daily rainfall records used in analyses were concomitantly collected using a standard rain gauge located at the research camp, 2 km from the bai, beginning in December 1993. These data are the same used in previous analyses, with slight differences in the cohort definition, detailed below. Details of the study site and general methods used in identification and aging can be found in [21]. This study was approved by the Dzanga Sangha Project, the Central African Republic Government and the Ministry of Water and Forest Resources.

Analyses presented here used a sample of best-known, or ‘core’ females and males, that regularly visited the bai over the course of the study [21]. Criteria were designed to minimize inclusion of the most transient visitors to the bai who would amplify the risk of conflating dispersal with mortality, and also to ensure confident re-identification despite long time periods between visits. Specifically, to be included in the ‘core’ sample, adults born before the study began, or immigrating into the visiting population, had to satisfy the following criteria: (i) seen a minimum of 12 times; (ii) observed over three or more years, and (iii) with an average of at least four observations for each year they were alive. Juveniles and newborns of these core individuals were included regardless of sighting frequency. Note that, because these core individuals must be seen for a minimum of three years to enter the class, we did not include them in survival analyses until 3 years after their initial identification unless they were offspring born during the study.

Although the basic observational criteria for inclusion in analyses presented here were the same as those in previous analyses [6, 21], the datasets differ due to the specific requirements needed for new analyses (e.g., estimated birthdates for age-specific survival). Further, the dataset of core individuals used here is larger than the dataset of [6] because here we include immigrants to the visiting population while the previous paper was restricted to a fixed ‘cohort’ alive at the end of the first four years of study. The primary dataset for analyses presented below includes 1564 individuals (491 adults when first seen, 227 dependents born before 1990, and 846 offspring born during the study).

Because of the criterion that adults be observed over a minimum of three years, it took until 1996 for 85% of the population visiting Dzanga to enter the data set. Overall annual population numbers are therefore reported beginning in 1997, although survival analyses include newborns from their birthdate and others when the three year delay is satisfied. Some analyses could confidently use a less restrictive set of criteria for constructing a dataset, as indicated below. Numbers are compiled annually using ‘Dzanga years’, beginning on 1 June rather than on a calendar year basis, because of when observations began (e.g. study year 5 began on 1 June 1994). We include an assessment of the sensitivity of survivorship estimates using different criteria in the supporting information (S1 Text), but found relaxing or restricting criteria has little qualitative effects on inference. We elaborate on the rationale for these definitions in the supporting information (S1 Text) and discussion section below.

### Reproduction and sex ratio analysis

Analysis of seasonality in reproduction and the primary sex ratio was based on 883 births observed between June 1992 and May 2010, including 37 offspring born to non-core females (the first two years were omitted because observation frequency was lower in those years so aging was less accurate and some births might have been missed). This sample included all calves observed in the bai within six months of their estimated birth date and whose sex was determined. Age for these calves was observationally estimated to be within ±1 month for 50% and within ±3 months for 98% of the sample.

We examined patterns of conception across months using the sample of newborns observed within 30 days of their estimated birth date. We applied linear regression models to assess the correlation between monthly rainfall and monthly proportion of the total annual births (arcsine transform). We examined primary sex ratios using all newborns observed and sexed within 3 months of their estimated birth date whether or not they were in ‘core’ groups. We applied the binomial test to examine sex ratio at birth and binomial (logistic) regression to evaluate the relationship between monthly sex ratio and monthly rainfall (a proxy for female physical condition), known population size and whether the population was stable or growing (binary predictor).

We applied two parallel analyses to examine the relationship between inter-birth interval and mother age, calf sex, and calf survival. For these analyses calves were first observed within 3 months of birth and birthdates assigned based on body size, coordination, and physical development [27]. In the sample of 618 such intervals, 338 were known intervals (a second calf observed) and 280 were censored intervals because the gap between successive observations was long enough that a birth could have been missed. In these cases, the interval ended with the last observation of the female before the gap. For the sample of known intervals, we used mixed model regression (GLMM) with female identity as a random effect. To take advantage of the information provided by censored intervals, we used Cox regression (proportional hazards) with female identity as a random effect. In both analyses, we used female age as a continuous variable, with a quadratic term to test for potential slower reproductive rates in older females. Finally, we also entered female age as a class variable in a Cox regression model, where females were grouped into three age-classes: younger than 25 yrs., 25–39 yrs., 40–59 yrs. The younger class cutoff point was informed by a median age at first calf of 23 years old [6] where the youngest age-class was chosen to include 12 females that gave birth to their first offspring at 24 years of age. The older class cutoff point was somewhat arbitrary, but provided a reasonable sample size and included all of the oldest females in the population.

### Mortality data collection and analysis

Estimated mortality dates were based on (1) observation of carcasses of known elephants (rare—with 16 cases in total), (2) very young, dependent calves missing when observing their known mother (rare), and (3) individuals not seen for four times their inter-visit interval [21]. Individuals in the latter category were assigned a death date of twice their inter-visit interval [21]. These criteria were established to minimize the propensity for individuals to be erroneously assigned as dead (i.e. subsequently observed alive), which occurred rarely under the stated criteria. To discern dispersal from mortality, social context was used to ascribe cause of disappearance (dispersal versus death) of dispersal aged individuals (Males were found to disperse as young as seven years and females around 13 years [21]. Specifically, when a juvenile or sub-adult was observed in the clearing without the natal group on two or more consecutive sightings it was considered to be initiating a break with the natal family group. When such an individual was seen for at least nine months after behaviorally initiating separation, we considered it individually identifiable and subsequent disappearance was coded as mortality. If the individual disappeared before the nine-month period, we applied a set of criteria, including age and social factors, to determine whether a dispersal event (censored in survival analyses), or likely mortality. For juveniles between two and 13 years of age, if the entire social group disappeared together, or the mother was seen less than twice in the year after the last sighting of the juvenile, the juvenile was assigned as having dispersed (censored). If the mother was seen more than three times in the year following disappearance or was seen in multiple years, we attributed disappearance to mortality for males younger than 7 years and females younger than 13 years. Females were much more likely to stay with the family group (or associate with them) and so identity was retained by association.

Cox regression models were employed for survival analysis, allowing for variable age at entry and time-dependent covariates. We analyzed the monthly survival of 538 newborns (289 females, 249 males), in which mother age class (<25 years old, 25–32 years old, 33–39 years old, and 40 years old or more) was used as a proxy for experience. Annual survival of the entire core population of elephants (n = 1564) was analyzed with respect to age and whether certain age-classes experienced higher mortality. For the latter analysis we grouped individuals into three large age-classes based on their age when entering the risk pool: 0–9 years, 10–23 years, and over 23 years, where the 10 year cut-off related to the age when dispersal becomes increasingly common, and 23 years was the average age of primiparity [6].

A similar approach was used to model the dispersal age of juveniles (defined above). The first date of independence was used to define the dispersal year. The dispersal sample of 843 individuals excluded juveniles/subadults last seen when less than six years old, or first identified at more than 20 years old. New family groups regularly immigrated into the Dzanga population so we limited analyses to individuals that were less than 10 years old when first seen. Individuals last seen while still with the natal group were censored.

All statistical analyses were run using SAS®9.4 (Cary, NC, USA) and tests were considered significant at an experiment-wise alpha level of 0.05. Data are included in the supporting information (S1, S2, and S3 Tables).

## Results

### Annual natality and mortality

**Fig 1:**
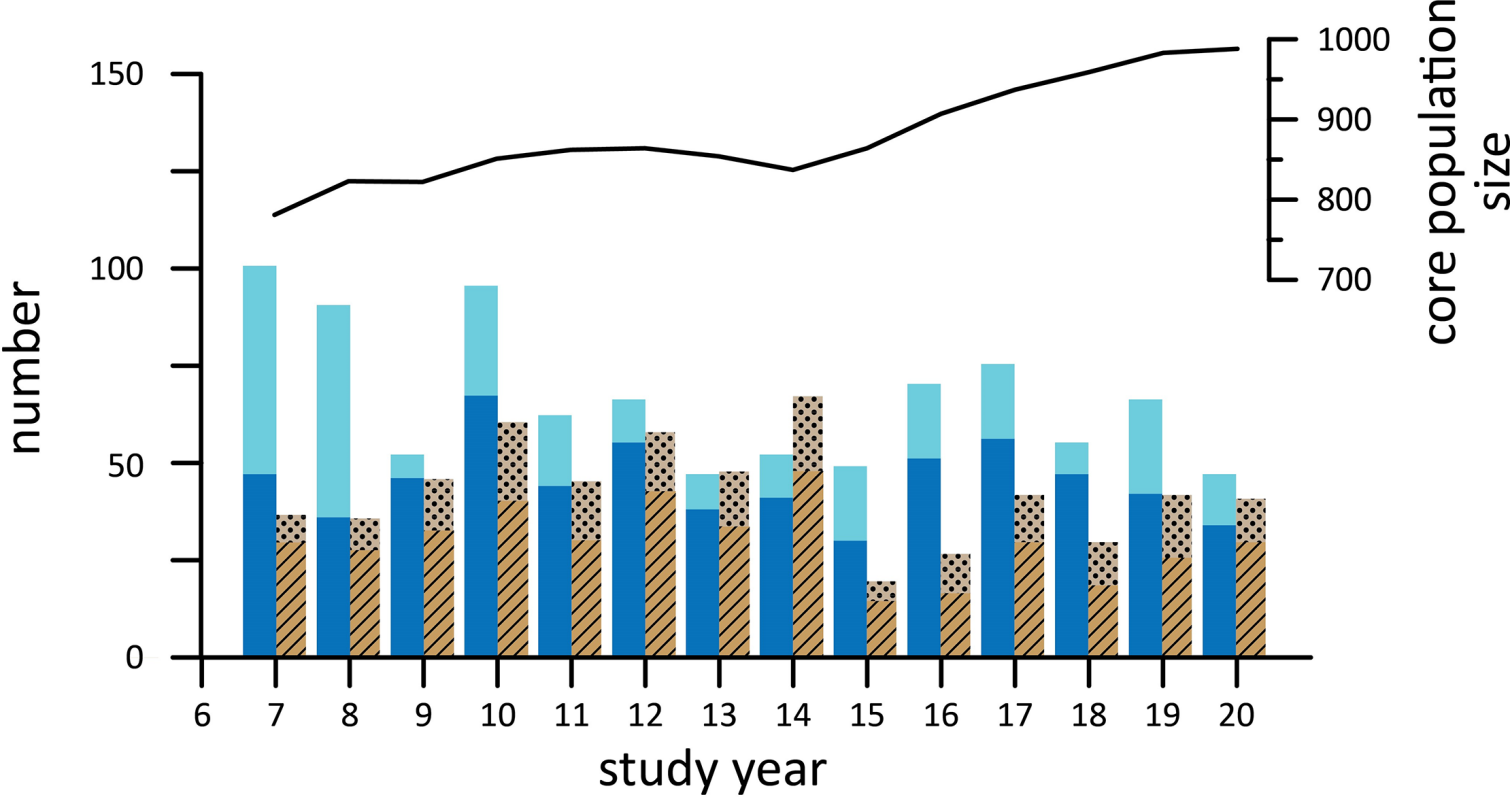
Annual Dzanga demography and population trend from 1997 to 2010. Net inflows (births, dark blue; immigration, light blue) were higher than net losses (death, dark brown; dispersal, light brown) in most years.

The sample of ‘core’ elephants visiting the Dzanga Bai increased from 781 in 1997 to 988 individuals by 2010 (Fig 1), driven by intrinsic growth and immigration. Natality among this cohort exceeded mortality in all but one year (study year 14), averaging 5.3% (SD 1.3) and 3.5% (SD 1.2) respectively. Assuming that disappearances represent mortality and not dispersal, average mortality per year could be as high as 4.9% (SD 1.6). Immigration of new adult males and family groups occurred throughout the study period, adding an average of 2.5% (SD 2.1%) to the population each year.

Based on a sample of 864 calves first seen within three months of birth, the sex ratio at birth was not significantly different from 1:1 (exact test of binomial proportion p = 0.39; 445 females: 419 males). There was no evidence for any effect of cumulative rainfall (month of conception, month prior to conception, sum over two months) on the sex ratio (all likelihood ratio χ^2^ <0.8 for probability of a female birth and all p>0.36).

**Fig 2:**
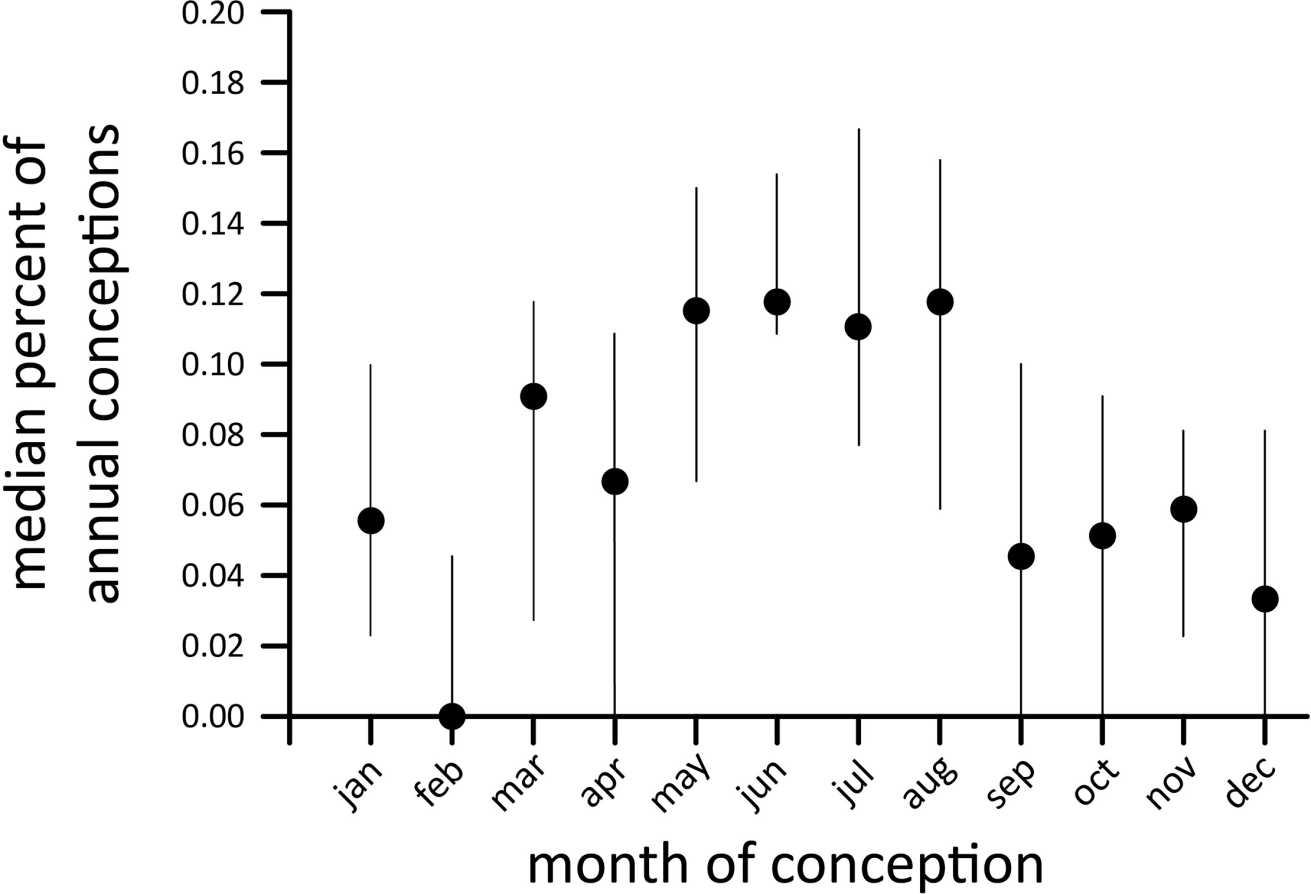
Seasonality of conception collected from 421 calves conceived between 1991 and 2007 (back calculated from birth assuming a gestation of 22 months). Lines indicate the 95% confidence intervals on the median value.

If conception was evenly distributed through the year, the expectation would be that about eight percent of the annual total would occur in each month. However, nearly 50% of females at Dzanga conceived between May and August, the midst of the March-November rainy season (Fig 2). Although month was a significant predictor of conception timing (GLM analysis of arcsine percent of annual conceptions; F_s(11,192)_ = 4.07; p<0.0001; R^2^ = 0.19), there was considerable variation among years and only February had significantly fewer conceptions than the four peak months when adjusted for multiple comparisons. Within the eight-month rainy season, we found no correlation between conceptions and cumulative monthly rainfall for the month of conception, the previous month, or cumulative rainfall over the two months before conception (GLM analysis of arcsine percent of annual conceptions; F_s(1,124)_ = 2.41, 0.08, 1.19 respectively; all p>0.1).

The length of time between sequential births (inter-birth interval) was influenced by female age, with intervals decreasing in length as females aged, but then increasing again for the oldest females (Table 1). Survival of the calf to 48 months of age was also significant in our GLMM analysis, while sex of the first calf was not. Although calf mortality had a strong effect, shortening intervals by about 31 months, only six calves died before they were two years old.

**Table 1:** Model results for evaluating influences on inter-birth interval in forest elephants.

Predictor	Parameter estimate	Test statistic	p-value
a.			
intercept	91.9	8.45	<0.01
female age (continuous)	-1.68	-2.28	0.02
female age squared (continuous)	0.02	1.85	0.06
calf sex = female	-2.06	-1.02	0.31
calf dies <24 months	-31.05	-4.06	<0.01
b.			
female age (continuous)	1.8	12.96	<0.01
female age squared (continuous)	-0.31	14.53	<0.01
calf sex = female	0.05	0.12	0.73
calf dies <24 months	0.59	0.96	0.33
c.			
female < 25 yrs old	-0.45	7.7	<0.01
female > 39 yrs old	-0.76	16.48	<0.01
female 25–39 yrs old	0	-	-
calf sex = female	0.07	0.31	0.56
calf dies <24 months	0.54	0.84	0.36

All models included female identity as a random effect and covariates for mother’s age, sex and survival of the calf initiating the interval. Models estimate the birth interval in months. (a) GLMM for observed intervals (n = 338) with female age as a quadratic term. (b) Cox proportional hazards model including censored observations (n = 618), with female age as a quadratic term. (c) Cox proportional hazards model with female age as a class variable. Both Cox models estimate the hazard of a birth event.

The influence of these covariates on inter-birth intervals was also examined using Cox regression analysis (proportional hazards) including 280 additional intervals that were censored because the female disappeared or the study ended without observation of a subsequent birth. After controlling for repeated observations on the same female, neither the sex of the first calf nor its survival to 48 months was significant (Wald χ^2^ = 0.12 and 0.96 respectively, both p>0.19). The mother’s age was significant both as a continuous variable (with quadratic term) and as a class variable (Table 1B and 1C). Females younger than 25 years and those that were 40 years or older were close to half as likely to give birth compared to females between 25 and 39 years old. the median interval for older females was 75 months, 14 months longer than for females in their prime. The youngest females, most of whom had only given birth to one calf, waited a full year longer than females in their prime, most of whom had given birth to two or more calves.

### Survival

**Fig 3:**
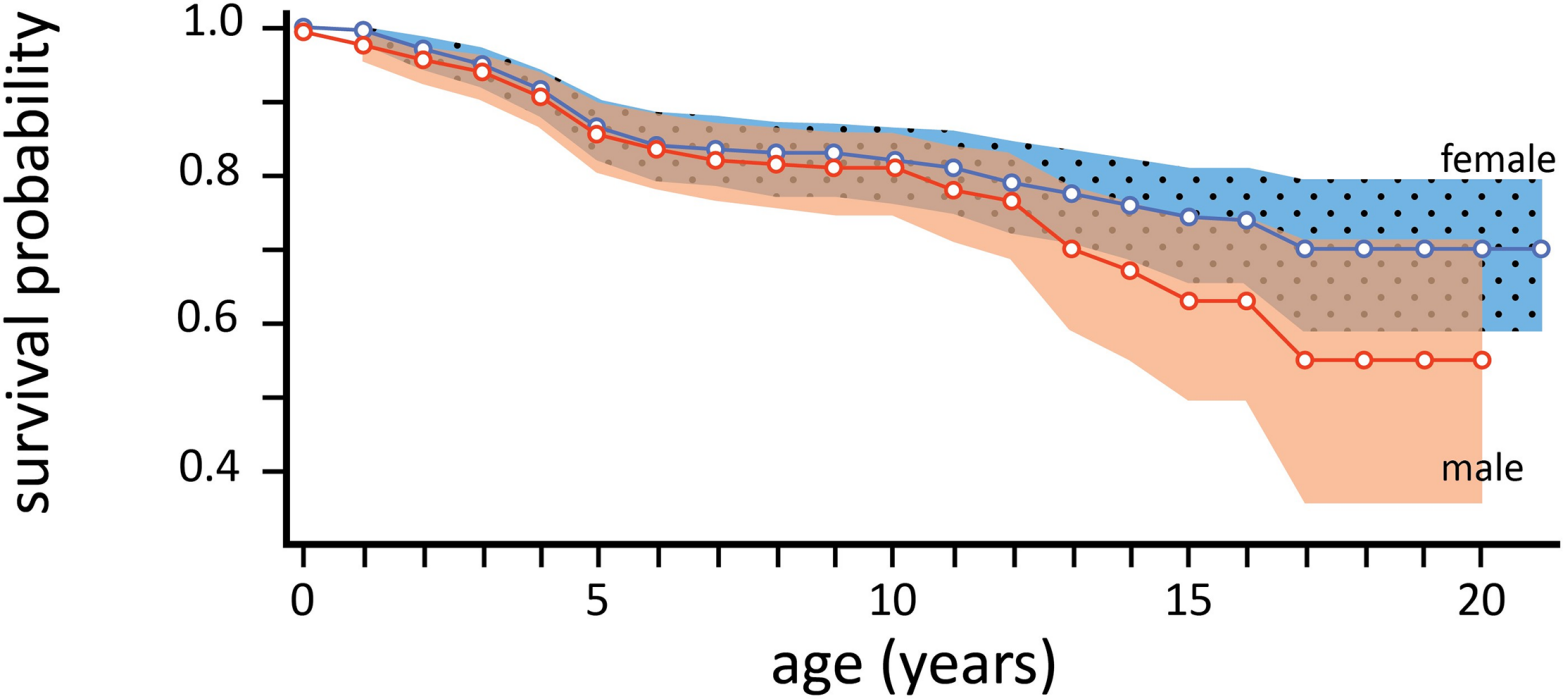
Female (blue) and male (red) survivorship through the first 20 years of age, estimated using cohort analyses of offspring with age accuracy of 3 months or better. Shaded areas are the point-wise 95% confidence interval on the survival probability.

In a sample of 538 newborns first seen before three months of age, survival was over 97% for each sex in the first year, and remained above 85% to age five. Only five females and seven males died within their first year of life. Survival was 80% through the age of ten years for both males and females (Fig 3). Males begin having a higher risk of mortality, as defined in this study, at about age 13, and the 95% confidence intervals around survival probabilities were distinct after the age of 20. Beyond ten years, males appear to suffer higher mortality than females (Fig 3).

**Fig 4:**
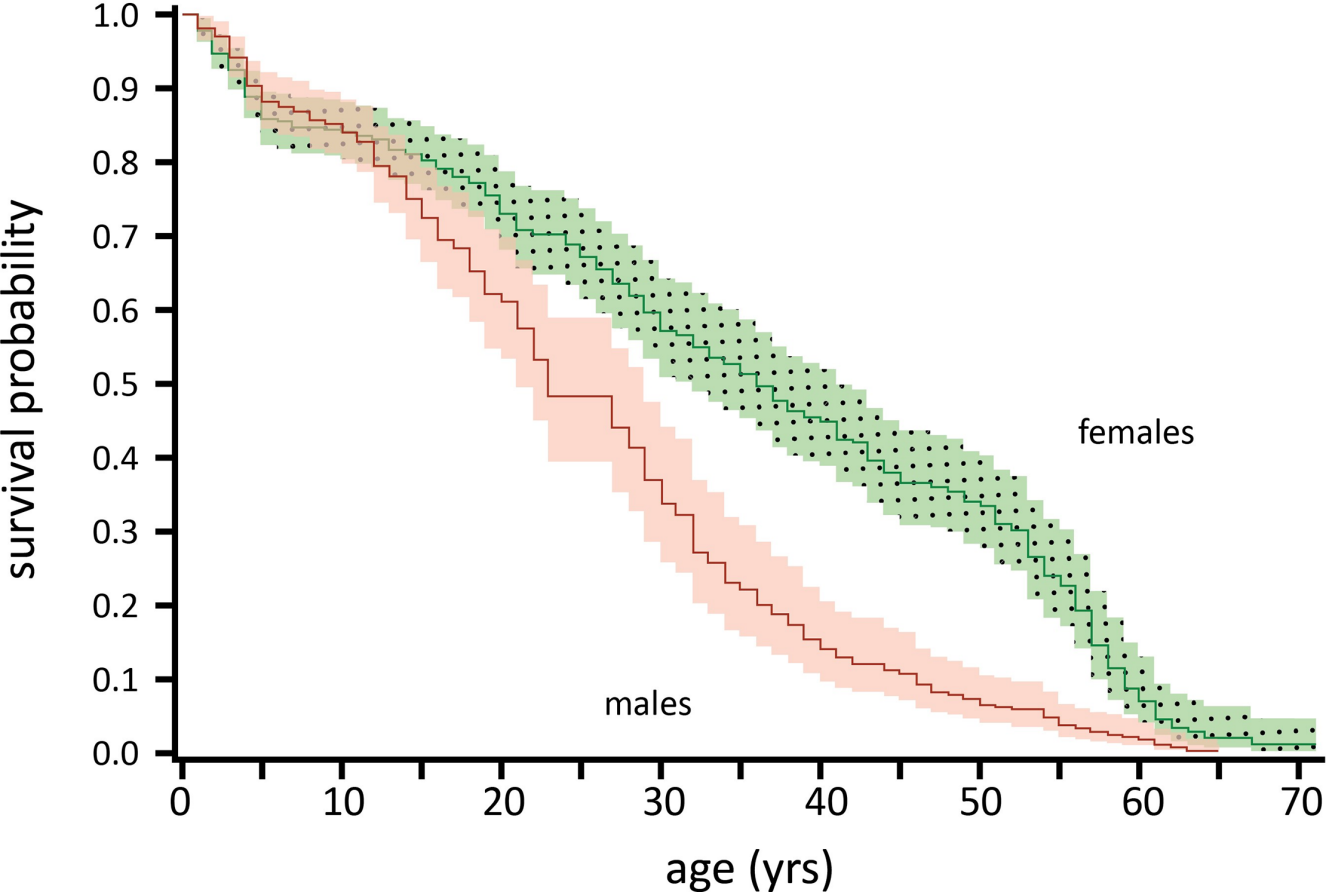
Female (green) and male (red) survivorship in the Dzanga population (shaded areas show point-level 95% CI). Females were generally twice as likely to survive to a given age as were males.

In our analysis of all individuals in the core dataset, sex was highly predictive of lifetime survival (Wald χ^2^ = 27.8, p<0.01), with males about twice as likely to die at a given age compared to females (Fig 4). However, analysis of individuals observed since birth demonstrated that survival of calves to ten years of age was not related to sex, whether the calf was the firstborn for that mother (both Wald χ^2^ < 0.001, p>0.9), or mother’s age class (Wald χ^2^ = 4.07, 3df, p>0.2). However, an individual’s age when its younger sibling was born was significantly negatively correlated with likelihood of death (Cox Regression, with age at birth of a sibling entered as an age-dependent covariate, Wald χ^2^ = 8.18, p< 0.005). Individuals with a sibling were 22% less likely to die relative to calves without a sibling.

Cox regression evaluating the age at which calves become independent of their natal family group indicated there was no significant difference in natal independence age between males and females (Wald χ^2^ = 0.62, p>0.4, n = 412 females, 346 males). Median age at independence was estimated to be 15 years.

### Fecundity and reproductive value

**Fig 5:**
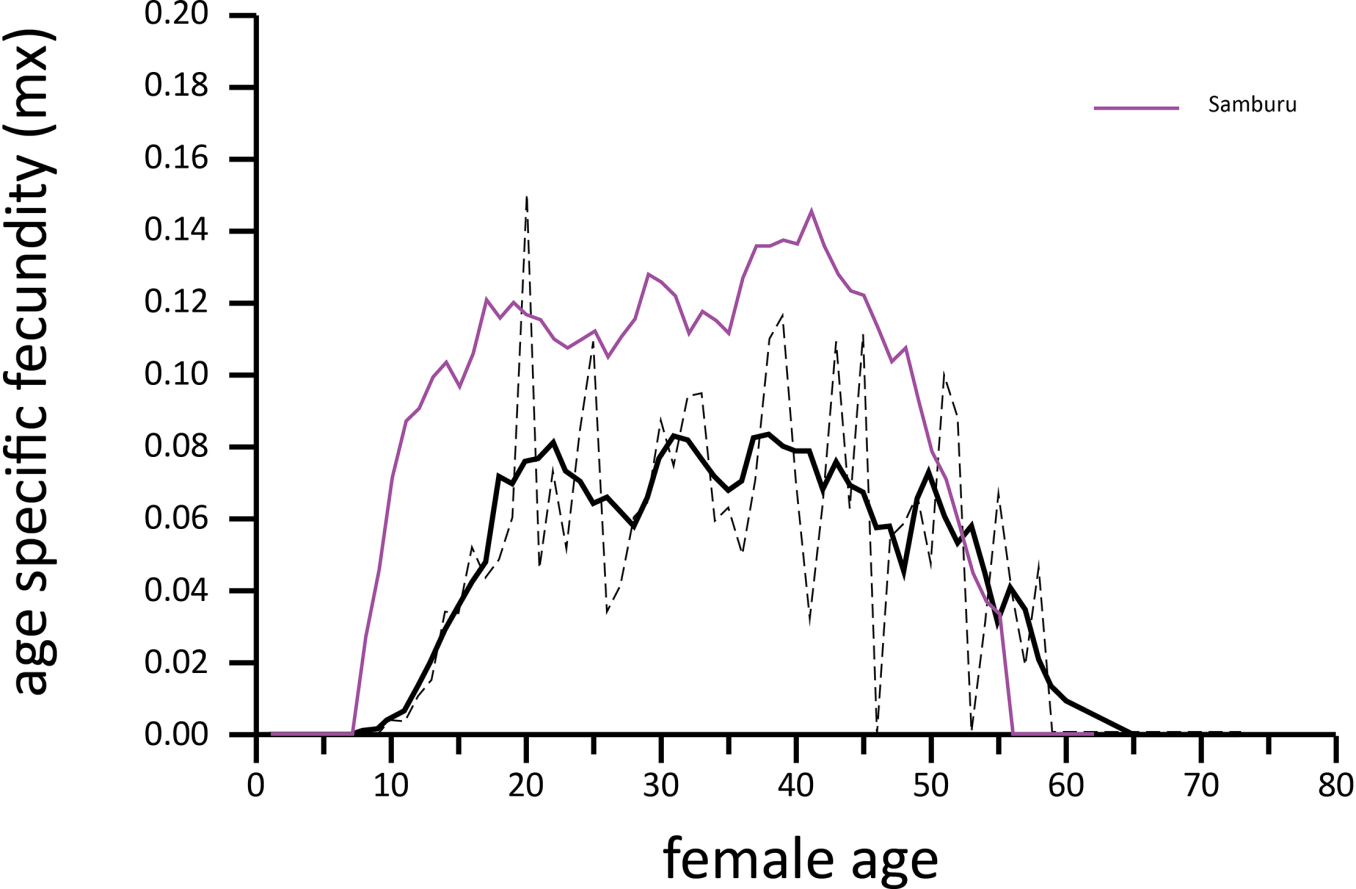
Age-specific fecundity in two populations. Dzanga (black curves): 5-yr smoothed curve (solid) and actual values (dashed). Samburu Kenya (purple curve): 5-yr smoothed curve [11].

**Fig 6:**
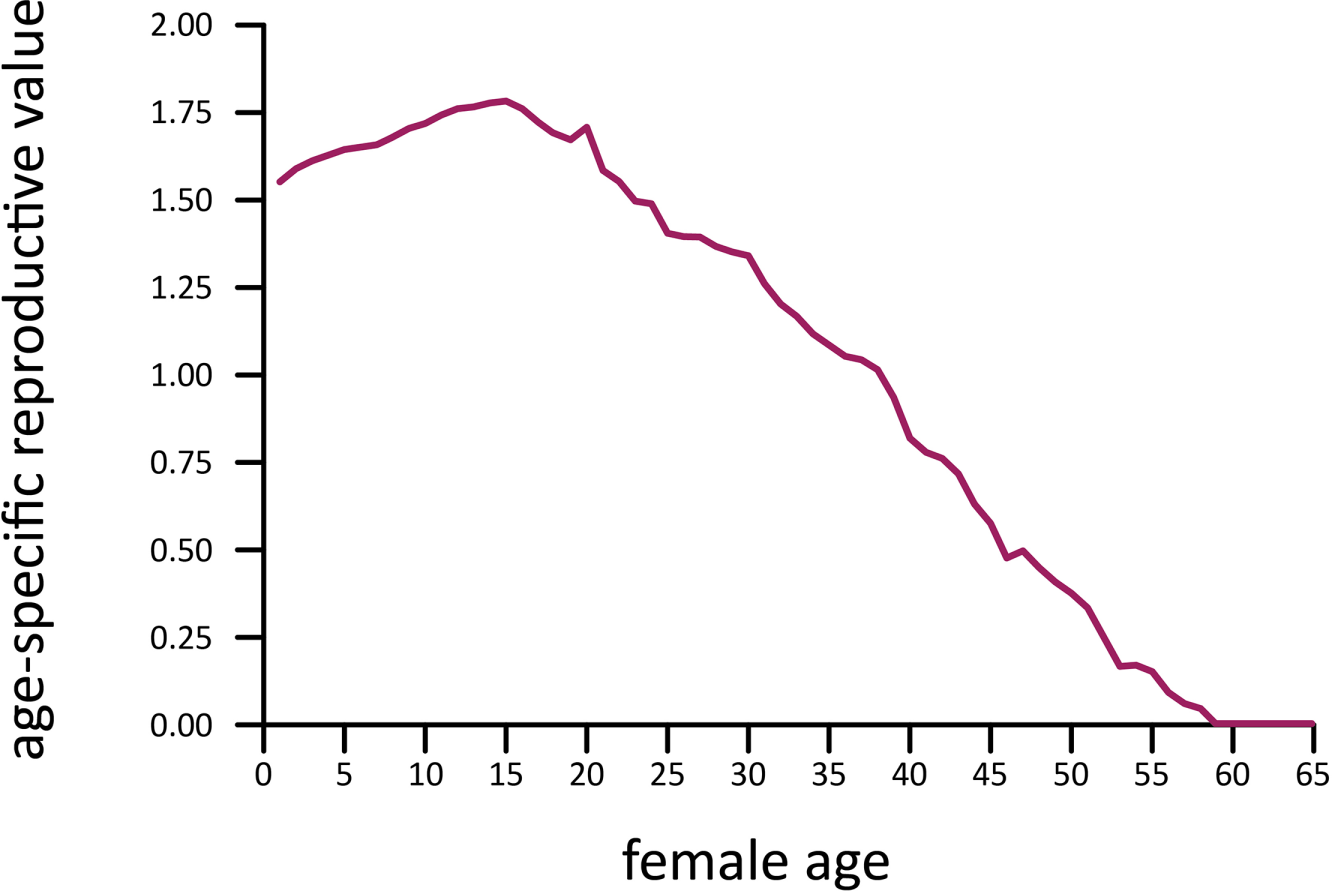
Female reproductive value.

Age-specific fecundity, measured as the number of female calves produced by females of a given age, averaged 0.06 female calves per female-year for females between the ages of 10 and 60 (Fig 5). Females of primiparous age (10–23 yrs) averaged 0.04 calves/year, mature females (24–49 yrs.) 0.07 calves/year, and oldest females (>49 yrs.) were similar to the youngest age group at 0.04 calves/year. Overall fecundity was fairly stable from the age of 20 to 45. The oldest female to give birth during the study was 59 years old and the youngest was 10. Reproductive value of females peaked between 15 and 25 years of age and then decreased linearly (Fig 6).

Births among older females drop off markedly. Of a sample of 38 females older than 49 years (cumulative observation time 4025 months), 26 females gave birth to 31 calves, half the number expected given the average inter-birth interval. The oldest known female when she died was estimated to be 75 years old. Caution is advisable with this age-class because all of these females were mature at the time they were first identified, and the accuracy of aging is relatively poor.

## Discussion

Effective conservation strategies are founded on evidence-based knowledge of rates of population change, levels of human-induced mortality, and the natural demographic processes that determine how populations will respond to severe size reductions [3]. In this paper we provide age-specific measures of demographic processes collected over nearly 20 years in a forest elephant population, a cryptic species for which basic biological knowledge is lacking despite its iconic status. These data complement previous analyses of change in population size and age-sex distribution [21], and cohort-based estimates of annual birth and death rates, inter-birth intervals, and age of females to first reproduction [6]. These new estimates reinforce the substantial difference in demographic indices compared to those of well-studied savannah elephant populations, highlighting the unique biology of forest elephants. Importantly, these age specific metrics help identify differential mortality risk and reproductive value across age classes, information critical for diagnosing and reversing population decline, and projecting population status and trends.

### Basic demographics

For the analysis period 1997–2010, births averaged 5.3% and mortality 3.5% of the annual population known to be alive. This differs from the numbers reported previously [6] because of changes in cohort composition related to immigration. Specifically, in the previous analysis we focused on a static cohort through time, while in this manuscript we included all individuals with detailed data, regardless of when they entered the known cohort. Thus, the intrinsic growth rate reported from the static cohort analyzed in [6] of 1.9% is more stringently defined and we avoid reporting intrinsic growth in this analysis due to the impacts of migration on the sample (though ignoring this constraint results in similar metrics). On average net migration (immigration—emigration) added 1% annually to population growth. Immigration to the Dzanga system (mean 2.5% annually) may reflect the relative safety of the ecosystem (supported by Monitoring of Illegal Killing of Elephants data[6]), and/or problems in surrounding areas (logging concessions were active in surrounding forests the duration of the study which may influence immigration).

Well-studied savannah elephant populations show variable rates of mortality and natality, driven by environmental factors. For two well studied populations, in Amboseli and Samburu, Kenya, annual mortality and natality oscillated strongly between near 0 and over 10% per year. Average annual mortality was higher than that seen in Dzanga, at 4.15% and 4.7% respectively [11], which in part was due to the influence of major droughts that happened infrequently (i.e., 5–15 years) in the savannah systems [11, 25]. Forest elephant annual mortality was lower (3.5%) and less variable, likely in relation to more stable environmental conditions and possibly influenced by the lack of big predators in that system. Concerns in distinguishing dispersal from death affect both this study and those of savanna elephants.

### Variation in reproduction: Age differences and seasonality

The ecology of primary tropical forests for large, ground dwelling herbivores likely influences their intrinsic growth rates, and has been speculated to drive the reduced fecundity in the study population [6]. While thought to experience much less seasonal variation relative to savanna systems, at least in terms of water availability and vegetative productivity, weak seasonality in reproduction was found among the Dzanga elephants. Specifically, the frequency of conceptions peaked during the middle of the rainy season, between May and August (Fig 2), indicating seasonal signals in reproduction. Notably, while this seasonal effect was significant, we did not find a significant correlation between the month specific conception and precipitation. Studies in the neighboring Nouabalé-Ndoki National Park suggest fruit abundance in these forests is highest during periods of higher rainfall [28] which might correlate with the seasonality of conception in the Dzanga population. Interestingly, the sex ratio of new born calves did not deviate from 1:1 regardless of year or season of conception, suggesting no sex-biased maternal investment [29]. The fact that births occurred throughout the year differs substantially from savannah elephant systems, where annual variation in the abundance and quality of food resources, caused by seasonal precipitation and related vegetative productivity, strongly structure reproductive biology and survival [24, 25, 30, 31]. This difference in the inter-annual reproductive rate between elephant species has a profound effect on population demography, resulting in a more uniform expected age structure in forest elephants over time.

Median birth interval for a sample of 212 females where sequential births were observed was 59 months (SD = 18.4, range 20–136), but there was considerable variation in the interval that was predominantly explained by female age. Relative to females in their ‘prime’, females younger than 25 years and females older than 39 years had longer intervals, with the median inter-calf interval of females in the older age-class being 11 months longer than those of prime aged females. In contrast to savannah elephant populations, where variation in intervals is mostly explained by calf survivorship [32], we found only weak evidence that calf survival influenced inter-calf intervals in forest elephants, largely due to high calf survival. When calves survived past 24 months, savannah elephant females waited about 53 months to bear the next calf and even the oldest females generally gave birth again after a wait of 63 months [32]. The equivalent wait for older forest elephant females was 75 months. Surprisingly, we found no evidence that inter-calf intervals were influenced by sex and birth order in forest elephants, in contrast to findings for savannah elephants [32]. The longer inter-calving interval combined with delayed age of primiparity (median 23 years), result in a markedly slower intrinsic growth rate for the Dzanga elephant population relative to savanna elephant populations. This difference is suspected to be related to the challenges of being a terrestrial herbivore in primary rainforest due to tannins in forage and productivity occurring predominantly in the canopy [6]. In combination, these rates result in one of the longest generation times (i.e., the time between a females birth and the birth of her first female offspring) recorded among mammals, estimated to be 31 years for Dzanga, compared to 24.1 years for Samburu elephants using the same methods [11]. Combined with heavy poaching pressure, these demographic rates present challenges for the recovery of forest elephant populations even with adequate protection [6].

### Age specific survivorship

Survivorship was high among juvenile Dzanga elephants through the age of 13, irrespective of sex (Figs 3 and 4). The transition in survivorship around the age of 13 may be linked to natal dispersal with the median age of dispersal being 15 years. It is important to note that complete independence from the natal group typically did not occur for months or years after the initial break, with repeated affiliation observed among dispersing individuals and their mothers. It is also notable that the median age of dispersal for females is much younger than the median age of primiparity. This prenatal dispersal among female forest elephants is notably different from the strong female philopatry and matrilocality in their savanna elephant relatives, for which familial support in the rearing of calves is thought to be a primary driver of social philopatry [33].

After the age of 13 the survivorship probability of males begins to decrease relative to females. It is notable that the survival estimates between the ages of 13 and about 20 are not well characterized on account of dispersal from the natal group. In particular, male estimates arebased on relatively few individuals that frequently visited the bai and could be individually identified through this age-span in absence of family context. We assume that dispersal behavior incurs increased mortality risk to young males as they spend more time alone. Our censoring of 'lost' males and focus on bai residents potentially inflates our survival estimates. As such, we believe the survival curves over this period are conservative, underestimating mortality because most individuals that disappeared in the sample were censored.

These survival probabilities, particularly of young animals, are strikingly different from those estimated for savannah populations. Although the age of male dispersal is similar in the two species, 19% of savannah calves in Amboseli [32] and 12% in Samburu [11] died before they were two years old; much higher than the 3% observed for forest elephants. In the savannah, droughts and predator mortality (lions, hyenas) disproportionately impact calves [11, 34], but for the most part these mortality factors are absent from the forest ecosystems of Central Africa. This may contribute to the relatively higher calf survivorship recorded at Dzanga. The major threat to survival in forest elephants is poaching, which potentially drives differential survivorship between the sexes (females were nearly twice as likely to survive to a given age as males over their lifetimes) given that males are primary targets for ivory harvest.

The pattern of age-specific fecundity for forest elephant females (Fig 5), and similar estimates from two populations of savannah elephants [11, 25], are remarkably similar, differing primarily in the onset of reproduction (age of primiparity) and an overall lower level through a forest elephant female’s lifetime because of the long inter-birth interval. We could not discern any change in reproductive value for females in older age-classes as was found in savannah elephants [11, 25].

### Study system considerations

While the rarity of detailed data on forest elephants makes the data and analyses presented here invaluable with respect to understanding forest elephant biology, it is important to recognize aspects of the study system that potentially influence these results. The study population is comprised of individuals that choose to visit the Dzanga clearing during the day, potentially from a much larger population with individuals that are not observed. Thus, our dataset is comprised of ‘self-selected’ individuals (see description in S1 Text). Observations were limited to daylight hours, primarily in the afternoon. It is possible such diurnal observation leads to some sort of bias in sampling (e.g. lower observation rates of at risk individuals like large male tuskers), although a three-week thermal imaging study provided no evidence that any age-class preferentially entered the clearing only at night.

Forty-four percent of potentially new individuals either were observed only a single time or problematic to identify (no discernable markings) [21] and, therefore, could not be included in analyses. While we have no reason to believe the included cohort of individuals was not representative, it is clear this is not a complete sampling of the population. The constant influx of new adults, including female family groups, supports the conclusion that the observed age and sex structure is determined in part by immigration. We took care to define our core cohort of individuals based on criteria that would include as many individuals as possible whose identification and ages were confidently known (see criteria listed in methods). In order to perform a sensitivity analysis of these criteria, we relaxed most of these selection criteria and re-examined survival estimates without finding strong differences in results (survival estimates) or unexpected bias (S1 Text).

We suggest that the demography of the Dzanga population might not be representative of other forest elephant populations as much as might be desired, because the Dzanga population has experienced better protection than other populations (e.g. based on the Monitoring of Illegal Killing of Elephants data [16] and survey data [15]). The demographic metrics presented here are therefore likely to be representative of a population under relatively good protection.

Finally, it was not possible to diagnose the causes of disappearances of individuals that were socially independent, particularly older males. We used social information to the greatest extent possible to differentiate dispersal (resulting in censorship of that individual) versus mortality. But after the age of 15 for males, social criteria were no longer reliable. As such, survival rates reported for older individuals, particularly males, are apparent survival rates given the potential misspecification between mortality and dispersal.

### Conclusion

This detailed demographic study of a forest elephant population diagnoses the drivers of the observed marked differences in intrinsic growth potential and generation time between forest and savannah elephants [6]. In concordance with the IUCN Red List criteria for assessing population changes [35], the conservation status of forest elephants should be assessed using a generation time of 31 years (i.e. for 62 or 93 years). However, survey data are not available over this time frame. Rather, contemporary data demonstrates drastic declines over the last 20–30 years (one generation) [15, 36, 37]. As such, IUCN species assessments using historic data should be based on this period.

With the ongoing scenario, forest elephant populations are in crisis and show limited recovery potential even with an optimistic view of adequate protection. In several savannah elephant populations, marked recoveries in elephant numbers have been recorded after severe drought and intensive poaching, driven notably by a lowering of the primiparous age and inter-birth interval [11, 23]. Concerningly, we found no evidence of this compensatory response in the Dzanga population despite evidence of poaching [6]. Nor do we have evidence of adequate protection in their range needed prior to recovery.

## Supporting information

S1 Text Sensitivity analysis of cohort definition. (DOCX)

S1 Table Annual number of individuals, births, deaths, dispersals and immigrants used in analyses. (DOCX)

S2 Table Individual based survivorship data. (DOCX)

S3 Table Individual based inter-calf interval data. (DOCX)

## References

[1] Roff DA. The Evolution of Life Histories: Theory and Analysis. New York: Chapman & Hall, Inc; 1992.

[2] Stearns SC. The Evolution of Life Histories. Oxford, U.K.: Oxford University Press; 1992.

[3] Caughley G, Sinclair A. Wildlife Ecology and Management. Cambridge, MA: Blackwell Science; 1994.

[4] Gaillard JM, Festa-Bianchet M, Yoccoz NG. Population dynamics of large herbivores: variable recruitment with constant adult survival. Trends in Ecology & Evolution. 1998; 13(2):58–63. doi: 10.1016/s0169-5347(97)01237-8 WOS:000071817100007. 21238201 10.1016/s0169-5347(97)01237-8

[5] Brooks ME, Mugabo M, Rodgers GM, Benton TG, Ozgul A. How well can body size represent effects of the environment on demographic rates? Disentangling correlated explanatory variables. J Anim Ecol. 2016; 85(2):318–28. doi: 10.1111/1365-2656.12465 WOS:000370959300002. 26620593 10.1111/1365-2656.12465

[6] Turkalo AK, Wrege PH, Wittemyer G. Slow intrinsic growth rate in forest elephants indicates recovery from poaching will require decades. Journal of Applied Ecology. 2017; 54(1):153–9. doi: 10.1111/1365-2664.12764

[7] Royle JA, Nichols JD. Estimating abundance from repeated presence-absence data or point counts. Ecology. 2003; 84(3):777–90. doi: 10.1890/0012-9658(2003)084[0777:eafrpa]2.0.co;2 WOS:000181629500029.

[8] White GC, Burnham KP. Program MARK: survival estimation from populations of marked animals. Bird Study. 1999; 46:120–39. WOS:000084390400015.

[9] Schwartz MK, Luikart G, Waples RS. Genetic monitoring as a promising tool for conservation and management. Trends in Ecology & Evolution. 2007; 22(1):25–33. doi: 10.1016/j.tree.2006.08.009 WOS:000243552900008. 16962204 10.1016/j.tree.2006.08.009

[10] Clutton-Brock T, Sheldon BC. Individuals and populations: the role of long-term, individual-based studies of animals in ecology and evolutionary biology. Trends in Ecology & Evolution. 2010; 25(10):562–73. doi: 10.1016/j.tree.2010.08.002 ISI:000282860600005. 20828863 10.1016/j.tree.2010.08.002

[11] Wittemyer G, Daballen D, Douglas-Hamilton I. Comparative Demography of an At-Risk African Elephant Population. PLoS One. 2013; 8(1). e53726 doi: 10.1371/journal.pone.0053726 23341984 10.1371/journal.pone.0053726 PMC3547063

[12] Blake S, Strindberg S, Boudjan P, Makombo C, Bila-Isia I, Ilambu O, et al Forest elephant crisis in the Congo Basin. PLoS Biology. 2007; 5(4):e111 doi: 10.1371/journal.pbio.0050111 17407383 10.1371/journal.pbio.0050111 PMC1845159

[13] Ishida Y, Oleksyk TK, Georgiadis NJ, David VA, Zhao K, Stephens RM, et al Reconciling Apparent Conflicts between Mitochondrial and Nuclear Phylogenies in African Elephants. PLoS One. 2011; 6(6). e20642 doi: 10.1371/journal.pone.0020642 WOS:000291611500036. 21701575 10.1371/journal.pone.0020642 PMC3110795

[14] Roca AL, Ishida Y, Brandt AL, Benjamin NR, Zhao K, Georgiadis NJ. Elephant Natural History: A Genomic Perspective. Annual Review of Animal Biosciences. 2015; 3(1):139–67. doi: 10.1146/annurev-animal-022114-110838 25493538 10.1146/annurev-animal-022114-110838

[15] Maisels F, Strindberg S, Blake S, Wittemyer G, Hart J, Williamson EA, et al Devastating decline of forest elephants in Central Africa. PLoS ONE. 2013; 8(3):e59469 Epub 3/4/2013. doi: 10.1371/journal.pone.0059469 23469289 10.1371/journal.pone.0059469 PMC3587600

[16] Wittemyer G, Northrup JM, Blanc J, Douglas-Hamilton I, Omondi P, Burnham KP. Illegal killing for ivory drives global decline in African elephants. Proceedings of the National Academy of Sciences of the United States of America. 2014; 111(36):13117–21. doi: 10.1073/pnas.1403984111 25136107 10.1073/pnas.1403984111 PMC4246956

[17] Mayaux P, Pekel JF, Desdee B, Donnay F, Lupi A, Achard F, et al State and evolution of the African rainforests between 1990 and 2010. Philosophical Transactions of the Royal Society B-Biological Sciences. 2013; 368(1625). doi: 10.1098/rstb.2012.0300 23878331 10.1098/rstb.2012.0300 PMC3720022

[18] Beaune D, Bretagnolle F, Bollache L, Hohmann G, Surbeck M, Fruth B. Seed dispersal strategies and the threat of defaunation in a Congo forest. Biodiversity and Conservation. 2013; 22(1):225–38. doi: 10.1007/s10531-012-0416-x

[19] Blake S, Deem SL, Mossimbo E, Maisels F, Walsh P. Forest elephants: tree planters of the Congo. Biotropica. 2009; 41(4):459–68. doi: 10.1111/j.1744-7429.2009.00512.x ISI:000267538200009.

[20] Blake S, Inkamba-Nkulu C. Fruit, minerals, and forest elephant trails: Do all roads lead to Rome? Biotropica. 2004; 36(3):392–401. ISI:000223742800013.

[21] Turkalo AK, Wrege PH, Wittemyer G. Long-term monitoring of Dzanga Bai forest elephants: forest clearing use patterns. PloS ONE. 2013; 8(12):e85154 Epub 12/26/2013. doi: 10.1371/journal.pone.0085154 24386460 10.1371/journal.pone.0085154 PMC3873458

[22] Douglas-Hamilton I. On the ecology and behaviour of the African elephant: Elephants of Lake Manyara [D.Phil.]. Oxford: Oxford University; 1972.

[23] Foley CAH, Faust LJ. Rapid population growth in an elephant *Loxodonta africana* population recovering from poaching in Tarangire National Park, Tanzania. Oryx. 2010; 44(2):205–12. doi: 10.1017/s0030605309990706 WOS:000277951400014.

[24] Gough KF, Kerley GIH. Demography and population dynamics in the elephants *Loxodonta africana* of Addo Elephant National Park, South Africa: is there evidence of density dependent regulation? Oryx. 2006;4 0(4):434–41. doi: 10.1017/s0030605306001189 WOS:000243833100016.

[25] Moss CJ. The demography of an African elephant (*Loxodonta africana*) population in Amboseli, Kenya. Journal of Zoology. 2001; 255:145–56. ISI:000171784700002.

[26] Wrege PH, Rowland ED, Bout N, Doukaga M. Opening a larger window onto forest elephant ecology. African Journal of Ecology. 2012; 50(2):176–83. doi: 10.1111/j.1365-2028.2011.01310x

[27] Turkalo AK. Estimating forest elephant age. African Journal of Ecology. 2013 doi: 10.1111/aje.12087

[28] Blake S. The ecology of forest elephant distribution and its implications for conservation [Ph.D.]. Edinburgh: University of Edinburgh; 2002.

[29] Hewison AJM, Gaillard JM. Successful sons or advantaged daughters? The Trivers-Willard model and sex-biased maternal investment in ungulates. Trends in Ecology & Evolution. 1999; 14(6):229–34. doi: 10.1016/s0169-5347(99)01592-x WOS:000081381300009. 10354625 10.1016/s0169-5347(99)01592-x

[30] Foley CAH, Pettorelli N, Foley L. Severe drought and calf survival in elephants. Biology Letters. 2008; 4:541–4. doi: 10.1098/rsbl.2008.0370 18682358 10.1098/rsbl.2008.0370 PMC2610102

[31] Wittemyer G, Rasmussen HB, Douglas-Hamilton I. Breeding phenology in relation to NDVI variability in free-ranging African elephant. Ecography. 2007; 30(1):42–50. ISI:000244419200005.

[32] Moss CJ, Lee PC. Female reproductive strategies: individual life histories. Moss CJ, Croze H, Lee PC, editors 2011. 187–204 p.

[33] Wittemyer G, Douglas-Hamilton I, Getz WM. The socioecology of elephants: analysis of the processes creating multitiered social structures. Animal Behaviour. 2005; 69:1357–71. ISI:000230036400014.

[34] Wittemyer G, Daballen DK, Rasmussen HB, Kahindi O, Douglas-Hamilton I. Demographic Status of elephants in the Samburu and Buffalo Springs National Reserves, Kenya. African Journal of Ecology. 2005; 43:44–7.

[35] Mace GM, Collar NJ, Gaston KJ, Hilton-Taylor C, Akcakaya HR, Leader-Williams N, et al Quantification of Extinction Risk: IUCN's System for Classifying Threatened Species. Conserv Biol. 2008; 22(6):1424–42. doi: 10.1111/j.1523-1739.2008.01044.x WOS:000261395700018. 18847444 10.1111/j.1523-1739.2008.01044.x

[36] Bouche P, Douglas-Hamilton I, Wittemyer G, Nianogo AJ, Doucet JL, Lejeune P, et al Will Elephants Soon Disappear from West African Savannahs? PLoS One. 2011; 6(6):e20169. e20619. 21731620 10.1371/journal.pone.0020619 PMC3120750

[37] Beyers RL, Hart JA, Sinclair ARE, Grossmann F, Klinkenberg B, Dino S. Resource Wars and Conflict Ivory: The Impact of Civil Conflict on Elephants in the Democratic Republic of Congo—The Case of the Okapi Reserve. PLoS One. 2011; 6(11). doi: 10.1371/journal.pone.0027129 WOS:000297350800030. 22096529 10.1371/journal.pone.0027129 PMC3212536

