# Supplementary material for: Demography of a forest elephant population
Source: PLoS One. 2018 Feb 15;13(2):e0192777. doi: 10.1371/journal.pone.0192777 (PMC5813957; doi:10.1371/journal.pone.0192777)
Supplement: S1 Text — (DOCX) [file pone.0192777.s001.docx]

**Supporting Information**

**Demography of a Forest Elephant population**

Andrea K. Turkalo, Peter H. Wrege, George Wittemyer

**S1 Text. Sensitivity analysis of cohort definition**

**Investigating potential bias**

The foundation of demographic analyses presented in this paper are the long-term, individual-based observations made by AKT on forest elephants visiting the Dzanga Bai in the south of the Central African Republic. With nearly 1000 individually recognized elephants visiting every year, Dzanga Bai is by far the most active elephant clearing known. In spite of this high visiting rate, dung transects in the surrounding forest suggest a relatively low population density [1], which leads us to assume that many individuals are traveling from considerable distance to visit the bai. Our sample therefore represents a ‘self-selected’ set of individuals and there is no reasonable way to know whether it is representative of the larger source population. The major difference from other forest elephant populations may be that Dzanga is situated within a UNESCO world heritage site, the Sangha Trinational, and has perhaps enjoyed more protection than many other populations.

Below we present analysis results after modifying selection criteria for included individuals. But in order to understand these investigations, we first detail our motivation for choosing the criteria that we did.

The frequency of sightings of individuals at Dzanga is highly variable, but on average an individual or family visits the clearing only 2-3 times per year, for a few days each visit [2]. More than 50% of potentially unique individuals are seen only a few times, either because they visit extremely rarely (including missed sightings because of visits at night or when an observer was not present), or were not sufficiently distinct to ensure recognition on later entrance. For these reasons a set of criteria for inclusion in the demographic datasets were formulated *a priori* to focus on individuals and families that were best recognized and whose visiting behavior to the Dzanga bai was frequent enough to provide confidence that disappearances were likely mortality events and not dispersal [2, 3]. The most basic of these criteria was that an individual had to be re-sighted nine or more months after its first identification to ensure that it was, in fact, individually recognizable. Three additional criteria were applied, based on the distribution of sightings for all individuals seen at least twice over nine months: (1) sighted a minimum of 12 times during the 20-yr study, (2) sighted an average of at least four times per year, and (3) sighted, if an adult, for at least three years.

In any demographic analysis involving wild populations, one of the most difficult decisions is whether to code a permanent absence as a mortality event or a dispersal event (in the latter case the observation is ‘censored’ in survival analyses). For elephants, proof of mortality is rare and so mortality is usually inferred with corroborating data the rest of the family group, consistency of sightings, etc. This decision is particularly difficult in the case of forest elephants because they generally cannot be observed or searched for away from the clearing, nor can the family group be located to confirm an absence short of their next visit to the bai. For this reason, we integrated the average time between successive visits to the clearing and social information, when available, to decide on a time-point for mortality events.

For our demographic analyses, we implemented the above criteria on frequency of observation largely in the hope of identifying a primarily ‘resident’ sub-population. Adults that disappeared permanently were assumed to have died. We required an individual be absent for four times its average inter-visit interval before assigning a mortality date at twice the inter-visit interval following its last sighting. For dependent offspring, the required frequency of group observation allowed us to use social information to inform whether a disappeared calf, depending on age and sex, might reasonably have dispersed rather than died.

In order to investigate the possibility that our analysis set contains unintended bias, we constructed an expanded set of observations that relaxed all of the criteria listed above except that we had to be certain of unique identification (was seen at minimum nine months after initial identification). Surprisingly this added only 202 new individuals to the ‘core’ dataset of 1563. However, the characteristics of these ‘added’ individuals were generally quite different from the core set, as shown in table S1.1. In general, differences in visiting interval and the proportion of individuals that were still alive at the end of the 20-yr study (= censored for survival analysis) are what would be expected by including individuals that were rarely seen.

| Table S1.1. Frequency of demographic characteristics in two samples from the Dzanga population. The ‘core’ dataset satisfied all three criteria associated with sighting frequency, while the ‘added’ cases only required unique identification. | | |
| --- | --- | --- |
|  | Core Dataset | 202 added cases |
| average number of days (sd) between successive visits | 123 (166) | 2205 (25815) |
| Enters as calf or juvenile | 69% | 77% |
| Dead (not censored) - all ages | 28% | 46% |
| Dead (not censored) – juveniles only | 17% | 41% |
| Younger than 5yrs when last seen (= dead) | 23% | 46% |

The standard deviation of visit interval for the added cases indicates that some individuals were unobserved for very long periods, but on average these new individuals were seen for significantly fewer years than the core dataset, 6.7 years versus 10.9 years, respectively (GLM controlling for sex, F_1,1763_ = 90.83, p<0.001).

We ran four different Cox regression models (proportional hazards) with staggered entry times, using different dataset criteria. We compared (1) the ‘core’ dataset used for analyses in this paper, (2) the more restricted dataset of individuals representing a ‘cohort’ of adults analyzed in a previous paper [3], differing primarily by the absence of immigrants arriving during the analysis period, (3) an inclusive dataset that combined the ‘core’ and the ‘relaxed’ individuals, and (4) a dataset comprised only of the 202 ‘added’ cases. It is important to note that dataset (2) overlaps by 75% with dataset (1) and particularly that the added cases used in dataset (3) represent only 11% of observations and so their influence on the results are expected to be overwhelmed by the ‘core’ observations.

Figure S1.1 shows the survivorship curves for males (as an example), estimated using each of these datasets. Except for the dataset using only the 202 added individuals, the different datasets produced qualitatively very similar estimates of male survival, all within the 95% CI for the ‘core’ analysis. In fact, the hazard ratios indicating the relative increased survival of females relative to males were 0.56, 0.61, and 0.61 for datasets (1), (2), and (3), respectively. Analyzed alone, the sample of 202 added cases estimated a much steeper survival curve and there was no significant difference between males and females in that sample.


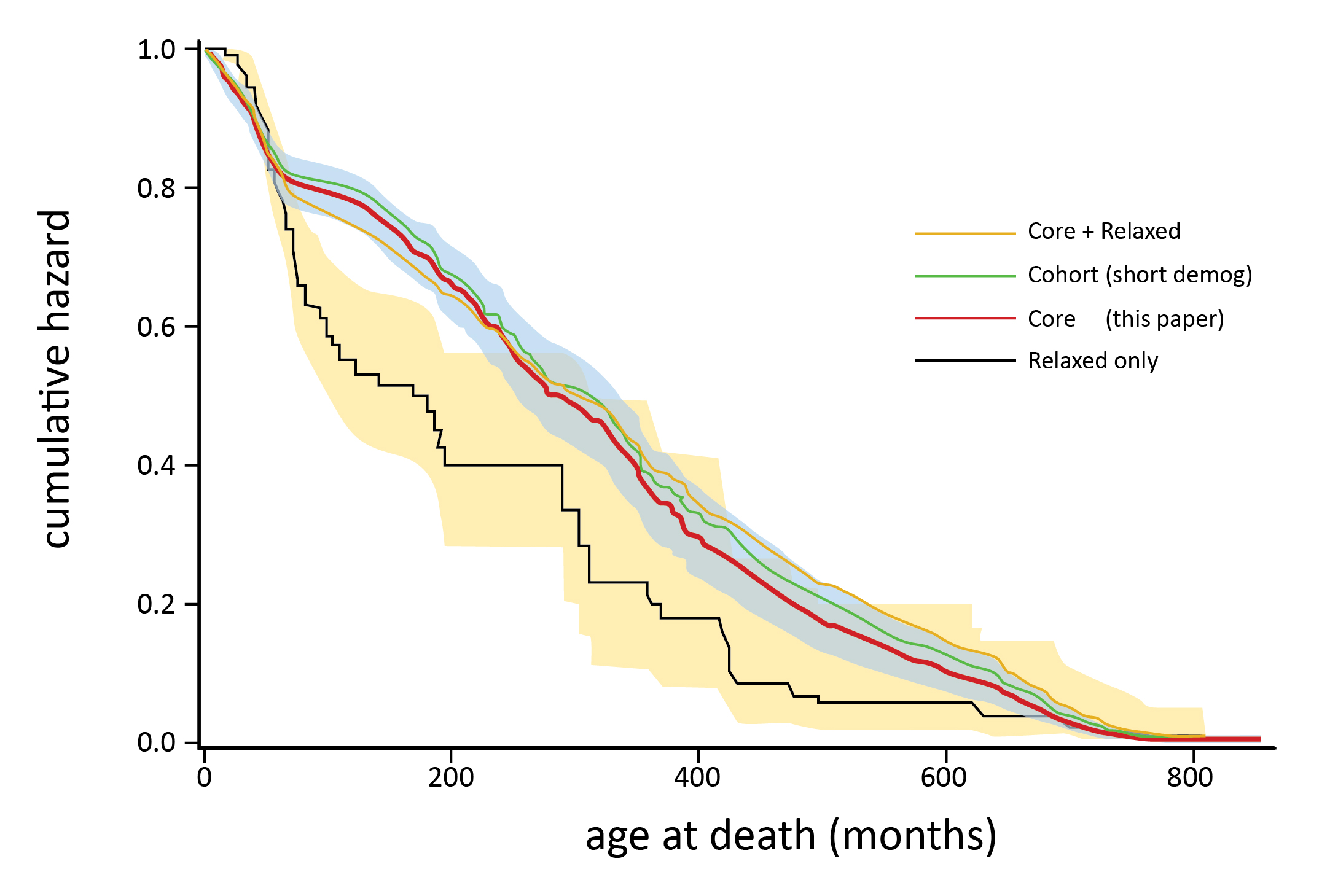


Figure S1.1. Comparison of Cox regression analyses with alternative datasets. All include staggered entry to the risk set, with sex as the only covariate. Plots are of male survival. Blue region indicates 95% point-wise CI for the Core regression, the yellow area for the relaxed cases.

References:

1. African Elephant Database [Internet]. 2013 [cited 1 July 2016]. Available from: <http://www.elephantdatabase.org>.

2. Turkalo AK, Wrege PH, Wittemyer G. Long-term monitoring of Dzanga Bai forest elephants: forest clearing use patterns. PloS ONE. 2013;8(12):e85154. Epub 12/26/2013. doi: 10.1371/journal.pone.0085154.

3. Turkalo AK, Wrege PH, Wittemyer G. Slow intrinsic growth rate in forest elephants indicates recovery from poaching will require decades. Journal of Applied Ecology. 2017;54(1):153-9. doi: 10.1111/1365-2664.12764.
