# Supplementary material for: Demography of a forest elephant population
Source: PLoS One. 2018 Feb 15;13(2):e0192777. doi: 10.1371/journal.pone.0192777 (PMC5813957; doi:10.1371/journal.pone.0192777)
Supplement: S1 Table — (DOCX) [file pone.0192777.s002.docx]

**Supporting Information**

**Demography of a Forest Elephant population**

Andrea K. Turkalo, Peter H. Wrege, George Wittemyer

S2 Table: Annual number of individuals, births, deaths, dispersals and immigrants used in analyses

| studyyr | alive_start | alive_end | dead | born | dispersed | immigrant |
| --- | --- | --- | --- | --- | --- | --- |
| 1 | 0 | 53 | 0 | 36 | 0 | 17 |
| 2 | 53 | 601 | 4 | 59 | 0 | 493 |
| 3 | 601 | 1026 | 28 | 32 | 1 | 421 |
| 4 | 1026 | 1319 | 62 | 53 | 0 | 302 |
| 5 | 1319 | 1427 | 103 | 56 | 0 | 155 |
| 6 | 1427 | 1492 | 89 | 56 | 5 | 98 |
| 7 | 1492 | 1514 | 104 | 64 | 9 | 62 |
| 8 | 1514 | 1514 | 94 | 44 | 9 | 50 |
| 9 | 1514 | 1554 | 97 | 56 | 14 | 81 |
| 10 | 1554 | 1540 | 129 | 81 | 21 | 34 |
| 11 | 1540 | 1511 | 100 | 54 | 20 | 17 |
| 12 | 1511 | 1466 | 124 | 65 | 20 | 14 |
| 13 | 1466 | 1453 | 113 | 44 | 16 | 56 |
| 14 | 1453 | 1436 | 127 | 45 | 24 | 65 |
| 15 | 1436 | 1444 | 51 | 35 | 5 | 24 |
| 16 | 1444 | 1462 | 72 | 56 | 13 | 34 |
| 17 | 1462 | 1503 | 63 | 60 | 13 | 44 |
| 18 | 1503 | 1581 | 54 | 52 | 16 | 80 |
| 19 | 1581 | 1613 | 59 | 50 | 17 | 41 |
| 20 | 1613 | 1642 | 17 | 42 | 4 | 4 |
