# Supplementary material for: Demography of a forest elephant population
Source: PLoS One. 2018 Feb 15;13(2):e0192777. doi: 10.1371/journal.pone.0192777 (PMC5813957; doi:10.1371/journal.pone.0192777)
Supplement: S2 Table — (DOCX) [file pone.0192777.s003.docx]

**Supporting Information**

**Demography of a Forest Elephant population**

Andrea K. Turkalo, Peter H. Wrege, George Wittemyer

S3 Table. Individual based survivorship data

| eleid | sex | birthdate | FirstObs | LastObs | diedate | startdate | termdate | censored |
| --- | --- | --- | --- | --- | --- | --- | --- | --- |
| 4 | F | 1/1/1935 | 10/9/1990 | 11/12/2011 | 6/23/2012 | 10/8/1993 | 5/31/2010 | 1 |
| 5 | F | 1/1/1986 | 10/9/1990 | 11/12/2011 | 6/27/2012 | 10/8/1993 | 5/31/2010 | 1 |
| 6 | M | 10/1/1984 | 10/9/1990 | 7/25/1997 | 10/5/1997 | 10/8/1993 | 10/5/1997 | 1 |
| 7 | F | 4/25/1994 | 6/17/1994 | 8/8/1997 | 10/15/1997 | 4/25/1994 | 10/15/1997 | 0 |
| 8 | F | 1/1/1964 | 10/26/1990 | 6/18/2002 | 9/14/2002 | 10/25/1993 | 9/14/2002 | 0 |
| 10 | F | 1/1/1965 | 10/26/1990 | 7/15/2001 | 10/11/2001 | 10/25/1993 | 10/11/2001 | 0 |
| 11 | M | 1/1/1983 | 10/26/1990 | 12/11/2004 | 4/8/2005 | 10/25/1993 | 4/8/2005 | 0 |
| 13 | M | 12/9/1990 | 1/8/1991 | 1/2/2002 | 4/2/2002 | 12/9/1990 | 4/2/2002 | 0 |
| 14 | F | 4/19/1994 | 6/25/1994 | 2/14/2011 | 7/10/2011 | 6/25/1994 | 5/31/2010 | 1 |
| 15 | F | 7/1/1997 | 7/5/1997 | 7/27/2009 | 1/15/2010 | 7/1/1997 | 1/15/2010 | 0 |
| 22 | F | 1/1/1969 | 12/28/1990 | 11/8/2003 | 3/29/2004 | 12/27/1993 | 3/29/2004 | 0 |
| 23 | M | 1/1/1985 | 12/28/1990 | 8/8/2000 | 11/24/2000 | 12/27/1993 | 11/24/2000 | 0 |
| 24 | F | 9/3/1991 | 12/2/1991 | 2/26/2013 |  | 12/2/1991 | 5/31/2010 | 1 |
| 25 | F | 6/15/1997 | 10/2/1997 | 12/15/2000 | 4/26/2001 | 10/2/1997 | 4/26/2001 | 0 |
| 28 | M | 1/1/1965 | 9/19/1991 | 1/10/2013 |  | 9/18/1994 | 5/31/2010 | 1 |
| 33 | M | 1/1/1941 | 2/25/1991 | 3/9/2000 | 10/19/2000 | 2/24/1994 | 10/19/2000 | 0 |
| 35 | M | 1/1/1960 | 6/4/1991 | 2/26/2013 |  | 6/3/1994 | 5/31/2010 | 1 |
| 40 | M | 1/1/1966 | 1/18/1991 | 3/9/2004 | 3/14/2005 | 1/17/1994 | 3/14/2005 | 0 |
| 44 | F | 1/1/1980 | 9/19/1992 | 5/24/1998 | 10/17/1998 | 9/19/1995 | 10/17/1998 | 0 |
| 50 | M | 1/1/1958 | 2/13/1993 | 3/13/2000 | 6/23/2000 | 2/13/1996 | 6/23/2000 | 0 |
| 52 | F | 1/1/1988 | 3/24/1991 | 11/28/2012 |  | 3/23/1994 | 5/31/2010 | 1 |
| 53 | M | 6/1/1994 | 7/9/1994 | 8/7/2009 | 5/12/2010 | 6/1/1994 | 5/12/2010 | 0 |
| 54 | M | 4/1/2000 | 10/30/2000 | 10/4/2012 |  | 10/30/2000 | 5/31/2010 | 1 |
| 55 | F | 8/1/2006 | 8/16/2006 | 10/4/2012 |  | 8/1/2006 | 5/31/2010 | 1 |
| 69 | F | 1/10/1960 | 1/13/1991 | 3/22/2013 |  | 1/12/1994 | 5/31/2010 | 1 |
| 70 | F | 1/1/1985 | 9/4/1991 | 3/22/2013 |  | 1/12/1994 | 5/31/2010 | 1 |
| 71 | F | 12/1/1992 | 12/27/1992 | 12/13/2010 | 4/30/2011 | 12/1/1992 | 5/31/2010 | 1 |
| 72 | M | 11/15/2001 | 3/24/2002 | 2/7/2013 |  | 3/24/2002 | 5/31/2010 | 1 |
| 75 | F | 9/15/1998 | 9/16/1998 | 1/13/2013 |  | 9/15/1998 | 5/31/2010 | 1 |
| 76 | M | 3/1/2003 | 8/20/2003 | 12/28/2011 | 4/16/2012 | 8/20/2003 | 5/31/2010 | 1 |
| 77 | M | 6/23/2007 | 8/31/2007 | 3/22/2013 |  | 8/31/2007 | 5/31/2010 | 1 |
| 84 | M | 1/1/1951 | 6/5/1991 | 8/30/1996 | 5/27/1997 | 6/4/1994 | 5/27/1997 | 0 |
| 86 | M | 1/1/1951 | 2/3/1991 | 8/5/2006 | 2/23/2007 | 2/2/1994 | 2/23/2007 | 0 |
| 87 | F | 1/1/1965 | 3/25/1991 | 11/20/2012 |  | 3/24/1994 | 5/31/2010 | 1 |
| 90 | F | 1/1/1983 | 3/25/1991 | 3/22/2013 |  | 3/24/1994 | 5/31/2010 | 1 |
| 92 | M | 7/1/2004 | 11/18/2004 | 3/22/2013 |  | 11/18/2004 | 5/31/2010 | 1 |
| 97 | F | 5/3/2002 | 5/29/2002 | 2/23/2013 |  | 5/3/2002 | 5/31/2010 | 1 |
| 98 | F | 1/1/2007 | 2/26/2007 | 11/20/2012 | 6/15/2012 | 1/1/2007 | 5/31/2010 | 1 |
| 101 | F | 1/1/1976 | 12/4/1994 | 4/15/2013 |  | 12/3/1997 | 5/31/2010 | 1 |
| 102 | F | 12/1/1994 | 12/4/1994 | 4/15/2013 |  | 12/1/1994 | 5/31/2010 | 1 |
| 103 | M | 3/16/2000 | 6/3/2000 | 6/3/2000 | 6/10/2000 | 6/3/2000 | 6/10/2000 | 0 |
| 104 | F | 4/19/2009 | 4/20/2009 | 4/15/2013 |  | 4/19/2009 | 5/31/2010 | 1 |
| 105 | F | 5/3/2002 | 5/21/2002 | 5/18/2011 | 10/5/2011 | 5/3/2002 | 5/31/2010 | 1 |
| 106 | F | 5/27/2007 | 7/25/2007 | 4/15/2013 |  | 5/27/2007 | 5/31/2010 | 1 |
| 111 | M | 1/1/1957 | 2/3/1992 | 11/3/2001 | 9/11/2002 | 2/2/1995 | 9/11/2002 | 0 |
| 123 | F | 1/1/1987 | 12/2/2005 | 1/19/2013 |  | 12/1/2008 | 5/31/2010 | 1 |
| 125 | F | 1/1/2002 | 12/2/2005 | 12/28/2012 |  | 12/2/2005 | 5/31/2010 | 1 |
| 126 | F | 4/6/2006 | 1/5/2007 | 1/19/2013 |  | 1/5/2007 | 5/31/2010 | 1 |
| 134 | F | 1/1/1958 | 2/3/1992 | 3/21/2013 |  | 2/2/1995 | 5/31/2010 | 1 |
| 139 | F | 1/1/1989 | 2/3/1992 | 11/26/2010 | 12/9/2011 | 2/2/1995 | 5/31/2010 | 1 |
| 140 | M | 4/17/1995 | 8/22/1995 | 12/2/2010 | 11/23/2011 | 8/22/1995 | 5/31/2010 | 1 |
| 141 | F | 3/24/2004 | 9/9/2004 | 11/26/2010 |  | 9/9/2004 | 5/31/2010 | 1 |
| 143 | F | 2/1/2000 | 1/5/2001 | 3/21/2013 |  | 1/5/2001 | 5/31/2010 | 1 |
| 144 | F | 5/8/2006 | 10/4/2006 | 3/21/2013 |  | 10/4/2006 | 5/31/2010 | 1 |
| 145 | F | 1/1/1968 | 1/29/1993 | 9/17/2012 |  | 1/29/1996 | 5/31/2010 | 1 |
| 146 | F | 1/1/1993 | 6/17/1994 | 11/22/2006 | 5/5/2008 | 6/17/1994 | 5/5/2008 | 0 |
| 147 | F | 1/6/1992 | 1/29/1993 | 12/18/2006 | 12/23/2007 | 1/29/1993 | 12/23/2007 | 1 |
| 150 | M | 1/19/1998 | 7/2/1998 | 5/25/2010 | 12/11/2010 | 7/2/1998 | 5/31/2010 | 1 |
| 152 | M | 11/24/2001 | 4/22/2002 | 9/30/2010 | 4/18/2011 | 4/22/2002 | 5/31/2010 | 1 |
| 153 | M | 8/1/2005 | 12/27/2005 | 9/17/2012 |  | 12/27/2005 | 5/31/2010 | 1 |
| 154 | M | 1/1/1968 | 6/24/2002 | 2/10/2012 |  | 6/23/2005 | 5/31/2010 | 1 |
| 160 | F | 1/1/1986 | 10/17/1991 | 2/24/2013 |  | 10/8/1995 | 5/31/2010 | 1 |
| 161 | F | 7/24/2002 | 10/17/2002 | 11/23/2012 |  | 10/17/2002 | 5/31/2010 | 1 |
| 162 | M | 3/10/2008 | 6/28/2008 | 11/12/2012 |  | 6/28/2008 | 5/31/2010 | 1 |
| 163 | M | 1/1/1944 | 11/1/1990 | 9/19/2004 | 8/5/2005 | 10/31/1993 | 8/5/2005 | 0 |
| 165 | F | 1/1/1955 | 1/31/1991 | 3/13/2013 |  | 1/30/1994 | 5/31/2010 | 1 |
| 168 | F | 1/1/1982 | 1/31/1991 | 2/24/2012 |  | 1/30/1994 | 5/31/2010 | 1 |
| 169 | M | 1/24/1992 | 7/30/1992 | 1/13/2003 | 8/11/2003 | 7/30/1992 | 8/11/2003 | 1 |
| 170 | F | 7/30/1999 | 12/17/2000 | 8/5/2009 | 2/24/2011 | 12/17/2000 | 5/31/2010 | 1 |
| 174 | F | 8/31/1996 | 10/7/1996 | 5/14/2011 | 1/11/2012 | 8/31/1996 | 5/31/2010 | 1 |
| 175 | M | 6/30/2001 | 7/17/2001 | 2/21/2012 |  | 6/30/2001 | 5/31/2010 | 1 |
| 176 | F | 5/30/2006 | 6/11/2006 | 3/13/2013 |  | 5/30/2006 | 5/31/2010 | 1 |
| 177 | M | 1/1/1941 | 1/15/1991 | 12/28/1994 | 9/16/1995 | 1/14/1994 | 9/16/1995 | 0 |
| 178 | M | 1/1/1965 | 11/11/1990 | 2/3/1994 | 4/30/1994 | 11/10/1993 | 4/30/1994 | 0 |
| 183 | F | 1/1/1962 | 3/8/2006 | 5/24/2012 |  | 3/7/2009 | 5/31/2010 | 1 |
| 186 | F | 1/1/1992 | 3/8/2006 | 1/29/2013 |  | 3/8/2006 | 5/31/2010 | 1 |
| 187 | F | 1/1/1997 | 3/8/2006 | 5/24/2012 |  | 3/8/2006 | 5/31/2010 | 1 |
| 188 | M | 5/23/2007 | 8/18/2007 | 1/29/2013 |  | 8/18/2007 | 5/31/2010 | 1 |
| 189 | F | 1/1/2003 | 2/25/2007 | 5/24/2012 |  | 2/25/2007 | 5/31/2010 | 1 |
| 190 | F | 5/7/2007 | 6/29/2007 | 5/24/2012 |  | 5/7/2007 | 5/31/2010 | 1 |
| 191 | F | 1/1/1977 | 9/1/1992 | 10/9/1996 | 2/4/1997 | 9/1/1995 | 2/4/1997 | 0 |
| 192 | M | 3/1/1992 | 9/1/1992 | 10/6/1996 | 2/27/1997 | 9/1/1992 | 2/27/1997 | 0 |
| 193 | M | 4/5/1995 | 4/13/1995 | 12/26/2011 | 5/12/2012 | 4/5/1995 | 5/31/2010 | 1 |
| 196 | F | 1/1/1982 | 1/3/1991 | 9/20/2007 | 1/4/2008 | 1/2/1994 | 1/4/2008 | 0 |
| 197 | F | 10/1/1999 | 11/1/2000 | 5/22/2012 | 10/25/2012 | 11/1/2000 | 5/31/2010 | 1 |
| 201 | F | 1/1/1981 | 4/29/1993 | 10/8/2007 | 3/15/2009 | 4/28/1996 | 3/15/2009 | 0 |
| 202 | M | 3/14/1999 | 10/24/1999 | 10/8/2007 | 5/31/2010 | 10/24/1999 | 5/31/2010 | 1 |
| 204 | F | 1/1/1968 | 4/28/1995 | 4/25/2008 | 8/31/2008 | 4/27/1998 | 8/31/2008 | 0 |
| 205 | F | 1/1/1970 | 8/23/1995 | 6/26/2001 | 4/20/2002 | 8/22/1998 | 4/20/2002 | 0 |
| 206 | M | 1/1/1965 | 8/23/1995 | 6/26/2001 | 4/20/2002 | 8/22/1998 | 4/20/2002 | 1 |
| 207 | F | 1/1/1983 | 6/6/1995 | 7/23/2008 | 1/5/2009 | 4/27/1998 | 1/5/2009 | 0 |
| 208 | M | 4/1/1991 | 4/28/1995 | 6/3/1999 | 10/1/1999 | 4/28/1995 | 10/1/1999 | 1 |
| 209 | F | 12/18/2000 | 4/29/2001 | 7/23/2008 | 2/6/2009 | 4/29/2001 | 2/6/2009 | 1 |
| 212 | F | 12/11/1997 | 4/17/1998 | 3/30/2008 | 9/4/2008 | 4/17/1998 | 9/4/2008 | 0 |
| 213 | M | 4/1/2003 | 4/15/2003 | 4/10/2008 | 9/19/2008 | 4/1/2003 | 9/19/2008 | 1 |
| 214 | M | 4/30/2007 | 3/30/2008 | 4/25/2008 | 5/21/2008 | 3/30/2008 | 5/21/2008 | 0 |
| 232 | F | 3/4/1936 | 3/25/1991 | 3/4/1996 | 7/4/1996 | 3/24/1994 | 7/4/1996 | 0 |
| 236 | F | 1/1/1966 | 1/28/1992 | 1/28/2013 |  | 1/27/1995 | 5/31/2010 | 1 |
| 239 | F | 6/1/1991 | 1/28/1992 | 1/9/2013 |  | 1/28/1992 | 5/31/2010 | 1 |
| 240 | F | 4/14/2007 | 7/2/2007 | 2/7/2012 |  | 7/2/2007 | 5/31/2010 | 1 |
| 243 | M | 9/20/1999 | 1/3/2000 | 1/4/2013 |  | 1/3/2000 | 5/31/2010 | 1 |
| 244 | F | 2/1/2008 | 6/17/2008 | 1/28/2013 |  | 6/17/2008 | 5/31/2010 | 1 |
| 248 | F | 1/1/1979 | 1/31/1992 | 12/28/2012 |  | 1/30/1995 | 5/31/2010 | 1 |
| 249 | M | 11/1/2001 | 12/24/2001 | 12/28/2012 |  | 11/1/2001 | 5/31/2010 | 1 |
| 250 | F | 4/5/2008 | 5/30/2008 | 12/28/2012 |  | 4/5/2008 | 5/31/2010 | 1 |
| 251 | M | 1/1/1970 | 10/4/1992 | 3/19/2013 |  | 10/4/1995 | 5/31/2010 | 1 |
| 257 | M | 1/1/1956 | 6/13/1991 | 7/12/1994 | 1/12/1995 | 6/12/1994 | 1/12/1995 | 0 |
| 264 | F | 1/1/1964 | 3/21/1991 | 1/9/2013 |  | 3/20/1994 | 5/31/2010 | 1 |
| 266 | F | 1/1/1975 | 3/21/1991 | 3/22/2013 |  | 3/20/1994 | 5/31/2010 | 1 |
| 269 | F | 1/1/1987 | 3/21/1991 | 2/15/2011 | 5/18/2011 | 3/20/1994 | 5/31/2010 | 1 |
| 271 | F | 6/13/2003 | 8/7/2003 | 5/28/2010 | 10/5/2010 | 6/13/2003 | 5/31/2010 | 1 |
| 274 | F | 5/14/2007 | 7/2/2007 | 5/28/2010 | 9/11/2010 | 5/14/2007 | 5/31/2010 | 0 |
| 276 | M | 12/21/1995 | 2/23/1996 | 1/19/2013 |  | 2/23/1996 | 5/31/2010 | 1 |
| 277 | M | 2/7/2001 | 3/12/2001 | 4/15/2013 |  | 2/7/2001 | 5/31/2010 | 1 |
| 278 | F | 1/1/2005 | 9/22/2005 | 1/29/2013 |  | 9/22/2005 | 5/31/2010 | 1 |
| 279 | F | 10/26/2007 | 12/5/2007 | 3/22/2013 |  | 10/26/2007 | 5/31/2010 | 1 |
| 281 | F | 1/1/1988 | 3/21/1991 | 1/29/2013 |  | 3/20/1994 | 5/31/2010 | 1 |
| 284 | F | 2/28/1994 | 3/11/1994 | 2/10/2012 | 6/21/2012 | 2/28/1994 | 5/31/2010 | 1 |
| 285 | M | 10/1/1998 | 1/28/1999 | 2/9/2012 | 7/12/2012 | 1/28/1999 | 5/31/2010 | 1 |
| 286 | F | 8/1/2003 | 8/7/2003 | 1/9/2013 |  | 8/1/2003 | 5/31/2010 | 1 |
| 310 | M | 1/1/1952 | 7/21/1992 | 10/12/2004 | 10/13/2005 | 7/21/1995 | 10/13/2005 | 0 |
| 311 | F | 1/1/1968 | 4/19/1992 | 3/15/2013 |  | 4/19/1995 | 5/31/2010 | 1 |
| 313 | F | 1/1/1984 | 9/27/1992 | 1/19/2013 |  | 4/19/1995 | 5/31/2010 | 1 |
| 314 | F | 1/1/1990 | 4/19/1992 | 3/21/2013 |  | 4/19/1995 | 5/31/2010 | 1 |
| 316 | F | 8/1/2005 | 10/19/2005 | 10/19/2012 |  | 10/19/2005 | 5/31/2010 | 1 |
| 319 | F | 6/1/2006 | 10/3/2006 | 3/18/2013 |  | 10/3/2006 | 5/31/2010 | 1 |
| 321 | F | 4/25/2002 | 6/10/2002 | 1/19/2013 |  | 4/25/2002 | 5/31/2010 | 1 |
| 322 | M | 6/15/1994 | 8/5/1994 | 6/29/2006 | 10/23/2006 | 6/15/1994 | 6/29/2006 | 1 |
| 323 | M | 6/1/1998 | 6/1/1998 | 2/16/2012 | 7/13/2012 | 6/1/1998 | 5/31/2010 | 1 |
| 324 | M | 11/11/2002 | 12/23/2002 | 3/28/2012 |  | 11/11/2002 | 5/31/2010 | 1 |
| 340 | F | 1/1/1972 | 8/24/1992 | 10/21/2007 | 4/2/2008 | 8/24/1995 | 4/2/2008 | 0 |
| 342 | M | 7/1/1994 | 7/13/1996 | 10/21/2007 | 4/10/2008 | 7/13/1996 | 4/10/2008 | 0 |
| 343 | M | 5/20/2002 | 6/1/2002 | 10/21/2007 | 5/10/2008 | 5/20/2002 | 5/10/2008 | 1 |
| 344 | F | 6/1/2006 | 7/18/2006 | 10/21/2007 | 3/5/2008 | 6/1/2006 | 3/5/2008 | 0 |
| 347 | F | 1/1/1953 | 2/11/1991 | 4/3/2009 | 11/7/2009 | 2/10/1994 | 11/7/2009 | 0 |
| 348 | F | 1/1/1980 | 1/22/1993 | 1/28/2013 |  | 2/10/1994 | 5/31/2010 | 1 |
| 349 | M | 6/30/1999 | 11/9/1999 | 3/23/2013 |  | 11/9/1999 | 5/31/2010 | 1 |
| 350 | M | 8/1/2005 | 10/1/2005 | 1/28/2013 |  | 10/1/2005 | 5/31/2010 | 1 |
| 351 | F | 1/1/1984 | 1/22/1993 | 3/21/2013 |  | 2/10/1994 | 5/31/2010 | 1 |
| 352 | F | 10/1/2008 | 11/11/2008 | 3/21/2013 |  | 10/1/2008 | 5/31/2010 | 1 |
| 353 | F | 1/1/1988 | 2/11/1991 | 11/11/2011 | 6/4/2012 | 2/10/1994 | 5/31/2010 | 1 |
| 355 | M | 11/7/1997 | 12/15/1997 | 12/10/2010 | 8/15/2011 | 11/7/1997 | 5/31/2010 | 1 |
| 357 | F | 1/1/1990 | 8/21/2003 | 11/14/2012 |  | 8/20/2006 | 5/31/2010 | 1 |
| 358 | F | 1/1/1977 | 8/17/2005 | 11/11/2011 | 4/9/2012 | 8/16/2008 | 5/31/2010 | 1 |
| 359 | F | 1/1/2002 | 8/17/2005 | 11/11/2011 | 4/15/2012 | 8/17/2005 | 5/31/2010 | 1 |
| 360 | M | 2/14/2006 | 6/25/2006 | 11/11/2011 | 4/15/2012 | 6/25/2006 | 5/31/2010 | 1 |
| 361 | F | 1/1/1996 | 8/21/2003 | 11/14/2012 |  | 8/21/2003 | 5/31/2010 | 1 |
| 362 | M | 8/1/2001 | 8/21/2003 | 11/14/2012 |  | 8/21/2003 | 5/31/2010 | 1 |
| 363 | M | 6/10/2007 | 8/15/2007 | 11/14/2012 |  | 8/15/2007 | 5/31/2010 | 1 |
| 372 | F | 1/1/1971 | 9/25/1991 | 2/14/2008 | 7/13/2008 | 9/24/1994 | 7/13/2008 | 0 |
| 373 | F | 1/1/1967 | 9/25/1991 | 3/23/2013 |  | 9/24/1994 | 5/31/2010 | 1 |
| 374 | F | 5/6/1995 | 8/11/1995 | 2/23/2013 |  | 8/11/1995 | 5/31/2010 | 1 |
| 375 | M | 1/10/2000 | 1/12/2000 | 10/13/2011 |  | 1/10/2000 | 5/31/2010 | 1 |
| 376 | F | 10/7/2006 | 7/6/2007 | 3/23/2013 |  | 7/6/2007 | 5/31/2010 | 1 |
| 377 | F | 5/1/1992 | 1/6/1993 | 12/5/2010 | 6/5/2011 | 1/6/1993 | 5/31/2010 | 1 |
| 378 | M | 7/1/2006 | 12/20/2006 | 12/5/2010 | 10/15/2011 | 12/20/2006 | 5/31/2010 | 1 |
| 379 | F | 6/22/1997 | 11/28/1997 | 3/23/2013 |  | 11/28/1997 | 5/31/2010 | 1 |
| 380 | M | 7/4/2001 | 12/8/2001 | 9/27/2012 |  | 12/8/2001 | 5/31/2010 | 1 |
| 381 | F | 3/1/2005 | 7/24/2005 | 2/14/2008 | 6/5/2008 | 7/24/2005 | 6/5/2008 | 0 |
| 405 | M | 1/1/1969 | 10/30/1991 | 1/27/2013 |  | 10/29/1994 | 5/31/2010 | 1 |
| 419 | M | 1/1/1957 | 1/26/1992 | 12/30/1997 | 11/1/1998 | 1/25/1995 | 11/1/1998 | 0 |
| 435 | F | 1/1/1985 | 1/5/1991 | 1/18/2013 |  | 1/4/1994 | 5/31/2010 | 1 |
| 436 | M | 7/26/1999 | 1/14/2000 | 1/8/2011 | 7/1/2011 | 1/14/2000 | 5/31/2010 | 1 |
| 437 | F | 5/4/2006 | 8/12/2006 | 1/18/2013 |  | 8/12/2006 | 5/31/2010 | 1 |
| 451 | M | 1/1/1984 | 8/20/2005 | 11/14/2011 |  | 8/19/2008 | 5/31/2010 | 1 |
| 459 | F | 1/1/1968 | 10/26/2003 | 1/9/2013 |  | 10/25/2006 | 5/31/2010 | 1 |
| 460 | M | 10/1/2001 | 10/26/2003 | 7/2/2009 | 3/31/2010 | 10/26/2003 | 3/31/2010 | 1 |
| 461 | M | 3/29/2006 | 6/7/2006 | 1/9/2013 |  | 6/7/2006 | 5/31/2010 | 1 |
| 463 | F | 1/1/1974 | 8/1/1993 | 11/4/2012 |  | 7/31/1996 | 5/31/2010 | 1 |
| 465 | M | 1/1/1987 | 8/1/1993 | 7/30/1998 | 1/8/1999 | 7/31/1996 | 1/8/1999 | 0 |
| 466 | F | 1/1/1990 | 8/1/1993 | 1/19/2006 | 7/4/2006 | 7/31/1996 | 1/19/2006 | 1 |
| 467 | M | 1/9/2005 | 12/27/2005 | 1/19/2006 | 3/6/2006 | 12/27/2005 | 3/6/2006 | 0 |
| 469 | F | 1/31/1997 | 5/28/1997 | 11/4/2012 |  | 5/28/1997 | 5/31/2010 | 1 |
| 470 | F | 5/5/2001 | 7/21/2001 | 11/4/2012 |  | 7/21/2001 | 5/31/2010 | 1 |
| 471 | M | 7/20/2005 | 12/27/2005 | 12/22/2011 |  | 12/27/2005 | 5/31/2010 | 1 |
| 474 | F | 1/1/1951 | 1/7/1991 | 12/6/1997 | 2/13/2010 | 1/6/1994 | 2/13/2010 | 0 |
| 475 | M | 1/1/1981 | 1/7/1991 | 1/9/2009 | 7/26/2009 | 1/6/1994 | 7/26/2009 | 0 |
| 476 | F | 1/1/1963 | 11/12/1990 | 11/23/2012 |  | 11/11/1993 | 5/31/2010 | 1 |
| 477 | M | 1/1/1985 | 11/12/1990 | 12/23/2006 | 6/11/2007 | 11/11/1993 | 12/23/2006 | 1 |
| 478 | M | 7/1/1995 | 7/15/1996 | 11/29/2010 | 3/19/2011 | 7/15/1996 | 5/31/2010 | 1 |
| 479 | F | 5/9/2004 | 8/20/2004 | 11/23/2012 |  | 8/20/2004 | 5/31/2010 | 1 |
| 485 | M | 1/1/1942 | 4/20/1992 | 4/16/2000 | 1/15/2001 | 4/20/1995 | 1/15/2001 | 0 |
| 504 | F | 1/1/1963 | 8/29/1992 | 4/16/2002 | 10/19/2002 | 8/29/1995 | 10/19/2002 | 0 |
| 505 | F | 1/1/1987 | 8/29/1992 | 7/19/1999 | 1/3/2000 | 8/29/1995 | 1/3/2000 | 1 |
| 506 | F | 1/1/1992 | 8/29/1992 | 3/16/2001 | 9/12/2001 | 8/29/1992 | 9/12/2001 | 1 |
| 507 | M | 6/15/1999 | 7/16/1999 | 3/16/2001 | 8/31/2001 | 6/15/1999 | 8/31/2001 | 0 |
| 535 | F | 1/1/1957 | 11/23/1992 | 4/23/1996 | 10/26/1996 | 11/23/1995 | 10/26/1996 | 0 |
| 536 | M | 1/1/1984 | 12/6/1992 | 1/13/1997 | 9/18/1997 | 11/23/1995 | 1/13/1997 | 1 |
| 537 | F | 11/1/1991 | 12/23/1993 | 4/23/1996 | 9/6/1996 | 12/23/1993 | 9/6/1996 | 0 |
| 538 | M | 1/1/1962 | 1/26/1994 | 2/14/2001 | 9/24/2001 | 1/25/1997 | 9/24/2001 | 0 |
| 543 | M | 1/1/1956 | 10/4/1991 | 8/28/1999 | 4/2/2000 | 10/3/1994 | 4/2/2000 | 0 |
| 565 | F | 1/1/1979 | 10/13/1991 | 3/22/2013 |  | 10/12/1994 | 5/31/2010 | 1 |
| 566 | M | 5/28/2003 | 8/14/2003 | 3/22/2013 |  | 8/14/2003 | 5/31/2010 | 1 |
| 568 | F | 1/1/1966 | 9/1/1991 | 5/26/2012 |  | 8/31/1994 | 5/31/2010 | 1 |
| 569 | F | 1/1/1990 | 9/1/1991 | 5/26/2012 |  | 8/31/1994 | 5/31/2010 | 1 |
| 570 | M | 12/21/1999 | 5/12/2000 | 5/24/2011 | 3/25/2012 | 5/12/2000 | 5/31/2010 | 1 |
| 571 | F | 6/1/2007 | 4/9/2008 | 5/26/2012 |  | 4/9/2008 | 5/31/2010 | 1 |
| 573 | M | 6/1/2007 | 4/4/2008 | 5/26/2012 |  | 4/4/2008 | 5/31/2010 | 1 |
| 578 | F | 1/1/1971 | 11/7/1990 | 12/20/2011 | 7/17/2012 | 11/6/1993 | 5/31/2010 | 1 |
| 579 | M | 1/1/1988 | 11/7/1990 | 12/21/2011 | 6/18/2012 | 11/6/1993 | 5/31/2010 | 1 |
| 580 | M | 11/1/1991 | 11/4/1991 | 7/6/2006 | 2/15/2007 | 11/1/1991 | 2/15/2007 | 1 |
| 581 | F | 7/4/1997 | 9/30/1997 | 12/20/2011 |  | 9/30/1997 | 5/31/2010 | 1 |
| 582 | M | 7/1/2003 | 11/19/2003 | 2/9/2010 | 8/30/2010 | 11/19/2003 | 5/31/2010 | 1 |
| 583 | F | 7/1/2007 | 7/9/2007 | 12/20/2011 | 7/19/2012 | 7/1/2007 | 5/31/2010 | 1 |
| 584 | F | 1/1/1980 | 5/12/1990 | 8/28/1999 | 5/18/2000 | 5/11/1993 | 5/18/2000 | 0 |
| 585 | F | 1/1/1982 | 5/12/1990 | 8/28/1999 | 5/18/2000 | 5/11/1993 | 5/18/2000 | 1 |
| 586 | F | 1/1/1986 | 5/12/1990 | 8/28/1999 | 5/18/2000 | 5/11/1993 | 5/18/2000 | 1 |
| 587 | F | 3/26/1991 | 8/19/1991 | 8/28/1999 | 5/24/2000 | 8/19/1991 | 5/24/2000 | 1 |
| 588 | M | 3/2/1998 | 8/28/1998 | 8/28/1999 | 8/20/2000 | 8/28/1998 | 8/20/2000 | 0 |
| 589 | F | 1/1/1987 | 8/17/1994 | 9/23/2002 | 5/25/2003 | 8/16/1997 | 5/25/2003 | 0 |
| 590 | F | 1/1/1952 | 3/25/1991 | 1/4/2013 |  | 3/24/1994 | 5/31/2010 | 1 |
| 591 | F | 1/1/1976 | 3/25/1991 | 1/4/2013 |  | 3/24/1994 | 5/31/2010 | 1 |
| 592 | M | 1/1/1990 | 3/25/1991 | 1/4/2013 |  | 3/24/1994 | 5/31/2010 | 1 |
| 593 | F | 9/6/2006 | 12/28/2006 | 1/28/2012 |  | 12/28/2006 | 5/31/2010 | 1 |
| 594 | F | 10/12/2007 | 4/3/2008 | 1/4/2013 |  | 4/3/2008 | 5/31/2010 | 1 |
| 596 | M | 2/1/2000 | 3/28/2000 | 2/12/2010 | 11/5/2010 | 2/1/2000 | 5/31/2010 | 1 |
| 598 | M | 1/1/1945 | 10/20/1990 | 2/9/2001 | 3/24/2002 | 10/19/1993 | 3/24/2002 | 0 |
| 599 | M | 1/1/1971 | 10/4/1991 | 3/7/2012 |  | 10/3/1994 | 5/31/2010 | 1 |
| 611 | F | 1/1/1968 | 9/19/1992 | 5/3/2012 |  | 9/19/1995 | 5/31/2010 | 1 |
| 612 | M | 1/1/1987 | 9/19/1992 | 3/15/2000 | 9/21/2000 | 9/19/1995 | 9/21/2000 | 1 |
| 613 | F | 11/8/1993 | 5/7/1994 | 11/30/2012 |  | 5/7/1994 | 5/31/2010 | 1 |
| 614 | F | 11/22/1996 | 1/11/1997 | 12/4/2010 | 8/23/2011 | 11/22/1996 | 5/31/2010 | 1 |
| 615 | M | 9/28/1999 | 3/11/2000 | 5/10/2010 | 1/17/2011 | 3/11/2000 | 5/31/2010 | 1 |
| 616 | F | 7/1/2004 | 9/1/2004 | 5/10/2010 | 3/22/2011 | 9/1/2004 | 5/31/2010 | 1 |
| 619 | F | 1/1/1973 | 9/26/1991 | 1/4/2013 |  | 9/25/1994 | 5/31/2010 | 1 |
| 620 | F | 1/1/1985 | 9/26/1991 | 2/23/1999 | 6/17/1999 | 9/25/1994 | 2/23/1999 | 1 |
| 621 | M | 5/1/1997 | 8/9/1997 | 11/3/2013 |  | 8/9/1997 | 5/31/2010 | 1 |
| 622 | F | 4/1/2003 | 8/7/2003 | 1/4/2013 |  | 8/7/2003 | 5/31/2010 | 1 |
| 637 | M | 1/1/1946 | 9/18/1991 | 1/21/2013 |  | 9/17/1994 | 5/31/2010 | 1 |
| 638 | F | 1/1/1968 | 6/18/1991 | 1/6/2013 |  | 6/17/1994 | 5/31/2010 | 1 |
| 640 | F | 6/1/1990 | 6/18/1991 | 11/1/2012 |  | 6/18/1991 | 5/31/2010 | 1 |
| 642 | F | 6/21/1994 | 11/14/1994 | 10/27/2007 | 6/17/2008 | 11/14/1994 | 6/17/2008 | 0 |
| 643 | M | 10/30/1998 | 12/29/1999 | 11/13/2012 |  | 12/29/1999 | 5/31/2010 | 1 |
| 644 | F | 10/26/2008 | 4/28/2009 | 1/6/2013 |  | 4/28/2009 | 5/31/2010 | 1 |
| 645 | F | 1/1/1965 | 5/1/1990 | 12/30/1993 | 9/24/1994 | 4/30/1993 | 9/24/1994 | 0 |
| 647 | M | 1/1/1984 | 12/9/2004 | 5/8/2011 | 1/31/2012 | 12/9/2007 | 5/31/2010 | 1 |
| 654 | M | 1/1/1980 | 8/3/2005 | 3/13/2013 |  | 8/2/2008 | 5/31/2010 | 1 |
| 655 | F | 1/1/1960 | 11/12/1991 | 11/9/2009 | 6/27/2010 | 11/11/1994 | 5/31/2010 | 1 |
| 656 | F | 1/1/1984 | 1/31/1994 | 1/28/2013 |  | 11/11/1994 | 5/31/2010 | 1 |
| 657 | M | 1/1/1989 | 11/12/1991 | 10/21/2007 | 1/21/2009 | 11/11/1994 | 1/21/2009 | 0 |
| 659 | F | 6/9/1999 | 10/31/1999 | 11/13/2012 |  | 10/31/1999 | 5/31/2010 | 1 |
| 660 | F | 8/1/2003 | 11/6/2003 | 1/28/2013 |  | 11/6/2003 | 5/31/2010 | 1 |
| 663 | F | 5/17/1992 | 11/30/1992 | 11/9/2009 | 6/27/2010 | 11/30/1992 | 5/31/2010 | 1 |
| 665 | M | 6/9/1999 | 10/31/1999 | 11/9/2009 | 7/9/2010 | 10/31/1999 | 5/31/2010 | 1 |
| 669 | F | 1/1/1960 | 2/22/1992 | 3/19/2013 |  | 2/21/1995 | 5/31/2010 | 1 |
| 670 | F | 1/1/1964 | 2/22/1992 | 12/3/2012 |  | 2/21/1995 | 5/31/2010 | 1 |
| 671 | M | 8/1/2003 | 2/5/2004 | 10/5/2012 |  | 2/5/2004 | 5/31/2010 | 1 |
| 673 | M | 6/16/1995 | 9/12/1995 | 1/28/2013 |  | 9/12/1995 | 5/31/2010 | 1 |
| 674 | M | 2/1/2001 | 3/27/2002 | 2/26/2013 |  | 3/27/2002 | 5/31/2010 | 1 |
| 675 | M | 6/28/2007 | 9/25/2007 | 3/19/2013 |  | 9/25/2007 | 5/31/2010 | 1 |
| 678 | M | 1/1/1941 | 3/26/1991 | 3/12/2001 | 9/12/2001 | 3/25/1994 | 9/12/2001 | 0 |
| 682 | M | 1/1/1960 | 3/24/1995 | 1/15/2007 | 7/14/2007 | 3/23/1998 | 7/14/2007 | 0 |
| 683 | F | 1/1/1966 | 3/11/1995 | 7/19/2000 | 10/15/2000 | 3/10/1998 | 10/15/2000 | 0 |
| 684 | M | 1/1/1941 | 10/18/1990 | 4/13/2001 | 8/25/2001 | 10/17/1993 | 8/25/2001 | 0 |
| 686 | F | 1/1/1983 | 3/11/1995 | 1/4/2000 | 5/11/2000 | 3/10/1998 | 5/11/2000 | 1 |
| 687 | M | 1/1/1992 | 3/11/1995 | 6/20/1999 | 10/20/1999 | 3/11/1995 | 10/20/1999 | 1 |
| 688 | F | 1/1/1997 | 1/12/1997 | 7/19/2000 | 10/19/2000 | 1/1/1997 | 10/19/2000 | 0 |
| 690 | M | 1/1/1946 | 2/3/1991 | 3/1/1994 | 11/28/1994 | 2/2/1994 | 11/28/1994 | 0 |
| 699 | M | 1/1/1975 | 3/16/1992 | 1/28/2013 |  | 3/16/1995 | 5/31/2010 | 1 |
| 700 | F | 1/1/1956 | 2/1/1991 | 1/5/2000 | 8/22/2000 | 1/31/1994 | 8/22/2000 | 0 |
| 701 | F | 8/1/1988 | 2/1/1991 | 2/14/1999 | 11/7/1999 | 1/31/1994 | 11/7/1999 | 1 |
| 702 | M | 1/2/1997 | 4/4/1997 | 1/5/2000 | 8/22/2000 | 4/4/1997 | 8/22/2000 | 0 |
| 713 | F | 1/1/1970 | 2/11/1993 | 3/13/2013 |  | 2/11/1996 | 5/31/2010 | 1 |
| 714 | F | 1/1/1983 | 2/11/1993 | 3/31/2004 | 6/19/2006 | 2/11/1996 | 6/19/2006 | 1 |
| 715 | M | 1/1/1990 | 2/11/1993 | 4/20/2004 | 6/24/2005 | 2/11/1996 | 6/24/2005 | 1 |
| 716 | M | 1/15/1995 | 3/9/1995 | 2/22/2012 |  | 1/15/1995 | 5/31/2010 | 1 |
| 717 | F | 1/5/2002 | 3/26/2002 | 3/13/2013 |  | 3/26/2002 | 5/31/2010 | 1 |
| 718 | F | 7/1/2008 | 10/10/2008 | 3/13/2013 |  | 10/10/2008 | 5/31/2010 | 1 |
| 736 | F | 1/1/1972 | 9/10/1992 | 3/12/2013 |  | 9/10/1995 | 5/31/2010 | 1 |
| 737 | M | 1/1/1987 | 2/9/1993 | 3/10/2000 | 1/22/2001 | 9/10/1995 | 1/22/2001 | 1 |
| 738 | M | 1/1/1990 | 9/10/1992 | 2/25/2012 |  | 9/10/1995 | 5/31/2010 | 1 |
| 739 | F | 6/30/1998 | 9/12/1998 | 3/12/2013 |  | 9/12/1998 | 5/31/2010 | 1 |
| 740 | F | 1/1/2005 | 1/28/2005 | 3/12/2013 |  | 1/1/2005 | 5/31/2010 | 1 |
| 741 | F | 3/13/2009 | 3/14/2009 | 3/12/2013 |  | 3/13/2009 | 5/31/2010 | 1 |
| 753 | M | 1/1/1982 | 2/24/2003 | 11/3/2013 |  | 2/23/2006 | 5/31/2010 | 1 |
| 759 | F | 1/1/1941 | 11/11/1992 | 4/25/2004 | 11/3/2004 | 11/11/1995 | 11/3/2004 | 0 |
| 761 | F | 1/1/1989 | 11/11/1992 | 4/30/2003 | 8/18/2004 | 11/11/1995 | 8/18/2004 | 1 |
| 762 | F | 1/1/1990 | 11/11/1992 | 10/15/2002 | 7/10/2003 | 11/11/1995 | 7/10/2003 | 1 |
| 763 | F | 1/1/1947 | 9/28/1992 | 9/6/2003 | 5/3/2004 | 9/28/1995 | 5/3/2004 | 0 |
| 764 | F | 1/1/1986 | 7/5/1991 | 7/1/2001 | 9/30/2002 | 9/28/1995 | 7/1/2001 | 1 |
| 765 | F | 1/1/1992 | 9/28/1992 | 9/6/2003 | 5/19/2004 | 9/28/1992 | 5/19/2004 | 1 |
| 770 | F | 1/1/1955 | 11/4/1990 | 12/28/2012 |  | 11/3/1993 | 5/31/2010 | 1 |
| 773 | F | 1/1/1970 | 11/4/1990 | 2/23/2013 |  | 11/3/1993 | 5/31/2010 | 1 |
| 775 | F | 5/5/2005 | 7/26/2005 | 2/23/2013 |  | 7/26/2005 | 5/31/2010 | 1 |
| 776 | M | 4/6/2009 | 5/11/2009 | 2/23/2013 |  | 4/6/2009 | 5/31/2010 | 1 |
| 777 | M | 1/1/1986 | 11/4/1990 | 12/28/2012 |  | 11/3/1993 | 5/31/2010 | 1 |
| 778 | F | 4/11/1995 | 4/18/1995 | 3/24/2010 | 8/13/2010 | 4/11/1995 | 5/31/2010 | 1 |
| 779 | M | 8/21/2000 | 10/28/2000 | 10/5/2012 |  | 10/28/2000 | 5/31/2010 | 1 |
| 785 | M | 3/24/1992 | 3/24/1992 | 10/9/2012 |  | 3/24/1992 | 5/31/2010 | 1 |
| 787 | F | 10/1/1996 | 12/22/1996 | 10/8/2011 | 2/11/2012 | 12/22/1996 | 5/31/2010 | 1 |
| 788 | M | 7/24/2001 | 12/9/2001 | 12/28/2012 |  | 12/9/2001 | 5/31/2010 | 1 |
| 799 | F | 1/1/1983 | 4/29/1997 | 3/21/2013 |  | 4/28/2000 | 5/31/2010 | 1 |
| 800 | M | 10/14/2001 | 4/8/2002 | 2/21/2012 | 8/17/2012 | 4/8/2002 | 5/31/2010 | 1 |
| 806 | M | 1/1/1951 | 1/13/1991 | 2/11/2004 | 8/7/2004 | 1/12/1994 | 8/7/2004 | 0 |
| 808 | F | 1/1/1941 | 6/19/1991 | 5/3/1996 | 1/6/1997 | 6/18/1994 | 1/6/1997 | 0 |
| 809 | M | 1/1/1983 | 7/18/1992 | 4/21/1998 | 5/8/1999 | 6/18/1994 | 4/21/1998 | 1 |
| 810 | F | 6/1/1991 | 7/18/1992 | 8/7/1997 | 2/23/1998 | 7/18/1992 | 2/23/1998 | 0 |
| 812 | F | 3/10/1995 | 9/24/1995 | 5/3/1996 | 7/7/1997 | 9/24/1995 | 7/7/1997 | 0 |
| 814 | M | 1/1/1961 | 1/25/1991 | 4/30/1994 | 2/18/1995 | 1/24/1994 | 2/18/1995 | 0 |
| 816 | F | 1/1/1958 | 10/2/1992 | 3/22/2013 |  | 10/2/1995 | 5/31/2010 | 1 |
| 818 | F | 1/1/1986 | 10/2/1992 | 3/22/2013 |  | 10/2/1995 | 5/31/2010 | 1 |
| 819 | M | 1/1/1992 | 10/2/1992 | 11/5/2012 |  | 10/2/1992 | 5/31/2010 | 1 |
| 827 | M | 1/1/1994 | 3/8/1996 | 2/28/2012 |  | 3/8/1996 | 5/31/2010 | 1 |
| 842 | F | 1/1/1960 | 8/7/1991 | 2/13/2013 |  | 8/6/1994 | 5/31/2010 | 1 |
| 843 | F | 1/1/1974 | 8/7/1991 | 3/12/2013 |  | 8/6/1994 | 5/31/2010 | 1 |
| 844 | M | 3/1/1992 | 7/30/1992 | 7/6/2009 | 5/26/2010 | 7/30/1992 | 7/6/2009 | 1 |
| 850 | M | 1/16/1997 | 4/21/1997 | 9/13/2012 |  | 4/21/1997 | 5/31/2010 | 1 |
| 851 | M | 12/1/2001 | 3/15/2003 | 2/7/2013 |  | 3/15/2003 | 5/31/2010 | 1 |
| 852 | F | 11/1/2005 | 7/31/2006 | 2/13/2013 |  | 7/31/2006 | 5/31/2010 | 1 |
| 854 | M | 1/1/1970 | 4/12/2002 | 2/5/2013 |  | 4/11/2005 | 5/31/2010 | 1 |
| 855 | M | 1/1/1948 | 2/9/1993 | 11/4/2001 | 9/14/2002 | 2/9/1996 | 9/14/2002 | 0 |
| 856 | F | 1/1/1962 | 6/28/1991 | 11/21/2002 | 4/8/2003 | 6/27/1994 | 4/8/2003 | 0 |
| 857 | F | 1/1/1988 | 6/28/1991 | 5/24/1997 | 3/2/1998 | 6/27/1994 | 3/2/1998 | 1 |
| 858 | M | 10/1/1990 | 6/28/1991 | 6/15/2000 | 12/2/2000 | 6/28/1991 | 12/2/2000 | 1 |
| 859 | F | 1/5/1996 | 4/24/1996 | 11/21/2002 | 3/7/2003 | 4/24/1996 | 3/7/2003 | 1 |
| 866 | F | 1/1/1970 | 1/28/1991 | 2/4/2013 |  | 1/27/1994 | 5/31/2010 | 1 |
| 868 | F | 1/1/1985 | 1/28/1991 | 3/6/2013 |  | 1/27/1994 | 5/31/2010 | 1 |
| 869 | M | 4/27/1991 | 6/7/1991 | 5/18/2010 | 1/5/2011 | 4/27/1991 | 5/31/2010 | 1 |
| 879 | F | 10/9/1996 | 12/27/1996 | 2/1/2011 | 1/1/2012 | 12/27/1996 | 5/31/2010 | 1 |
| 880 | M | 9/28/2000 | 1/26/2001 | 1/3/2013 |  | 1/26/2001 | 5/31/2010 | 1 |
| 881 | M | 9/25/2005 | 1/11/2006 | 2/28/2013 |  | 1/11/2006 | 5/31/2010 | 1 |
| 887 | M | 1/1/1941 | 1/2/1991 | 2/12/2003 | 12/21/2003 | 1/1/1994 | 12/21/2003 | 0 |
| 888 | M | 1/1/1962 | 4/27/1992 | 8/25/1998 | 10/13/1999 | 4/27/1995 | 10/13/1999 | 0 |
| 897 | F | 1/1/1948 | 6/14/1996 | 5/8/2003 | 11/30/2003 | 6/14/1999 | 11/30/2003 | 0 |
| 898 | F | 1/1/1990 | 6/14/1996 | 2/9/2003 | 11/20/2003 | 6/14/1999 | 11/20/2003 | 1 |
| 899 | M | 5/18/1999 | 6/14/1999 | 5/8/2003 | 9/17/2003 | 5/18/1999 | 9/17/2003 | 0 |
| 900 | F | 1/1/1936 | 3/21/1991 | 9/19/1997 | 5/25/1998 | 3/20/1994 | 5/25/1998 | 0 |
| 901 | F | 1/1/1979 | 3/21/1991 | 6/16/1994 | 8/12/1996 | 3/20/1994 | 8/12/1996 | 1 |
| 902 | M | 3/1/1985 | 3/21/1991 | 9/19/1997 | 1/6/1999 | 3/20/1994 | 1/6/1999 | 1 |
| 923 | M | 1/1/1976 | 8/3/1992 | 12/3/2013 |  | 8/3/1995 | 5/31/2010 | 1 |
| 924 | F | 1/1/1980 | 8/13/2003 | 1/27/2013 |  | 8/12/2006 | 5/31/2010 | 1 |
| 925 | M | 1/1/1998 | 8/13/2003 | 1/27/2013 |  | 8/13/2003 | 5/31/2010 | 1 |
| 926 | F | 12/1/2003 | 5/31/2005 | 1/27/2013 |  | 5/31/2005 | 5/31/2010 | 1 |
| 929 | F | 1/1/1971 | 2/25/1991 | 3/28/2000 | 7/4/2000 | 2/24/1994 | 7/4/2000 | 0 |
| 930 | M | 1/1/1990 | 8/31/1991 | 4/13/2000 | 7/26/2000 | 2/24/1994 | 4/13/2000 | 1 |
| 931 | M | 10/24/1992 | 11/6/1992 | 2/6/2000 | 5/24/2000 | 10/24/1992 | 5/24/2000 | 1 |
| 932 | F | 11/8/1997 | 12/17/1997 | 3/28/2000 | 6/18/2000 | 11/8/1997 | 6/18/2000 | 0 |
| 937 | F | 1/1/1960 | 1/11/1993 | 1/13/1997 | 12/25/1997 | 1/11/1996 | 12/25/1997 | 0 |
| 939 | F | 1/1/1971 | 7/31/1992 | 7/27/2000 | 11/18/2000 | 7/31/1995 | 11/18/2000 | 0 |
| 940 | M | 1/1/1986 | 7/31/1992 | 6/27/1999 | 1/21/2000 | 7/31/1995 | 1/21/2000 | 1 |
| 941 | M | 1/1/1992 | 7/31/1992 | 6/30/2000 | 11/9/2000 | 7/31/1992 | 11/9/2000 | 1 |
| 942 | F | 7/24/1999 | 8/20/1999 | 7/27/2000 | 12/6/2000 | 7/24/1999 | 12/6/2000 | 0 |
| 943 | M | 1/1/1946 | 8/10/1991 | 3/27/1995 | 7/11/1995 | 8/9/1994 | 7/11/1995 | 0 |
| 947 | M | 1/1/1959 | 4/20/1994 | 4/8/1998 | 9/15/1998 | 4/19/1997 | 9/15/1998 | 0 |
| 951 | F | 1/1/1975 | 6/6/1991 | 12/3/2012 |  | 6/5/1994 | 5/31/2010 | 1 |
| 952 | F | 1/1/1991 | 6/6/1991 | 2/27/2013 |  | 6/6/1991 | 5/31/2010 | 1 |
| 953 | F | 5/15/1994 | 7/5/1994 | 11/17/2011 |  | 5/15/1994 | 5/31/2010 | 1 |
| 954 | F | 3/21/2007 | 7/19/2007 | 2/27/2013 |  | 7/19/2007 | 5/31/2010 | 1 |
| 957 | F | 12/1/1999 | 12/30/1999 | 12/3/2012 |  | 12/1/1999 | 5/31/2010 | 1 |
| 958 | M | 4/1/2004 | 8/8/2004 | 11/11/2012 |  | 8/8/2004 | 5/31/2010 | 1 |
| 959 | F | 11/1/2008 | 4/13/2009 | 12/3/2012 |  | 4/13/2009 | 5/31/2010 | 1 |
| 961 | F | 1/1/1989 | 5/26/2001 | 1/29/2013 |  | 5/25/2004 | 5/31/2010 | 1 |
| 962 | M | 5/1/1999 | 5/26/2001 | 2/15/2011 |  | 5/26/2001 | 5/31/2010 | 1 |
| 963 | M | 1/1/2002 | 5/24/2002 | 1/29/2013 |  | 5/24/2002 | 5/31/2010 | 1 |
| 965 | F | 1/1/1962 | 11/4/1991 | 1/28/2013 |  | 11/3/1994 | 5/31/2010 | 1 |
| 969 | M | 1/1/1979 | 8/29/1992 | 12/20/2002 | 8/5/2003 | 11/3/1994 | 12/20/2002 | 1 |
| 970 | M | 1/1/1988 | 11/4/1991 | 5/22/2002 | 1/31/2003 | 11/3/1994 | 1/31/2003 | 1 |
| 971 | F | 6/28/1995 | 9/18/1995 | 12/2/2005 | 9/18/2006 | 9/18/1995 | 9/18/2006 | 1 |
| 972 | M | 6/1/1998 | 7/11/1999 | 7/10/2007 | 4/1/2008 | 7/11/1999 | 4/1/2008 | 1 |
| 973 | F | 3/1/2003 | 8/7/2003 | 1/28/2013 |  | 8/7/2003 | 5/31/2010 | 1 |
| 974 | F | 2/1/2006 | 8/25/2006 | 1/28/2013 |  | 8/25/2006 | 5/31/2010 | 1 |
| 977 | M | 1/1/1960 | 10/19/1990 | 3/22/2013 |  | 10/18/1993 | 5/31/2010 | 1 |
| 981 | F | 1/1/1966 | 4/11/1992 | 4/27/2008 | 1/10/2009 | 4/11/1995 | 1/10/2009 | 0 |
| 983 | F | 4/1/1988 | 4/11/1992 | 9/17/1997 | 7/1/2009 | 4/11/1995 | 7/1/2009 | 1 |
| 984 | M | 1/1/1991 | 4/11/1992 | 4/22/2007 | 1/19/2008 | 4/11/1992 | 1/19/2008 | 1 |
| 985 | M | 4/3/1998 | 7/7/1998 | 4/27/2008 | 4/30/2008 | 7/7/1998 | 4/30/2008 | 1 |
| 986 | F | 7/1/2004 | 8/2/2005 | 4/27/2008 | 9/5/2009 | 8/2/2005 | 9/5/2009 | 0 |
| 987 | M | 1/1/1966 | 11/9/1991 | 5/13/2012 |  | 11/8/1994 | 5/31/2010 | 1 |
| 992 | F | 1/1/1961 | 7/8/1991 | 3/21/2013 |  | 7/7/1994 | 5/31/2010 | 1 |
| 993 | F | 1/1/1989 | 7/8/1991 | 5/18/2012 |  | 7/7/1994 | 5/31/2010 | 1 |
| 994 | M | 10/18/1998 | 3/23/1999 | 8/25/1999 | 12/5/1999 | 3/23/1999 | 12/5/1999 | 0 |
| 995 | F | 4/4/2008 | 4/10/2008 | 5/18/2012 |  | 4/4/2008 | 5/31/2010 | 1 |
| 997 | M | 9/1/2002 | 3/5/2003 | 5/10/2012 |  | 3/5/2003 | 5/31/2010 | 1 |
| 998 | F | 11/12/2008 | 4/15/2009 | 3/21/2013 |  | 4/15/2009 | 5/31/2010 | 1 |
| 1001 | M | 1/1/1961 | 1/19/1991 | 3/4/1996 | 9/6/1996 | 1/18/1994 | 9/6/1996 | 0 |
| 1002 | F | 1/1/1966 | 4/21/1997 | 10/10/2012 |  | 4/20/2000 | 5/31/2010 | 1 |
| 1003 | F | 1/1/1993 | 4/21/1997 | 2/25/2012 |  | 4/21/1997 | 5/31/2010 | 1 |
| 1004 | F | 12/26/1998 | 4/4/1999 | 10/10/2012 |  | 4/4/1999 | 5/31/2010 | 1 |
| 1005 | F | 4/28/2004 | 5/11/2004 | 10/10/2012 |  | 4/28/2004 | 5/31/2010 | 1 |
| 1009 | F | 1/1/1970 | 1/21/1991 | 11/1/2012 |  | 1/20/1994 | 5/31/2010 | 1 |
| 1010 | F | 1/1/1988 | 3/23/1992 | 10/25/2012 |  | 1/20/1994 | 5/31/2010 | 1 |
| 1011 | M | 1/10/1994 | 4/17/1995 | 10/16/2012 |  | 4/17/1995 | 5/31/2010 | 1 |
| 1012 | M | 9/1/1999 | 12/27/1999 | 10/25/2012 |  | 12/27/1999 | 5/31/2010 | 1 |
| 1013 | F | 4/3/2006 | 10/1/2006 | 11/1/2012 |  | 10/1/2006 | 5/31/2010 | 1 |
| 1040 | M | 1/1/1980 | 2/20/1992 | 2/7/1997 | 10/17/1997 | 2/19/1995 | 10/17/1997 | 0 |
| 1048 | M | 1/1/1966 | 8/9/1996 | 7/19/2000 | 3/12/2001 | 8/9/1999 | 3/12/2001 | 0 |
| 1049 | F | 1/1/1936 | 1/14/1992 | 4/13/1996 | 11/19/1996 | 1/13/1995 | 11/19/1996 | 0 |
| 1051 | F | 1/1/1972 | 3/24/1992 | 4/15/2013 |  | 3/24/1995 | 5/31/2010 | 1 |
| 1052 | F | 1/1/1988 | 3/30/2001 | 4/15/2013 |  | 3/29/2004 | 5/31/2010 | 1 |
| 1053 | F | 11/1/2002 | 1/24/2003 | 4/15/2013 |  | 1/24/2003 | 5/31/2010 | 1 |
| 1054 | M | 1/17/2000 | 4/11/2000 | 4/15/2013 |  | 4/11/2000 | 5/31/2010 | 1 |
| 1055 | F | 12/7/2006 | 4/19/2007 | 5/21/2012 |  | 4/19/2007 | 5/31/2010 | 1 |
| 1059 | M | 1/1/1967 | 4/5/1992 | 4/19/2003 | 4/7/2004 | 4/5/1995 | 4/7/2004 | 0 |
| 1066 | M | 10/1/1995 | 10/6/1995 | 1/5/2007 | 7/24/2007 | 10/1/1995 | 7/24/2007 | 0 |
| 1074 | F | 1/1/1969 | 2/1/1992 | 3/4/2013 |  | 1/31/1995 | 5/31/2010 | 1 |
| 1075 | F | 1/1/1973 | 1/20/1993 | 3/3/2013 |  | 1/31/1995 | 5/31/2010 | 1 |
| 1076 | F | 1/20/1995 | 3/8/1995 | 3/8/2013 |  | 1/20/1995 | 5/31/2010 | 1 |
| 1077 | F | 10/16/1999 | 2/18/2000 | 2/12/2013 |  | 2/18/2000 | 5/31/2010 | 1 |
| 1078 | F | 12/1/2003 | 2/4/2004 | 2/12/2013 |  | 2/4/2004 | 5/31/2010 | 1 |
| 1079 | M | 7/1/2007 | 7/16/2007 | 3/3/2013 |  | 7/1/2007 | 5/31/2010 | 1 |
| 1080 | M | 2/1/1986 | 2/1/1992 | 2/23/2010 | 5/31/2011 | 1/31/1995 | 5/31/2010 | 1 |
| 1082 | F | 1/1/1992 | 2/1/1992 | 11/30/2010 | 7/6/2011 | 1/1/1992 | 5/31/2010 | 1 |
| 1084 | F | 5/23/1997 | 8/29/1997 | 3/4/2013 |  | 8/29/1997 | 5/31/2010 | 1 |
| 1085 | M | 4/1/2001 | 1/5/2002 | 5/21/2012 |  | 1/5/2002 | 5/31/2010 | 1 |
| 1086 | M | 8/1/2007 | 8/11/2007 | 3/4/2013 |  | 8/1/2007 | 5/31/2010 | 1 |
| 1098 | F | 1/1/1984 | 11/15/1992 | 8/18/2003 | 12/16/2003 | 11/15/1995 | 12/16/2003 | 0 |
| 1099 | F | 6/20/1998 | 7/6/1998 | 8/18/2003 | 12/14/2003 | 6/20/1998 | 12/14/2003 | 1 |
| 1100 | M | 1/1/1972 | 5/19/1997 | 2/11/2013 |  | 5/18/2000 | 5/31/2010 | 1 |
| 1101 | M | 1/1/1977 | 11/1/1995 | 10/17/2006 | 3/4/2007 | 10/31/1998 | 3/4/2007 | 0 |
| 1107 | M | 1/1/1961 | 8/6/1991 | 10/15/1996 | 7/4/1997 | 8/5/1994 | 7/4/1997 | 0 |
| 1108 | M | 1/1/1951 | 2/11/1991 | 2/9/1997 | 7/1/1997 | 2/10/1994 | 7/1/1997 | 0 |
| 1114 | F | 1/1/1955 | 7/30/1993 | 5/21/2011 | 12/21/2011 | 7/29/1996 | 5/31/2010 | 1 |
| 1115 | M | 1/1/1991 | 7/30/1993 | 7/15/2000 | 1/11/2001 | 7/30/1993 | 1/11/2001 | 1 |
| 1116 | M | 4/29/2000 | 6/26/2000 | 5/18/2010 | 1/11/2011 | 4/29/2000 | 5/31/2010 | 1 |
| 1118 | F | 1/1/1979 | 1/25/1993 | 1/13/2013 |  | 1/25/1996 | 5/31/2010 | 1 |
| 1119 | M | 12/1/1991 | 1/25/1993 | 1/14/2006 | 2/26/2007 | 1/25/1993 | 2/26/2007 | 0 |
| 1120 | M | 3/20/1996 | 8/7/1996 | 1/19/2007 | 4/11/2008 | 8/7/1996 | 4/11/2008 | 1 |
| 1121 | F | 8/7/2000 | 1/26/2001 | 2/18/2011 | 10/8/2011 | 1/26/2001 | 5/31/2010 | 1 |
| 1123 | F | 1/1/1976 | 7/26/1993 | 10/30/2012 |  | 7/25/1996 | 5/31/2010 | 1 |
| 1124 | F | 7/1/1992 | 12/22/1992 | 11/28/2011 | 7/29/2012 | 12/22/1992 | 5/31/2010 | 1 |
| 1126 | M | 10/1/1997 | 1/13/1998 | 10/30/2012 |  | 1/13/1998 | 5/31/2010 | 1 |
| 1127 | F | 1/1/1970 | 6/5/1991 | 5/25/2012 | 10/4/2012 | 6/4/1994 | 5/31/2010 | 1 |
| 1128 | M | 4/1/1992 | 12/4/1992 | 5/11/2004 | 9/8/2004 | 12/4/1992 | 9/8/2004 | 1 |
| 1129 | M | 11/18/2000 | 3/16/2001 | 2/12/2013 |  | 3/16/2001 | 5/31/2010 | 1 |
| 1137 | M | 1/1/1935 | 10/16/1990 | 8/30/1999 | 5/26/2000 | 10/15/1993 | 5/26/2000 | 0 |
| 1139 | F | 1/1/1935 | 2/3/1991 | 12/29/2005 | 7/1/2006 | 2/2/1994 | 7/1/2006 | 0 |
| 1140 | F | 1/1/1985 | 2/3/1991 | 3/13/2013 |  | 2/2/1994 | 5/31/2010 | 1 |
| 1142 | F | 8/1/1992 | 8/15/1992 | 2/25/1996 | 8/9/1996 | 8/1/1992 | 8/9/1996 | 0 |
| 1146 | M | 1/1/1956 | 8/13/1991 | 3/23/2013 |  | 8/12/1994 | 5/31/2010 | 1 |
| 1152 | F | 1/1/1976 | 6/19/1991 | 11/26/2010 | 5/1/2011 | 6/18/1994 | 5/31/2010 | 1 |
| 1153 | M | 6/1/1990 | 6/19/1991 | 11/26/2010 | 9/18/2011 | 6/19/1991 | 5/31/2010 | 1 |
| 1154 | F | 6/10/1995 | 10/25/1995 | 11/26/2010 | 5/21/2011 | 10/25/1995 | 5/31/2010 | 1 |
| 1155 | F | 10/1/2001 | 10/31/2001 | 11/26/2010 | 6/2/2011 | 10/1/2001 | 5/31/2010 | 1 |
| 1156 | M | 7/1/2007 | 10/19/2007 | 11/26/2010 | 11/29/2011 | 10/19/2007 | 5/31/2010 | 1 |
| 1167 | M | 1/1/1946 | 12/12/1991 | 3/22/1995 | 6/28/1995 | 12/11/1994 | 6/28/1995 | 0 |
| 1176 | M | 1/1/1986 | 1/20/1993 | 3/23/2013 |  | 1/20/1996 | 5/31/2010 | 1 |
| 1178 | F | 1/1/1973 | 9/27/1991 | 10/31/2001 | 4/25/2002 | 9/26/1994 | 4/25/2002 | 0 |
| 1180 | M | 9/1/1992 | 11/20/1992 | 10/9/2004 | 4/27/2005 | 11/20/1992 | 10/9/2004 | 1 |
| 1181 | M | 10/1/1998 | 5/31/1999 | 6/28/2001 | 12/17/2001 | 5/31/1999 | 12/17/2001 | 0 |
| 1186 | F | 1/1/1967 | 11/8/1990 | 9/21/2112 |  | 11/7/1993 | 5/31/2010 | 1 |
| 1187 | M | 1/1/1984 | 11/8/1990 | 2/19/2011 | 8/28/2011 | 11/7/1993 | 5/31/2010 | 1 |
| 1188 | F | 1/1/1986 | 11/8/1990 | 4/15/2013 |  | 11/7/1993 | 5/31/2010 | 1 |
| 1189 | F | 9/2/2005 | 9/30/2005 | 4/15/2013 |  | 9/2/2005 | 5/31/2010 | 1 |
| 1191 | M | 7/20/1995 | 9/17/1995 | 11/2/2011 |  | 7/20/1995 | 5/31/2010 | 1 |
| 1192 | F | 11/1/1999 | 12/15/1999 | 1/22/2013 |  | 11/1/1999 | 5/31/2010 | 1 |
| 1193 | M | 7/1/2003 | 8/16/2003 | 1/22/2013 |  | 7/1/2003 | 5/31/2010 | 1 |
| 1194 | M | 10/1/2007 | 10/11/2007 | 9/21/2012 |  | 10/1/2007 | 5/31/2010 | 1 |
| 1195 | M | 1/1/1973 | 10/3/1991 | 12/30/2011 |  | 10/2/1994 | 5/31/2010 | 1 |
| 1211 | M | 1/1/1983 | 2/15/2001 | 1/28/2013 |  | 2/15/2004 | 5/31/2010 | 1 |
| 1216 | F | 1/1/1955 | 1/19/1991 | 6/4/2003 | 9/30/2003 | 1/18/1994 | 9/30/2003 | 0 |
| 1217 | M | 6/1/1990 | 1/19/1991 | 6/2/2003 | 10/8/2003 | 1/19/1991 | 10/8/2003 | 1 |
| 1218 | F | 12/15/1994 | 4/11/1995 | 6/4/2003 | 10/20/2003 | 4/11/1995 | 10/20/2003 | 1 |
| 1219 | M | 5/6/1998 | 5/21/1998 | 6/2/2003 | 6/5/2005 | 5/6/1998 | 6/5/2005 | 1 |
| 1220 | M | 6/7/2000 | 11/15/2000 | 6/4/2003 | 1/8/2004 | 11/15/2000 | 1/8/2004 | 0 |
| 1223 | M | 1/1/1956 | 2/10/1991 | 1/28/2012 |  | 2/9/1994 | 5/31/2010 | 1 |
| 1228 | F | 1/1/1973 | 11/11/1992 | 10/22/2004 | 7/3/2005 | 11/11/1995 | 7/3/2005 | 0 |
| 1229 | M | 7/13/1997 | 6/27/1998 | 3/23/2013 |  | 6/27/1998 | 5/31/2010 | 1 |
| 1230 | M | 8/17/2002 | 10/15/2002 | 6/7/2006 | 2/26/2007 | 8/17/2002 | 2/26/2007 | 0 |
| 1231 | M | 1/1/1957 | 8/2/1993 | 4/15/2006 | 12/25/2006 | 8/1/1996 | 12/25/2006 | 0 |
| 1233 | F | 1/1/1971 | 6/7/1999 | 4/15/2013 |  | 6/6/2002 | 5/31/2010 | 1 |
| 1234 | F | 1/1/1998 | 6/7/1999 | 4/15/2013 |  | 6/7/1999 | 5/31/2010 | 1 |
| 1235 | F | 7/30/2004 | 12/28/2004 | 4/15/2013 |  | 12/28/2004 | 5/31/2010 | 1 |
| 1241 | F | 1/1/1968 | 3/16/1993 | 2/18/2004 | 5/24/2004 | 3/15/1996 | 5/24/2004 | 0 |
| 1242 | F | 4/30/1996 | 6/18/1996 | 3/23/2013 |  | 4/30/1996 | 5/31/2010 | 1 |
| 1243 | F | 6/1/2000 | 12/7/2000 | 3/21/2013 |  | 12/7/2000 | 5/31/2010 | 1 |
| 1248 | F | 1/1/1957 | 8/31/1991 | 11/25/2012 |  | 8/30/1994 | 5/31/2010 | 1 |
| 1250 | F | 1/1/1978 | 8/11/1993 | 9/15/2011 |  | 8/10/1996 | 5/31/2010 | 1 |
| 1257 | F | 1/1/1990 | 8/11/1993 | 9/15/2011 |  | 8/10/1996 | 5/31/2010 | 1 |
| 1258 | M | 7/1/1986 | 8/11/1993 | 9/15/2011 |  | 8/10/1996 | 5/31/2010 | 1 |
| 1259 | F | 1/9/1999 | 8/4/1999 | 9/15/2011 |  | 8/4/1999 | 5/31/2010 | 1 |
| 1262 | F | 1/1/1983 | 8/31/1991 | 9/14/2012 |  | 8/30/1994 | 5/31/2010 | 1 |
| 1263 | F | 6/18/1992 | 8/8/1992 | 11/25/2012 |  | 6/18/1992 | 5/31/2010 | 1 |
| 1264 | F | 9/21/2007 | 10/21/2007 | 11/25/2012 |  | 9/21/2007 | 5/31/2010 | 1 |
| 1269 | M | 12/12/2005 | 1/3/2006 | 9/14/2012 |  | 12/12/2005 | 5/31/2010 | 1 |
| 1275 | M | 3/4/1995 | 5/9/1995 | 4/4/2008 | 11/6/2008 | 5/9/1995 | 11/6/2008 | 0 |
| 1276 | F | 12/17/2000 | 2/1/2001 | 11/14/2012 |  | 12/17/2000 | 5/31/2010 | 1 |
| 1277 | F | 10/29/2007 | 11/6/2007 | 11/25/2012 |  | 10/29/2007 | 5/31/2010 | 1 |
| 1278 | M | 4/11/2009 | 5/12/2009 | 11/25/2012 |  | 4/11/2009 | 5/31/2010 | 1 |
| 1282 | F | 1/1/1945 | 5/25/1995 | 4/25/1999 | 10/28/1999 | 5/24/1998 | 10/28/1999 | 0 |
| 1283 | F | 5/1/1993 | 5/25/1995 | 4/25/1999 | 10/28/1999 | 5/25/1995 | 10/28/1999 | 1 |
| 1284 | M | 3/28/1998 | 7/2/1998 | 4/25/1999 | 9/12/1999 | 7/2/1998 | 9/12/1999 | 0 |
| 1285 | M | 1/1/1973 | 8/6/1993 | 5/27/1999 | 2/27/2000 | 8/5/1996 | 2/27/2000 | 0 |
| 1287 | F | 1/1/1976 | 12/17/1991 | 3/8/2013 |  | 12/16/1994 | 5/31/2010 | 1 |
| 1288 | M | 1/1/1989 | 12/17/1991 | 1/31/2011 | 7/16/2011 | 12/16/1994 | 5/31/2010 | 1 |
| 1289 | F | 7/15/1992 | 7/29/1992 | 3/8/2013 |  | 7/15/1992 | 5/31/2010 | 1 |
| 1290 | F | 3/10/1997 | 4/12/1997 | 3/8/2013 |  | 3/10/1997 | 5/31/2010 | 1 |
| 1292 | F | 12/1/2001 | 3/29/2002 | 3/8/2013 |  | 3/29/2002 | 5/31/2010 | 1 |
| 1293 | M | 5/30/2006 | 11/19/2006 | 2/15/2012 | 7/14/2012 | 11/19/2006 | 5/31/2010 | 1 |
| 1299 | M | 1/21/1996 | 6/24/1996 | 7/15/2001 | 10/23/2001 | 6/24/1996 | 10/23/2001 | 1 |
| 1303 | F | 1/1/1962 | 2/22/1997 | 4/10/2002 | 2/12/2003 | 2/22/2000 | 2/12/2003 | 0 |
| 1304 | M | 2/1/1992 | 2/22/1997 | 9/18/1998 | 10/26/2001 | 2/22/1997 | 10/26/2001 | 1 |
| 1305 | M | 1/1/1996 | 2/22/1997 | 4/10/2002 | 2/12/2003 | 2/22/1997 | 2/12/2003 | 1 |
| 1306 | F | 1/1/1987 | 5/21/2000 | 4/5/2012 |  | 5/21/2003 | 5/31/2010 | 1 |
| 1307 | F | 1/1/1951 | 10/8/2002 | 9/10/2009 | 6/13/2010 | 10/7/2005 | 5/31/2010 | 1 |
| 1308 | M | 1/1/1995 | 10/8/2002 | 12/19/2004 | 2/19/2006 | 10/8/2002 | 12/19/2004 | 1 |
| 1309 | M | 7/1/2003 | 9/19/2003 | 9/10/2009 | 7/11/2010 | 9/19/2003 | 5/31/2010 | 1 |
| 1312 | M | 2/27/2002 | 5/6/2002 | 4/5/2012 |  | 5/6/2002 | 5/31/2010 | 1 |
| 1313 | F | 1/16/2008 | 4/9/2008 | 11/12/2008 | 2/2/2009 | 4/9/2008 | 2/2/2009 | 0 |
| 1316 | F | 1/1/1941 | 8/1/1991 | 12/29/1998 | 3/19/1999 | 7/31/1994 | 3/19/1999 | 0 |
| 1317 | F | 1/1/1980 | 11/19/1992 | 11/5/2010 | 3/7/2011 | 7/31/1994 | 5/31/2010 | 1 |
| 1318 | F | 1/8/2002 | 3/20/2002 | 5/10/2012 | 10/13/2012 | 3/20/2002 | 5/31/2010 | 1 |
| 1319 | F | 1/1/1988 | 8/1/1991 | 3/6/2013 |  | 7/31/1994 | 5/31/2010 | 1 |
| 1320 | M | 6/13/2003 | 8/8/2003 | 3/6/2013 |  | 6/13/2003 | 5/31/2010 | 1 |
| 1321 | M | 4/1/2007 | 7/2/2007 | 3/6/2013 |  | 7/2/2007 | 5/31/2010 | 1 |
| 1330 | M | 1/1/1951 | 1/14/1991 | 3/14/2000 | 6/28/2000 | 1/13/1994 | 6/28/2000 | 0 |
| 1332 | F | 1/1/1957 | 11/18/1992 | 2/2/1999 | 10/22/1999 | 11/18/1995 | 10/22/1999 | 0 |
| 1333 | F | 1/1/1980 | 11/18/1992 | 2/2/1999 | 11/25/1999 | 11/18/1995 | 11/25/1999 | 0 |
| 1334 | F | 6/1/1990 | 11/18/1992 | 2/2/1999 | 10/24/1999 | 11/18/1992 | 10/24/1999 | 1 |
| 1335 | M | 9/1/1994 | 1/10/1995 | 2/2/1999 | 9/8/2000 | 1/10/1995 | 9/8/2000 | 0 |
| 1336 | F | 8/29/1994 | 1/10/1995 | 2/2/1999 | 2/3/2000 | 1/10/1995 | 2/3/2000 | 0 |
| 1346 | M | 1/1/1972 | 7/14/1997 | 1/13/2004 | 5/18/2004 | 7/13/2000 | 5/18/2004 | 0 |
| 1347 | F | 1/1/1976 | 3/19/1991 | 4/20/2012 | 9/19/2012 | 3/18/1994 | 5/31/2010 | 1 |
| 1348 | F | 3/10/1999 | 5/29/1999 | 4/20/2012 | 10/9/2012 | 5/29/1999 | 5/31/2010 | 1 |
| 1349 | M | 8/27/2004 | 12/14/2004 | 4/20/2012 |  | 12/14/2004 | 5/31/2010 | 1 |
| 1356 | F | 1/1/1980 | 8/4/1993 | 2/13/2013 |  | 8/3/1996 | 5/31/2010 | 1 |
| 1360 | M | 8/25/2003 | 1/12/2004 | 2/13/2013 |  | 1/12/2004 | 5/31/2010 | 1 |
| 1361 | F | 1/1/1970 | 4/29/1997 | 4/20/2002 | 9/11/2002 | 4/28/2000 | 9/11/2002 | 0 |
| 1362 | M | 4/1/1992 | 4/29/1997 | 4/13/2001 | 9/20/2001 | 4/29/1997 | 9/20/2001 | 1 |
| 1363 | M | 4/1/1995 | 4/29/1997 | 5/8/2001 | 9/17/2001 | 4/29/1997 | 9/17/2001 | 1 |
| 1364 | M | 5/1/2000 | 5/6/2000 | 4/20/2002 | 12/4/2002 | 5/1/2000 | 12/4/2002 | 0 |
| 1365 | M | 1/1/1990 | 5/27/2002 | 3/13/2013 |  | 5/26/2005 | 5/31/2010 | 1 |
| 1368 | M | 1/1/1956 | 2/22/1991 | 8/30/1994 | 11/14/1994 | 2/21/1994 | 11/14/1994 | 0 |
| 1377 | F | 1/1/1988 | 10/22/2003 | 7/27/2009 |  | 10/21/2006 | 5/31/2010 | 1 |
| 1387 | M | 1/1/1981 | 11/25/1991 | 9/26/2002 | 5/24/2003 | 11/24/1994 | 5/24/2003 | 0 |
| 1394 | M | 1/1/1996 | 6/14/2006 | 1/6/2013 |  | 6/14/2006 | 5/31/2010 | 1 |
| 1396 | F | 1/1/1977 | 12/13/1994 | 12/3/2012 |  | 12/12/1997 | 5/31/2010 | 1 |
| 1397 | F | 1/1/1972 | 3/21/1991 | 4/26/2003 | 2/28/2004 | 3/20/1994 | 2/28/2004 | 0 |
| 1398 | M | 1/1/1989 | 3/21/1991 | 4/26/2003 | 5/28/2004 | 3/20/1994 | 5/28/2004 | 1 |
| 1399 | F | 7/1/1998 | 1/23/1999 | 4/26/2003 | 11/8/2003 | 1/23/1999 | 11/8/2003 | 0 |
| 1400 | F | 1/31/2000 | 3/16/2001 | 12/3/2012 |  | 3/16/2001 | 5/31/2010 | 1 |
| 1401 | M | 4/1/2008 | 10/16/2008 | 12/3/2012 |  | 10/16/2008 | 5/31/2010 | 1 |
| 1402 | M | 1/1/1956 | 8/19/1991 | 8/13/1995 | 12/17/1995 | 8/18/1994 | 12/17/1995 | 0 |
| 1408 | F | 1/1/1965 | 5/3/2002 | 12/29/2005 | 5/12/2006 | 5/2/2005 | 5/12/2006 | 0 |
| 1409 | M | 1/1/1997 | 5/3/2002 | 7/2/2005 | 4/16/2006 | 5/3/2002 | 4/16/2006 | 1 |
| 1410 | M | 5/1/2000 | 5/3/2002 | 12/29/2005 | 5/20/2006 | 5/3/2002 | 5/20/2006 | 1 |
| 1411 | M | 1/1/1965 | 2/4/1993 | 11/16/2012 |  | 2/4/1996 | 5/31/2010 | 1 |
| 1413 | M | 1/1/1956 | 1/2/1991 | 5/13/2001 | 2/3/2002 | 1/1/1994 | 2/3/2002 | 0 |
| 1414 | F | 1/1/1968 | 9/4/1991 | 2/11/2013 |  | 9/3/1994 | 5/31/2010 | 1 |
| 1415 | F | 1/1/1986 | 9/4/1991 | 10/20/2001 | 2/24/2003 | 9/3/1994 | 2/24/2003 | 1 |
| 1416 | M | 9/1/1991 | 9/4/1991 | 10/17/2001 | 5/23/2002 | 9/1/1991 | 5/23/2002 | 1 |
| 1417 | M | 8/17/1995 | 4/27/1996 | 1/19/2006 | 8/27/2006 | 4/27/1996 | 8/27/2006 | 1 |
| 1418 | M | 12/1/2000 | 10/5/2001 | 1/7/2013 |  | 10/5/2001 | 5/31/2010 | 1 |
| 1419 | F | 10/1/2008 | 10/10/2008 | 2/11/2013 |  | 10/1/2008 | 5/31/2010 | 1 |
| 1421 | F | 1/1/1992 | 7/22/2005 | 11/18/2010 | 2/22/2011 | 7/22/2005 | 5/31/2010 | 1 |
| 1422 | M | 7/22/2008 | 7/26/2008 | 11/18/2010 | 3/28/2011 | 7/22/2008 | 5/31/2010 | 1 |
| 1426 | M | 1/1/1969 | 10/24/1991 | 1/5/2002 | 6/6/2002 | 10/23/1994 | 6/6/2002 | 0 |
| 1430 | M | 1/1/1966 | 9/15/1992 | 11/13/2012 |  | 9/15/1995 | 5/31/2010 | 1 |
| 1431 | F | 1/1/1955 | 4/29/1993 | 8/11/2009 | 5/10/2010 | 4/28/1996 | 5/10/2010 | 0 |
| 1432 | F | 10/1/1991 | 4/29/1993 | 5/16/2008 | 3/31/2010 | 4/29/1993 | 3/31/2010 | 1 |
| 1433 | M | 6/1/1999 | 10/24/1999 | 2/4/2008 | 12/30/2008 | 10/24/1999 | 12/30/2008 | 1 |
| 1434 | M | 12/1/2006 | 3/23/2007 | 8/11/2009 | 4/12/2010 | 3/23/2007 | 4/12/2010 | 0 |
| 1437 | M | 1/1/1957 | 1/12/1992 | 12/28/2012 |  | 1/11/1995 | 5/31/2010 | 1 |
| 1439 | F | 1/1/1964 | 8/14/1991 | 12/1/2012 |  | 8/13/1994 | 5/31/2010 | 1 |
| 1440 | F | 1/1/1979 | 8/14/1991 | 11/26/2012 |  | 8/13/1994 | 5/31/2010 | 1 |
| 1441 | F | 1/1/1983 | 8/14/1991 | 11/26/2012 |  | 8/13/1994 | 5/31/2010 | 1 |
| 1448 | M | 7/6/1997 | 9/1/1997 | 11/13/2012 |  | 7/6/1997 | 5/31/2010 | 1 |
| 1449 | F | 12/15/2004 | 5/3/2005 | 12/1/2012 |  | 5/3/2005 | 5/31/2010 | 1 |
| 1451 | M | 1/1/1962 | 12/19/1992 | 2/14/2002 | 9/20/2002 | 12/19/1995 | 9/20/2002 | 0 |
| 1459 | M | 1/1/1990 | 12/31/1990 | 12/28/2006 | 6/10/2007 | 12/30/1993 | 6/10/2007 | 0 |
| 1465 | F | 1/1/1971 | 6/11/1994 | 4/15/2013 |  | 6/10/1997 | 5/31/2010 | 1 |
| 1466 | F | 3/1/1994 | 6/11/1994 | 4/15/2013 |  | 6/11/1994 | 5/31/2010 | 1 |
| 1467 | F | 8/1/2000 | 12/3/2000 | 4/15/2013 |  | 12/3/2000 | 5/31/2010 | 1 |
| 1468 | F | 1/1/2006 | 4/24/2006 | 4/15/2013 |  | 4/24/2006 | 5/31/2010 | 1 |
| 1469 | M | 1/1/1956 | 7/31/1991 | 2/4/2013 |  | 7/30/1994 | 5/31/2010 | 1 |
| 1470 | M | 1/1/1974 | 8/30/1992 | 1/3/2011 | 6/22/2011 | 8/30/1995 | 5/31/2010 | 1 |
| 1475 | F | 1/1/1968 | 4/8/2002 | 2/11/2013 |  | 4/7/2005 | 5/31/2010 | 1 |
| 1478 | F | 1/1/1990 | 4/8/2002 | 5/24/2012 |  | 4/7/2005 | 5/31/2010 | 1 |
| 1479 | M | 4/1/1997 | 4/8/2002 | 5/18/2010 |  | 4/8/2002 | 5/31/2010 | 1 |
| 1480 | M | 8/1/2004 | 12/13/2004 | 5/24/2012 |  | 12/13/2004 | 5/31/2010 | 1 |
| 1484 | M | 2/25/2003 | 1/15/2004 | 2/10/2013 |  | 1/15/2004 | 5/31/2010 | 1 |
| 1493 | F | 1/1/1948 | 6/1/1991 | 9/21/2007 | 1/13/2008 | 5/31/1994 | 1/13/2008 | 0 |
| 1495 | M | 6/1/1988 | 6/1/1991 | 4/20/2003 | 8/26/2003 | 5/31/1994 | 8/26/2003 | 0 |
| 1496 | M | 12/21/1993 | 4/7/1994 | 5/14/2002 | 3/20/2003 | 4/7/1994 | 3/20/2003 | 1 |
| 1497 | F | 12/1/1998 | 4/25/1999 | 4/15/2013 |  | 4/25/1999 | 5/31/2010 | 1 |
| 1499 | F | 1/1/1960 | 4/17/1994 | 1/27/2013 |  | 4/16/1997 | 5/31/2010 | 1 |
| 1500 | M | 1/1/1949 | 2/16/1994 | 1/6/2000 | 11/27/2000 | 2/15/1997 | 11/27/2000 | 0 |
| 1503 | M | 1/1/1951 | 2/11/1991 | 12/20/2001 | 2/26/2002 | 2/10/1994 | 2/26/2002 | 0 |
| 1504 | F | 1/1/1988 | 2/13/1998 | 2/13/2013 |  | 2/12/2001 | 5/31/2010 | 1 |
| 1505 | M | 12/19/2003 | 1/16/2004 | 2/13/2013 |  | 12/19/2003 | 5/31/2010 | 1 |
| 1508 | F | 1/1/1976 | 10/7/1991 | 4/28/2003 | 8/16/2003 | 10/6/1994 | 8/16/2003 | 0 |
| 1509 | F | 4/17/1999 | 5/4/1999 | 4/28/2003 | 7/11/2003 | 4/17/1999 | 7/11/2003 | 0 |
| 1513 | M | 1/1/1967 | 3/29/1992 | 1/12/1999 | 8/18/1999 | 3/29/1995 | 8/18/1999 | 0 |
| 1514 | F | 1/1/1943 | 1/24/1993 | 8/23/1999 | 1/22/2000 | 1/24/1996 | 1/22/2000 | 0 |
| 1515 | M | 1/1/1982 | 3/29/1992 | 4/17/1995 | 1/18/1996 | 3/29/1995 | 1/18/1996 | 0 |
| 1516 | M | 1/1/1973 | 11/28/1993 | 7/2/2009 | 12/7/2009 | 11/27/1996 | 12/7/2009 | 0 |
| 1522 | F | 1/1/1947 | 8/7/1992 | 4/17/2000 | 10/26/2000 | 8/7/1995 | 10/26/2000 | 0 |
| 1523 | M | 1/1/1974 | 8/7/1992 | 4/17/2000 | 11/1/2000 | 8/7/1995 | 11/1/2000 | 1 |
| 1524 | M | 8/1/1991 | 8/7/1992 | 8/7/1992 | 8/22/1992 | 8/7/1992 | 8/22/1992 | 0 |
| 1528 | F | 1/1/1941 | 1/14/1991 | 7/25/2002 | 9/25/2002 | 1/13/1994 | 9/25/2002 | 0 |
| 1529 | M | 7/8/1996 | 7/25/1996 | 10/31/2002 | 12/28/2002 | 7/8/1996 | 10/31/2002 | 1 |
| 1530 | F | 1/1/1983 | 7/7/1997 | 1/23/2011 | 7/24/2011 | 7/6/2000 | 5/31/2010 | 1 |
| 1532 | M | 6/1/1999 | 6/7/1999 | 10/21/2010 | 5/13/2011 | 6/1/1999 | 5/31/2010 | 1 |
| 1533 | F | 2/7/2007 | 3/5/2007 | 1/23/2011 | 8/29/2011 | 2/7/2007 | 5/31/2010 | 1 |
| 1539 | M | 1/1/1961 | 3/2/1991 | 1/17/2004 | 9/23/2004 | 3/1/1994 | 9/23/2004 | 0 |
| 1548 | F | 1/1/1982 | 2/18/1993 | 11/13/2012 |  | 2/18/1996 | 5/31/2010 | 1 |
| 1549 | M | 1/12/1998 | 4/25/1998 | 11/7/2011 | 4/9/2012 | 4/25/1998 | 5/31/2010 | 1 |
| 1550 | M | 9/1/2006 | 9/23/2007 | 11/13/2012 |  | 9/23/2007 | 5/31/2010 | 1 |
| 1565 | F | 1/1/1978 | 6/21/1994 | 3/15/2013 |  | 6/20/1997 | 5/31/2010 | 1 |
| 1566 | F | 4/6/1997 | 7/5/1997 | 3/12/2013 |  | 7/5/1997 | 5/31/2010 | 1 |
| 1567 | F | 4/15/2002 | 5/29/2002 | 3/15/2013 |  | 4/15/2002 | 5/31/2010 | 1 |
| 1568 | M | 9/1/2007 | 6/9/2008 | 3/15/2013 |  | 6/9/2008 | 5/31/2010 | 1 |
| 1570 | F | 1/1/1955 | 8/21/1991 | 5/17/2012 |  | 8/20/1994 | 5/31/2010 | 1 |
| 1571 | F | 1/1/1970 | 6/15/1994 | 3/18/2013 |  | 8/20/1994 | 5/31/2010 | 1 |
| 1572 | F | 5/1/2000 | 5/20/2000 | 1/18/2013 |  | 5/1/2000 | 5/31/2010 | 1 |
| 1573 | M | 5/1/2004 | 12/30/2004 | 3/18/2013 |  | 12/30/2004 | 5/31/2010 | 1 |
| 1575 | F | 1/1/1976 | 6/15/1994 | 3/18/2013 |  | 8/20/1994 | 5/31/2010 | 1 |
| 1577 | M | 7/1/1997 | 7/5/1997 | 11/8/2012 |  | 7/1/1997 | 5/31/2010 | 1 |
| 1578 | F | 11/21/2002 | 12/13/2002 | 11/15/2012 |  | 11/21/2002 | 5/31/2010 | 1 |
| 1579 | M | 5/15/2008 | 5/16/2008 | 5/17/2012 | 10/14/2012 | 5/15/2008 | 5/31/2010 | 1 |
| 1590 | M | 1/1/1972 | 2/23/1992 | 8/18/2003 | 3/17/2004 | 2/22/1995 | 3/17/2004 | 0 |
| 1592 | M | 1/1/1960 | 4/1/1990 | 9/19/1998 | 3/20/1999 | 3/31/1993 | 3/20/1999 | 0 |
| 1595 | F | 1/1/1958 | 1/22/1994 | 3/9/2013 |  | 1/21/1997 | 5/31/2010 | 1 |
| 1596 | F | 1/1/1983 | 1/22/1994 | 3/9/2013 |  | 1/21/1997 | 5/31/2010 | 1 |
| 1597 | F | 1/1/1987 | 1/22/1994 | 3/9/2013 |  | 1/21/1997 | 5/31/2010 | 1 |
| 1605 | F | 1/1/1992 | 1/22/1994 | 3/10/2012 |  | 1/22/1994 | 5/31/2010 | 1 |
| 1606 | M | 11/21/1994 | 9/21/1995 | 2/20/2012 |  | 9/21/1995 | 5/31/2010 | 1 |
| 1607 | M | 9/25/2001 | 3/31/2002 | 3/3/2010 | 2/20/2011 | 3/31/2002 | 5/31/2010 | 1 |
| 1608 | M | 3/1/2008 | 4/14/2009 | 3/9/2013 |  | 4/14/2009 | 5/31/2010 | 1 |
| 1613 | M | 1/1/1962 | 12/30/1992 | 3/21/2012 |  | 12/30/1995 | 5/31/2010 | 1 |
| 1626 | M | 1/1/1944 | 2/16/1994 | 3/17/1998 | 12/10/1998 | 2/15/1997 | 12/10/1998 | 0 |
| 1638 | F | 1/1/1976 | 2/14/2000 | 11/7/2011 |  | 2/13/2003 | 5/31/2010 | 1 |
| 1639 | M | 2/9/2002 | 9/25/2002 | 11/7/2011 |  | 9/25/2002 | 5/31/2010 | 1 |
| 1641 | M | 1/1/1982 | 7/15/1992 | 10/2/1997 | 4/6/2009 | 7/15/1995 | 4/6/2009 | 0 |
| 1653 | F | 1/1/1992 | 11/16/2003 | 10/13/2012 |  | 11/16/2003 | 5/31/2010 | 1 |
| 1668 | M | 1/1/1960 | 9/1/1990 | 1/9/2013 |  | 8/31/1993 | 5/31/2010 | 1 |
| 1673 | F | 1/1/1960 | 3/30/1995 | 1/8/2013 |  | 3/29/1998 | 5/31/2010 | 1 |
| 1674 | F | 1/1/1989 | 3/30/1995 | 1/8/2013 |  | 3/29/1998 | 5/31/2010 | 1 |
| 1675 | M | 3/24/1998 | 1/26/1999 | 1/3/2013 |  | 1/26/1999 | 5/31/2010 | 1 |
| 1678 | F | 6/27/2004 | 12/5/2004 | 1/8/2013 |  | 12/5/2004 | 5/31/2010 | 1 |
| 1695 | M | 1/1/2007 | 3/3/2007 | 3/22/2013 |  | 3/3/2007 | 5/31/2010 | 1 |
| 1696 | F | 1/1/1977 | 2/27/1992 | 4/15/2013 |  | 2/26/1995 | 5/31/2010 | 1 |
| 1710 | M | 1/1/1962 | 7/30/1992 | 8/23/2005 | 3/21/2006 | 7/30/1995 | 3/21/2006 | 0 |
| 1725 | F | 1/1/1976 | 9/21/1991 | 9/3/2003 | 12/6/2003 | 9/20/1994 | 12/6/2003 | 0 |
| 1726 | F | 6/19/1995 | 8/19/1995 | 9/3/2003 | 11/8/2003 | 8/19/1995 | 11/8/2003 | 1 |
| 1727 | F | 11/19/2000 | 12/7/2000 | 9/3/2003 | 11/24/2003 | 11/19/2000 | 11/24/2003 | 0 |
| 1729 | F | 1/1/1960 | 9/22/1991 | 11/4/2009 | 3/26/2010 | 9/21/1994 | 3/26/2010 | 0 |
| 1730 | F | 1/1/1981 | 9/22/1991 | 9/6/2002 | 6/9/2003 | 9/21/1994 | 6/9/2003 | 1 |
| 1731 | M | 3/1/1993 | 4/4/1993 | 8/16/2005 | 3/2/2006 | 3/1/1993 | 3/2/2006 | 1 |
| 1760 | M | 1/1/1948 | 3/18/1992 | 2/8/2008 | 11/20/2008 | 3/18/1995 | 11/20/2008 | 0 |
| 1764 | F | 1/1/1959 | 7/23/1992 | 10/8/2003 | 12/21/2003 | 7/23/1995 | 12/21/2003 | 0 |
| 1765 | M | 1/1/1980 | 7/23/1992 | 6/28/2000 | 9/6/2000 | 7/23/1995 | 9/6/2000 | 0 |
| 1766 | M | 1/1/1992 | 7/23/1992 | 10/18/2003 | 1/2/2004 | 7/23/1992 | 1/2/2004 | 0 |
| 1767 | M | 1/1/1997 | 4/4/1997 | 10/24/2003 | 1/10/2004 | 4/4/1997 | 10/24/2003 | 1 |
| 1768 | F | 5/3/2001 | 6/20/2001 | 10/24/2003 | 2/9/2004 | 5/3/2001 | 2/9/2004 | 0 |
| 1771 | M | 1/1/1940 | 10/18/1990 | 3/14/2002 | 9/10/2002 | 10/17/1993 | 9/10/2002 | 0 |
| 1772 | F | 1/1/1973 | 12/16/1992 | 3/19/2013 |  | 12/16/1995 | 5/31/2010 | 1 |
| 1775 | F | 6/26/1996 | 7/11/1996 | 11/16/2012 |  | 6/26/1996 | 5/31/2010 | 1 |
| 1776 | M | 11/8/2002 | 11/27/2002 | 11/13/2012 |  | 11/8/2002 | 5/31/2010 | 1 |
| 1777 | M | 5/1/2007 | 10/17/2007 | 5/26/2012 | 7/23/2012 | 10/17/2007 | 5/31/2010 | 1 |
| 1779 | F | 1/1/1960 | 1/31/1992 | 11/30/2012 |  | 1/30/1995 | 5/31/2010 | 1 |
| 1784 | M | 1/1/1977 | 1/31/1992 | 10/23/2006 | 3/30/2007 | 1/30/1995 | 3/30/2007 | 0 |
| 1785 | M | 1/1/1985 | 1/31/1992 | 2/8/2003 | 7/10/2003 | 1/30/1995 | 7/10/2003 | 0 |
| 1788 | F | 1/1/1987 | 1/31/1992 | 2/17/2010 | 10/1/2010 | 1/30/1995 | 5/31/2010 | 1 |
| 1789 | F | 4/1/1999 | 5/15/1999 | 11/21/2012 |  | 4/1/1999 | 5/31/2010 | 1 |
| 1790 | F | 8/1/2002 | 11/20/2002 | 11/30/2012 |  | 11/20/2002 | 5/31/2010 | 1 |
| 1791 | F | 9/16/2008 | 12/23/2008 | 11/30/2012 |  | 12/23/2008 | 5/31/2010 | 1 |
| 1795 | F | 1/1/1982 | 4/11/1994 | 9/6/1998 | 7/21/1999 | 4/10/1997 | 7/21/1999 | 0 |
| 1796 | M | 1/1/1956 | 8/27/1991 | 5/10/2001 | 10/27/2001 | 8/26/1994 | 10/27/2001 | 0 |
| 1799 | F | 1/1/1990 | 9/20/2004 | 10/13/2012 |  | 9/20/2007 | 5/31/2010 | 1 |
| 1804 | M | 1/1/1961 | 9/1/1991 | 1/23/1997 | 5/29/1997 | 8/31/1994 | 5/29/1997 | 0 |
| 1814 | F | 1/1/1941 | 6/4/1991 | 2/15/1998 | 10/1/1998 | 6/3/1994 | 10/1/1998 | 0 |
| 1815 | F | 1/1/1983 | 6/4/1991 | 2/15/1998 | 10/1/1998 | 6/3/1994 | 10/1/1998 | 1 |
| 1816 | M | 1/1/1988 | 6/4/1991 | 2/15/1998 | 10/1/1998 | 6/3/1994 | 10/1/1998 | 1 |
| 1821 | M | 1/1/1967 | 2/4/1992 | 9/10/1997 | 8/14/1998 | 2/3/1995 | 8/14/1998 | 0 |
| 1825 | F | 1/1/1963 | 1/24/1991 | 2/26/2013 |  | 1/23/1994 | 5/31/2010 | 1 |
| 1829 | F | 1/1/1990 | 1/24/1991 | 3/8/2013 |  | 1/23/1994 | 5/31/2010 | 1 |
| 1830 | F | 1/1/1988 | 1/5/1996 | 3/23/2010 | 5/21/2011 | 1/5/1996 | 5/31/2010 | 1 |
| 1831 | M | 4/1/2007 | 12/11/2007 | 3/8/2013 |  | 12/11/2007 | 5/31/2010 | 1 |
| 1832 | M | 9/1/1999 | 11/4/2000 | 1/15/2010 | 5/24/2011 | 11/4/2000 | 5/31/2010 | 1 |
| 1833 | F | 6/6/2004 | 9/24/2005 | 2/26/2013 |  | 9/24/2005 | 5/31/2010 | 1 |
| 1839 | F | 1/1/1952 | 7/21/1992 | 6/5/2000 | 5/21/2001 | 7/21/1995 | 5/21/2001 | 0 |
| 1840 | M | 7/1/1991 | 7/21/1992 | 9/19/1997 | 5/3/1998 | 7/21/1992 | 5/3/1998 | 0 |
| 1849 | F | 1/1/1955 | 3/30/1995 | 7/23/1998 | 1/11/1999 | 3/29/1998 | 1/11/1999 | 0 |
| 1850 | M | 3/1/1989 | 3/30/1995 | 6/3/1998 | 3/18/1999 | 3/29/1998 | 3/18/1999 | 0 |
| 1851 | F | 3/1/1993 | 3/30/1995 | 7/23/1998 | 1/11/1999 | 3/30/1995 | 1/11/1999 | 1 |
| 1852 | F | 2/1/1997 | 4/21/1997 | 7/23/1998 | 10/31/1998 | 4/21/1997 | 10/31/1998 | 0 |
| 1856 | M | 1/1/1980 | 8/10/2005 | 3/9/2010 | 6/22/2011 | 8/9/2008 | 5/31/2010 | 1 |
| 1857 | F | 1/1/1942 | 7/21/1992 | 3/21/2001 | 7/14/2002 | 7/21/1995 | 7/14/2002 | 0 |
| 1858 | M | 7/1/1986 | 7/21/1992 | 8/14/1999 | 5/9/2001 | 7/21/1995 | 5/9/2001 | 1 |
| 1859 | M | 7/1/1990 | 2/6/1994 | 3/21/2001 | 8/4/2003 | 2/6/1994 | 8/4/2003 | 1 |
| 1860 | M | 7/1/1995 | 7/11/1996 | 3/21/2001 | 2/21/2003 | 7/11/1996 | 2/21/2003 | 1 |
| 1867 | M | 1/1/1960 | 12/5/1991 | 2/13/2013 |  | 12/4/1994 | 5/31/2010 | 1 |
| 1868 | F | 1/1/1956 | 9/29/2005 | 5/19/2011 | 11/23/2011 | 9/28/2008 | 5/31/2010 | 1 |
| 1869 | F | 1/1/1996 | 10/28/2006 | 5/19/2011 | 12/5/2011 | 10/28/2006 | 5/31/2010 | 1 |
| 1872 | M | 1/1/1957 | 8/8/1992 | 7/1/2000 | 12/4/2000 | 8/8/1995 | 12/4/2000 | 0 |
| 1884 | F | 1/1/1942 | 7/25/1992 | 11/6/1997 | 4/27/1998 | 7/25/1995 | 4/27/1998 | 0 |
| 1885 | M | 6/1/1986 | 7/25/1992 | 11/6/1997 | 6/18/1998 | 7/25/1995 | 6/18/1998 | 1 |
| 1887 | F | 1/1/1942 | 11/3/1992 | 11/1/2000 | 4/10/2001 | 11/3/1995 | 4/10/2001 | 0 |
| 1888 | M | 1/1/1989 | 11/3/1992 | 10/7/2012 |  | 11/3/1995 | 5/31/2010 | 1 |
| 1889 | M | 1/1/1994 | 4/30/1994 | 5/16/2005 | 2/22/2006 | 4/30/1994 | 2/22/2006 | 0 |
| 1893 | F | 1/1/1957 | 2/2/1992 | 12/1/2012 |  | 2/1/1995 | 5/31/2010 | 1 |
| 1894 | M | 1/1/1989 | 2/2/1992 | 12/17/2010 | 10/3/2011 | 2/1/1995 | 5/31/2010 | 1 |
| 1895 | F | 6/1/1994 | 1/23/1995 | 12/17/2010 | 9/7/2011 | 1/23/1995 | 5/31/2010 | 1 |
| 1896 | M | 4/23/2006 | 7/30/2006 | 8/5/2006 | 10/8/2006 | 7/30/2006 | 10/8/2006 | 0 |
| 1897 | M | 1/1/1968 | 1/18/1993 | 2/22/2010 | 3/17/2011 | 1/18/1996 | 5/31/2010 | 1 |
| 1906 | F | 1/1/1964 | 4/13/1993 | 10/19/2012 |  | 4/12/1996 | 5/31/2010 | 1 |
| 1907 | F | 1/1/1986 | 4/13/1993 | 11/10/2011 |  | 4/12/1996 | 5/31/2010 | 1 |
| 1909 | M | 1/1/1995 | 9/22/1995 | 1/7/2013 |  | 9/22/1995 | 5/31/2010 | 1 |
| 1910 | F | 3/1/2001 | 5/22/2001 | 10/9/2012 |  | 5/22/2001 | 5/31/2010 | 1 |
| 1911 | M | 5/1/2003 | 9/21/2003 | 4/16/2011 | 2/14/2012 | 9/21/2003 | 5/31/2010 | 1 |
| 1912 | M | 5/13/2006 | 5/23/2006 | 10/9/2012 |  | 5/13/2006 | 5/31/2010 | 1 |
| 1913 | F | 1/1/1969 | 10/7/1991 | 3/22/2013 |  | 10/6/1994 | 5/31/2010 | 1 |
| 1914 | F | 1/1/1983 | 10/7/1991 | 5/24/2001 | 9/13/2001 | 10/6/1994 | 9/13/2001 | 0 |
| 1915 | F | 1/1/1991 | 10/7/1991 | 3/21/2013 |  | 10/7/1991 | 5/31/2010 | 1 |
| 1918 | F | 11/1/1996 | 3/30/1997 | 2/6/2011 | 6/8/2011 | 3/30/1997 | 5/31/2010 | 1 |
| 1919 | M | 9/1/2001 | 2/26/2002 | 3/21/2013 |  | 2/26/2002 | 5/31/2010 | 1 |
| 1920 | F | 2/1/2006 | 8/7/2006 | 3/22/2013 |  | 8/7/2006 | 5/31/2010 | 1 |
| 1921 | F | 1/1/1965 | 1/2/1991 | 1/7/2011 | 6/2/2011 | 1/1/1994 | 5/31/2010 | 1 |
| 1922 | F | 1/1/1985 | 1/2/1991 | 1/16/2013 |  | 1/1/1994 | 5/31/2010 | 1 |
| 1923 | F | 1/18/2007 | 4/22/2007 | 1/16/2013 |  | 4/22/2007 | 5/31/2010 | 1 |
| 1924 | F | 1/1/1989 | 1/2/1991 | 12/5/2000 | 6/13/2001 | 1/1/1994 | 6/13/2001 | 1 |
| 1925 | F | 1/3/1991 | 1/5/1991 | 11/24/2005 | 8/25/2006 | 1/3/1991 | 8/25/2006 | 0 |
| 1926 | M | 6/8/1996 | 8/11/1996 | 1/5/2007 | 6/14/2007 | 8/11/1996 | 6/14/2007 | 1 |
| 1927 | M | 8/10/2000 | 12/3/2000 | 11/25/2008 | 4/4/2009 | 12/3/2000 | 4/4/2009 | 1 |
| 1928 | M | 11/25/2004 | 12/27/2004 | 1/7/2011 | 7/2/2011 | 11/25/2004 | 5/31/2010 | 1 |
| 1929 | M | 7/22/2008 | 7/23/2008 | 1/7/2011 | 6/26/2011 | 7/22/2008 | 5/31/2010 | 1 |
| 1932 | F | 1/1/1965 | 6/17/2002 | 10/26/2012 |  | 6/16/2005 | 5/31/2010 | 1 |
| 1935 | M | 6/1/1996 | 6/17/2002 | 1/25/2007 | 3/16/2008 | 6/17/2002 | 3/16/2008 | 1 |
| 1936 | F | 1/1/1999 | 6/17/2002 | 10/26/2012 |  | 6/17/2002 | 5/31/2010 | 1 |
| 1937 | F | 4/13/2006 | 8/14/2006 | 10/26/2012 |  | 8/14/2006 | 5/31/2010 | 1 |
| 1938 | F | 1/1/1968 | 9/16/1991 | 5/13/2001 | 9/6/2001 | 9/15/1994 | 9/6/2001 | 0 |
| 1939 | M | 1/1/1977 | 9/16/1991 | 12/16/2007 | 7/13/2008 | 9/15/1994 | 7/13/2008 | 0 |
| 1940 | M | 2/1/1993 | 8/1/1995 | 5/13/2001 | 7/26/2001 | 8/1/1995 | 7/26/2001 | 0 |
| 1941 | M | 9/30/1998 | 12/16/1998 | 5/13/2001 | 7/10/2001 | 12/16/1998 | 7/10/2001 | 0 |
| 1942 | M | 1/1/1960 | 11/25/1990 | 11/29/1999 | 3/12/2000 | 11/24/1993 | 3/12/2000 | 0 |
| 1945 | F | 1/1/1971 | 1/14/1992 | 3/15/2013 |  | 1/13/1995 | 5/31/2010 | 1 |
| 1946 | M | 1/1/1988 | 1/14/1992 | 2/20/2012 |  | 1/13/1995 | 5/31/2010 | 1 |
| 1947 | F | 6/1/1995 | 10/25/1995 | 2/22/2012 |  | 10/25/1995 | 5/31/2010 | 1 |
| 1948 | M | 1/1/2006 | 1/9/2007 | 3/15/2013 |  | 1/9/2007 | 5/31/2010 | 1 |
| 1975 | F | 1/1/1970 | 1/25/1991 | 11/20/2012 |  | 1/24/1994 | 5/31/2010 | 1 |
| 1977 | F | 1/1/1986 | 1/25/1991 | 11/20/2012 |  | 1/24/1994 | 5/31/2010 | 1 |
| 1978 | F | 3/18/1996 | 3/20/1996 | 11/1/2012 |  | 3/18/1996 | 5/31/2010 | 1 |
| 1982 | F | 6/4/2000 | 6/28/2000 | 11/23/2011 | 3/26/2012 | 6/4/2000 | 5/31/2010 | 1 |
| 1983 | M | 6/30/2004 | 9/3/2004 | 11/23/2011 | 4/7/2012 | 9/3/2004 | 5/31/2010 | 1 |
| 1984 | M | 5/1/2008 | 6/30/2008 | 11/20/2012 |  | 6/30/2008 | 5/31/2010 | 1 |
| 1989 | F | 1/1/1936 | 1/31/1991 | 2/24/1994 | 6/30/1994 | 1/30/1994 | 6/30/1994 | 0 |
| 1990 | F | 1/1/1973 | 1/6/1993 | 2/7/2013 |  | 9/8/1996 | 5/31/2010 | 1 |
| 1991 | M | 1/1/1987 | 1/6/1993 | 4/1/2007 | 8/27/2008 | 9/8/1996 | 8/27/2008 | 1 |
| 1992 | F | 1/1/1991 | 1/6/1993 | 9/21/2010 | 6/18/2011 | 1/6/1993 | 5/31/2010 | 1 |
| 1994 | F | 7/3/1996 | 9/30/1996 | 2/7/2013 |  | 9/30/1996 | 5/31/2010 | 1 |
| 1995 | F | 7/15/2004 | 10/9/2004 | 2/7/2013 |  | 10/9/2004 | 5/31/2010 | 1 |
| 1996 | F | 6/2/2007 | 8/3/2007 | 2/7/2013 |  | 8/3/2007 | 5/31/2010 | 1 |
| 1997 | M | 1/1/1960 | 8/5/1991 | 12/3/2012 |  | 8/4/1994 | 5/31/2010 | 1 |
| 2008 | F | 1/1/1952 | 1/14/1991 | 9/30/2007 | 10/20/2008 | 1/13/1994 | 10/20/2008 | 0 |
| 2009 | M | 8/12/1994 | 1/23/1995 | 12/30/2006 | 7/24/2008 | 1/23/1995 | 7/24/2008 | 1 |
| 2011 | F | 1/1/1951 | 6/1/1991 | 4/20/2003 | 3/23/2004 | 5/31/1994 | 3/23/2004 | 0 |
| 2012 | M | 2/1/1994 | 4/16/1994 | 4/20/2003 | 3/27/2004 | 4/16/1994 | 3/27/2004 | 1 |
| 2022 | F | 1/1/1982 | 10/3/2002 | 2/10/2013 |  | 10/2/2005 | 5/31/2010 | 1 |
| 2023 | F | 1/1/1997 | 10/3/2002 | 2/23/2010 | 5/11/2011 | 10/3/2002 | 5/31/2010 | 1 |
| 2024 | F | 10/1/2001 | 10/3/2002 | 2/10/2013 |  | 10/3/2002 | 5/31/2010 | 1 |
| 2026 | F | 1/1/1973 | 12/1/1992 | 4/24/1998 | 8/18/1998 | 12/1/1995 | 8/18/1998 | 0 |
| 2028 | F | 1/1/1978 | 12/1/1992 | 12/25/1997 | 6/9/1998 | 12/1/1995 | 6/9/1998 | 1 |
| 2029 | M | 1/1/1988 | 12/1/1992 | 4/24/1998 | 8/14/1998 | 12/1/1995 | 8/14/1998 | 1 |
| 2030 | M | 6/1/1991 | 12/1/1992 | 4/24/1998 | 8/18/1998 | 12/1/1992 | 8/18/1998 | 1 |
| 2031 | F | 8/3/1997 | 11/29/1997 | 4/24/1998 | 8/8/1998 | 11/29/1997 | 8/8/1998 | 0 |
| 2036 | F | 1/1/1963 | 3/28/1992 | 6/9/2005 | 10/23/2005 | 3/28/1995 | 10/23/2005 | 0 |
| 2037 | F | 1/1/1980 | 11/24/1993 | 12/13/2007 | 6/26/2008 | 3/28/1995 | 12/13/2007 | 1 |
| 2038 | F | 1/1/1982 | 3/28/1992 | 11/4/2012 |  | 3/28/1995 | 5/31/2010 | 1 |
| 2047 | F | 1/1/1986 | 3/28/1992 | 2/17/2000 | 7/16/2000 | 3/28/1995 | 2/17/2000 | 1 |
| 2049 | F | 1/1/1992 | 3/28/1992 | 1/1/2013 |  | 3/28/1992 | 5/31/2010 | 1 |
| 2050 | M | 2/1/1999 | 4/26/1999 | 6/9/2005 | 10/29/2005 | 4/26/1999 | 10/29/2005 | 1 |
| 2051 | F | 1/1/1983 | 6/17/1994 | 12/14/2001 | 10/22/2002 | 6/16/1997 | 10/22/2002 | 0 |
| 2052 | F | 6/1/1994 | 6/17/1994 | 2/7/2001 | 11/26/2001 | 6/1/1994 | 11/26/2001 | 1 |
| 2053 | M | 7/8/2000 | 2/6/2001 | 12/14/2001 | 8/26/2003 | 2/6/2001 | 8/26/2003 | 0 |
| 2054 | F | 1/1/1978 | 5/4/1993 | 12/9/1998 | 3/5/1999 | 5/3/1996 | 3/5/1999 | 0 |
| 2055 | F | 9/30/1997 | 10/2/1997 | 12/9/1998 | 6/23/1999 | 9/30/1997 | 6/23/1999 | 0 |
| 2057 | M | 1/1/1956 | 3/22/1991 | 10/27/1995 | 7/29/1996 | 3/21/1994 | 7/29/1996 | 0 |
| 2071 | F | 1/1/1960 | 12/5/1991 | 3/8/2013 |  | 12/4/1994 | 5/31/2010 | 1 |
| 2072 | F | 1/1/1975 | 12/2/1992 | 1/15/2013 |  | 12/4/1994 | 5/31/2010 | 1 |
| 2073 | F | 6/1/1990 | 12/5/1991 | 3/8/2013 |  | 12/5/1991 | 5/31/2010 | 1 |
| 2075 | F | 2/1/2007 | 3/4/2007 | 3/8/2013 |  | 2/1/2007 | 5/31/2010 | 1 |
| 2080 | M | 3/1/2000 | 12/10/2000 | 9/26/2011 | 3/14/2012 | 12/10/2000 | 5/31/2010 | 1 |
| 2083 | M | 10/20/2004 | 12/16/2004 | 1/15/2013 |  | 10/20/2004 | 5/31/2010 | 1 |
| 2084 | F | 8/19/1998 | 12/15/1998 | 11/13/2012 |  | 12/15/1998 | 5/31/2010 | 1 |
| 2085 | F | 9/1/2004 | 12/15/2004 | 5/3/2012 |  | 12/15/2004 | 5/31/2010 | 1 |
| 2090 | F | 1/1/1970 | 8/27/1991 | 3/15/2013 |  | 8/26/1994 | 5/31/2010 | 1 |
| 2091 | F | 1/1/1989 | 8/27/1991 | 3/22/2013 |  | 8/26/1994 | 5/31/2010 | 1 |
| 2092 | F | 2/1/1999 | 4/6/1999 | 3/18/2013 |  | 4/6/1999 | 5/31/2010 | 1 |
| 2095 | M | 3/1/2005 | 5/1/2005 | 2/24/2012 | 6/29/2012 | 5/1/2005 | 5/31/2010 | 1 |
| 2115 | M | 1/1/1936 | 1/7/1991 | 3/14/1995 | 9/30/1995 | 1/6/1994 | 9/30/1995 | 0 |
| 2123 | M | 10/1/2000 | 10/12/2006 | 3/12/2013 |  | 10/12/2006 | 5/31/2010 | 1 |
| 2124 | M | 4/13/2008 | 10/14/2008 | 3/12/2013 |  | 10/14/2008 | 5/31/2010 | 1 |
| 2125 | F | 1/1/1961 | 4/26/1997 | 10/18/2012 |  | 4/25/2000 | 5/31/2010 | 1 |
| 2126 | F | 1/1/1987 | 4/26/1997 | 10/18/2012 |  | 4/25/2000 | 5/31/2010 | 1 |
| 2127 | F | 1/1/1991 | 4/26/1997 | 2/22/2012 |  | 4/26/1997 | 5/31/2010 | 1 |
| 2133 | M | 11/16/1997 | 5/24/1998 | 12/16/2010 | 2/3/2012 | 5/24/1998 | 5/31/2010 | 1 |
| 2134 | F | 8/25/1999 | 1/4/2000 | 12/6/2008 | 8/1/2009 | 1/4/2000 | 8/1/2009 | 1 |
| 2135 | F | 7/25/2003 | 10/3/2003 | 3/10/2012 |  | 10/3/2003 | 5/31/2010 | 1 |
| 2136 | M | 2/13/2009 | 4/24/2009 | 3/10/2012 | 8/31/2012 | 4/24/2009 | 5/31/2010 | 1 |
| 2137 | F | 1/1/1987 | 3/3/1993 | 12/24/2011 |  | 3/2/1996 | 5/31/2010 | 1 |
| 2138 | F | 10/1/1999 | 12/3/2000 | 1/7/2011 |  | 12/3/2000 | 5/31/2010 | 1 |
| 2150 | F | 1/1/1951 | 1/5/1991 | 7/1/2001 | 11/10/2001 | 1/4/1994 | 11/10/2001 | 0 |
| 2151 | M | 1/1/1987 | 1/5/1991 | 8/13/1997 | 7/21/1998 | 1/4/1994 | 7/21/1998 | 1 |
| 2152 | M | 6/1/1990 | 1/5/1991 | 7/1/2001 | 11/14/2001 | 1/5/1991 | 11/14/2001 | 1 |
| 2164 | M | 1/1/1970 | 2/18/1993 | 1/1/2013 |  | 2/18/1996 | 5/31/2010 | 1 |
| 2223 | F | 1/1/1962 | 11/15/1994 | 10/10/2012 |  | 11/14/1997 | 5/31/2010 | 1 |
| 2224 | F | 1/1/1967 | 9/10/2002 | 10/12/2012 |  | 9/9/2005 | 5/31/2010 | 1 |
| 2227 | M | 6/1/2001 | 9/10/2002 | 5/7/2009 | 8/19/2009 | 9/10/2002 | 8/19/2009 | 0 |
| 2228 | M | 3/27/2006 | 6/27/2006 | 5/7/2009 | 7/20/2009 | 6/27/2006 | 7/20/2009 | 0 |
| 2229 | M | 1/1/1987 | 10/8/1992 | 11/28/2010 | 8/9/2011 | 11/14/1997 | 5/31/2010 | 1 |
| 2230 | F | 1/1/1991 | 11/15/1994 | 11/12/2012 |  | 11/15/1994 | 5/31/2010 | 1 |
| 2235 | M | 8/15/1996 | 10/2/1996 | 5/9/2011 | 1/14/2012 | 8/15/1996 | 5/31/2010 | 1 |
| 2237 | F | 7/13/2000 | 7/21/2000 | 10/12/2012 |  | 7/13/2000 | 5/31/2010 | 1 |
| 2238 | F | 5/1/2003 | 9/29/2005 | 5/10/2012 | 10/15/2012 | 9/29/2005 | 5/31/2010 | 1 |
| 2239 | M | 3/25/2008 | 5/28/2008 | 2/5/2013 |  | 5/28/2008 | 5/31/2010 | 1 |
| 2241 | F | 1/1/1960 | 3/8/1992 | 2/13/2013 |  | 3/8/1995 | 5/31/2010 | 1 |
| 2242 | F | 1/1/1990 | 3/8/1992 | 2/28/2012 |  | 3/8/1995 | 5/31/2010 | 1 |
| 2243 | F | 4/1/1995 | 8/1/1995 | 2/13/2013 |  | 8/1/1995 | 5/31/2010 | 1 |
| 2244 | F | 10/13/1999 | 1/6/2000 | 2/13/2013 |  | 1/6/2000 | 5/31/2010 | 1 |
| 2246 | M | 1/1/1990 | 12/7/2002 | 2/10/2012 | 8/28/2012 | 12/6/2005 | 5/31/2010 | 1 |
| 2248 | F | 1/1/1969 | 5/1/1990 | 3/15/2013 |  | 4/30/1993 | 5/31/2010 | 1 |
| 2249 | F | 1/1/1984 | 5/1/1990 | 3/8/2013 |  | 4/30/1993 | 5/31/2010 | 1 |
| 2250 | M | 9/19/2005 | 7/14/2006 | 3/8/2013 |  | 7/14/2006 | 5/31/2010 | 1 |
| 2252 | M | 1/1/1987 | 5/1/1990 | 2/9/2000 | 5/27/2000 | 4/30/1993 | 5/27/2000 | 1 |
| 2264 | M | 3/28/1991 | 10/20/1991 | 3/12/2002 | 6/6/2002 | 10/20/1991 | 6/6/2002 | 1 |
| 2266 | M | 7/2/1995 | 9/7/1995 | 2/11/2013 |  | 9/7/1995 | 5/31/2010 | 1 |
| 2267 | F | 10/28/1999 | 2/4/2000 | 3/14/2013 |  | 2/4/2000 | 5/31/2010 | 1 |
| 2268 | F | 7/8/2003 | 10/28/2003 | 3/15/2013 |  | 10/28/2003 | 5/31/2010 | 1 |
| 2269 | F | 10/25/2008 | 12/30/2008 | 3/15/2013 |  | 12/30/2008 | 5/31/2010 | 1 |
| 2270 | M | 1/1/1984 | 1/31/2004 | 2/12/2011 |  | 1/30/2007 | 5/31/2010 | 1 |
| 2280 | M | 1/1/1945 | 1/14/1992 | 8/15/1996 | 12/29/1996 | 1/13/1995 | 12/29/1996 | 0 |
| 2281 | M | 1/1/1968 | 1/18/1993 | 2/4/2013 |  | 1/18/1996 | 5/31/2010 | 1 |
| 2286 | M | 1/1/1941 | 10/30/1991 | 1/6/1998 | 10/13/1998 | 10/29/1994 | 10/13/1998 | 0 |
| 2290 | M | 1/1/1970 | 8/21/1992 | 12/23/1997 | 5/2/1998 | 8/21/1995 | 5/2/1998 | 0 |
| 2291 | F | 1/1/1971 | 3/3/1993 | 2/24/2013 |  | 3/2/1996 | 5/31/2010 | 1 |
| 2293 | F | 3/1/1993 | 3/3/1993 | 2/24/2013 |  | 3/1/1993 | 5/31/2010 | 1 |
| 2295 | M | 11/2/1999 | 2/2/2000 | 2/24/2013 |  | 2/2/2000 | 5/31/2010 | 1 |
| 2296 | M | 6/1/2006 | 9/21/2006 | 2/24/2013 |  | 9/21/2006 | 5/31/2010 | 1 |
| 2303 | M | 1/1/1982 | 6/12/2002 | 12/28/2006 | 10/14/2008 | 6/11/2005 | 10/14/2008 | 0 |
| 2308 | F | 1/1/1978 | 2/18/1998 | 6/21/2001 | 4/21/2002 | 2/17/2001 | 4/21/2002 | 0 |
| 2309 | F | 9/1/1997 | 2/18/1998 | 6/21/2001 | 4/21/2002 | 2/18/1998 | 4/21/2002 | 0 |
| 2310 | M | 1/1/1967 | 1/1/1992 | 1/6/1998 | 5/26/1998 | 12/31/1994 | 5/26/1998 | 0 |
| 2311 | F | 1/1/1955 | 10/26/1990 | 11/1/1995 | 1/8/1996 | 10/25/1993 | 1/8/1996 | 0 |
| 2313 | F | 1/1/1973 | 10/26/1990 | 2/24/2000 | 5/26/2000 | 10/25/1993 | 5/26/2000 | 0 |
| 2314 | F | 1/1/1976 | 10/26/1990 | 3/21/2000 | 7/3/2000 | 10/25/1993 | 7/3/2000 | 0 |
| 2317 | M | 2/3/1991 | 3/5/1991 | 4/12/2002 | 9/19/2002 | 2/3/1991 | 9/19/2002 | 0 |
| 2318 | F | 5/24/1995 | 7/29/1995 | 11/1/1995 | 11/27/1995 | 7/29/1995 | 11/27/1995 | 0 |
| 2319 | F | 1/1/1983 | 5/9/1993 | 2/12/2013 |  | 5/8/1996 | 5/31/2010 | 1 |
| 2320 | M | 11/24/1999 | 5/5/2000 | 1/27/2013 |  | 5/5/2000 | 5/31/2010 | 1 |
| 2321 | F | 5/1/2007 | 1/22/2008 | 2/12/2013 |  | 1/22/2008 | 5/31/2010 | 1 |
| 2330 | F | 1/1/1980 | 12/15/1992 | 12/19/2011 |  | 12/15/1995 | 5/31/2010 | 1 |
| 2334 | F | 12/1/1995 | 4/8/1996 | 12/19/2011 |  | 4/8/1996 | 5/31/2010 | 1 |
| 2335 | F | 9/1/1999 | 1/12/2000 | 12/19/2011 |  | 1/12/2000 | 5/31/2010 | 1 |
| 2336 | M | 12/13/2008 | 1/2/2009 | 12/18/2011 |  | 12/13/2008 | 5/31/2010 | 1 |
| 2339 | F | 1/1/1966 | 6/24/1996 | 2/5/2013 |  | 6/24/1999 | 5/31/2010 | 1 |
| 2340 | F | 1/1/1984 | 6/24/1996 | 5/5/2011 |  | 6/24/1999 | 5/31/2010 | 1 |
| 2341 | M | 6/1/1990 | 6/24/1996 | 7/26/1999 | 1/14/2000 | 6/24/1996 | 1/14/2000 | 1 |
| 2345 | F | 8/1/1995 | 6/24/1996 | 2/5/2013 |  | 6/24/1996 | 5/31/2010 | 1 |
| 2346 | F | 8/30/2002 | 11/14/2002 | 2/5/2013 |  | 11/14/2002 | 5/31/2010 | 1 |
| 2347 | F | 3/18/2006 | 6/6/2006 | 2/5/2013 |  | 6/6/2006 | 5/31/2010 | 1 |
| 2353 | F | 1/1/1960 | 6/5/1991 | 1/16/2013 |  | 6/4/1994 | 5/31/2010 | 1 |
| 2355 | F | 1/1/1979 | 1/15/1994 | 3/15/2013 |  | 6/4/1994 | 5/31/2010 | 1 |
| 2356 | M | 1/1/1985 | 6/5/1991 | 12/25/2006 | 7/29/2007 | 6/4/1994 | 7/29/2007 | 0 |
| 2362 | F | 1/12/1996 | 10/11/1996 | 12/28/2012 |  | 10/11/1996 | 5/31/2010 | 1 |
| 2363 | M | 10/1/2006 | 11/21/2006 | 1/16/2013 |  | 10/1/2006 | 5/31/2010 | 1 |
| 2367 | F | 1/1/1981 | 3/13/2003 | 11/26/2011 | 7/13/2012 | 3/12/2006 | 5/31/2010 | 1 |
| 2368 | M | 1/12/2006 | 5/15/2006 | 11/26/2011 | 7/15/2012 | 5/15/2006 | 5/31/2010 | 1 |
| 2374 | F | 1/1/1973 | 11/15/1994 | 10/30/2012 |  | 11/14/1997 | 5/31/2010 | 1 |
| 2375 | M | 6/1/1992 | 11/15/1994 | 11/5/2007 | 10/18/2008 | 11/15/1994 | 10/18/2008 | 1 |
| 2376 | F | 2/27/1999 | 5/15/1999 | 10/15/2012 |  | 5/15/1999 | 5/31/2010 | 1 |
| 2378 | M | 3/1/2003 | 11/21/2003 | 10/30/2012 |  | 11/21/2003 | 5/31/2010 | 1 |
| 2380 | M | 1/1/1970 | 1/3/1991 | 1/28/2013 |  | 1/2/1994 | 5/31/2010 | 1 |
| 2381 | F | 1/1/1988 | 1/29/1991 | 5/29/2011 | 6/5/2011 | 1/28/1994 | 5/31/2010 | 1 |
| 2383 | M | 5/1/2003 | 1/25/2007 | 5/27/2010 | 11/11/2010 | 1/25/2007 | 5/31/2010 | 1 |
| 2388 | M | 1/1/1966 | 2/3/1991 | 8/5/1999 | 5/15/2000 | 2/2/1994 | 5/15/2000 | 0 |
| 2405 | F | 1/1/1966 | 8/23/1994 | 11/18/2012 |  | 8/22/1997 | 5/31/2010 | 1 |
| 2408 | F | 1/1/1986 | 8/23/1994 | 11/18/2012 |  | 8/22/1997 | 5/31/2010 | 1 |
| 2409 | M | 5/1/1994 | 8/23/1994 | 11/14/2012 |  | 8/23/1994 | 5/31/2010 | 1 |
| 2414 | F | 7/1/1999 | 7/29/1999 | 11/18/2012 |  | 7/1/1999 | 5/31/2010 | 1 |
| 2415 | M | 6/1/2003 | 1/25/2004 | 11/11/2012 |  | 1/25/2004 | 5/31/2010 | 1 |
| 2416 | M | 12/28/2007 | 1/4/2008 | 11/18/2012 |  | 12/28/2007 | 5/31/2010 | 1 |
| 2418 | F | 1/1/1964 | 11/8/1990 | 4/21/1999 | 7/18/1999 | 11/7/1993 | 7/18/1999 | 0 |
| 2419 | F | 1/1/1990 | 11/8/1990 | 4/6/2002 | 8/4/2002 | 11/7/1993 | 8/4/2002 | 1 |
| 2420 | F | 4/8/1994 | 4/23/1994 | 4/15/2013 |  | 4/8/1994 | 5/31/2010 | 1 |
| 2421 | F | 4/8/1994 | 3/12/2002 | 4/15/2013 |  | 3/12/2002 | 5/31/2010 | 1 |
| 2422 | M | 1/1/1956 | 11/1/1990 | 10/16/2012 |  | 10/31/1993 | 5/31/2010 | 1 |
| 2426 | M | 1/1/1940 | 10/15/1990 | 1/30/1994 | 8/16/1994 | 10/14/1993 | 8/16/1994 | 0 |
| 2427 | M | 1/1/1952 | 11/13/1990 | 11/28/2012 |  | 11/12/1993 | 5/31/2010 | 1 |
| 2430 | M | 1/1/1971 | 5/13/1996 | 3/20/2002 | 12/15/2002 | 5/13/1999 | 12/15/2002 | 0 |
| 2433 | F | 1/1/1971 | 5/5/1998 | 1/16/2013 |  | 5/4/2001 | 5/31/2010 | 1 |
| 2434 | F | 1/1/1986 | 5/5/1998 | 10/11/2012 |  | 5/4/2001 | 5/31/2010 | 1 |
| 2435 | M | 1/1/1996 | 7/8/1998 | 12/5/2010 | 10/31/2011 | 7/8/1998 | 5/31/2010 | 1 |
| 2436 | M | 12/12/2001 | 5/9/2002 | 12/23/2010 | 9/3/2011 | 5/9/2002 | 5/31/2010 | 1 |
| 2437 | F | 10/5/2008 | 11/28/2008 | 1/16/2013 |  | 10/5/2008 | 5/31/2010 | 1 |
| 2451 | M | 1/1/1972 | 12/17/1992 | 1/5/2001 | 11/19/2001 | 12/17/1995 | 11/19/2001 | 0 |
| 2454 | F | 1/1/1967 | 4/12/1999 | 3/18/2013 |  | 4/11/2002 | 5/31/2010 | 1 |
| 2455 | F | 1/1/1971 | 2/5/2000 | 3/22/2013 |  | 2/4/2003 | 5/31/2010 | 1 |
| 2456 | M | 1/1/1994 | 2/5/2000 | 2/24/2012 |  | 2/5/2000 | 5/31/2010 | 1 |
| 2457 | F | 9/3/2000 | 1/27/2001 | 3/22/2013 |  | 1/27/2001 | 5/31/2010 | 1 |
| 2459 | F | 2/26/2006 | 4/11/2006 | 3/22/2013 |  | 2/26/2006 | 5/31/2010 | 1 |
| 2461 | F | 1/1/1987 | 4/12/1999 | 3/18/2013 |  | 4/11/2002 | 5/31/2010 | 1 |
| 2462 | F | 7/21/2000 | 1/27/2001 | 2/4/2013 |  | 1/27/2001 | 5/31/2010 | 1 |
| 2463 | M | 7/14/2006 | 1/5/2007 | 3/18/2013 |  | 1/5/2007 | 5/31/2010 | 1 |
| 2477 | F | 1/1/1960 | 9/22/1992 | 5/21/2012 |  | 9/22/1995 | 5/31/2010 | 1 |
| 2479 | M | 1/1/1978 | 9/22/1992 | 1/24/2004 | 11/17/2004 | 9/22/1995 | 11/17/2004 | 1 |
| 2480 | F | 8/1/1989 | 9/22/1992 | 5/9/2009 | 4/15/2011 | 9/22/1995 | 5/31/2010 | 1 |
| 2481 | F | 1/5/1994 | 1/11/1994 | 5/21/2012 |  | 1/5/1994 | 5/31/2010 | 1 |
| 2483 | M | 10/11/1999 | 1/20/2004 | 5/18/2011 | 2/6/2012 | 1/20/2004 | 5/31/2010 | 1 |
| 2484 | M | 1/1/1980 | 2/28/1991 | 2/23/2013 |  | 2/27/1994 | 5/31/2010 | 1 |
| 2494 | F | 1/1/1973 | 1/13/1994 | 4/15/2013 |  | 1/12/1997 | 5/31/2010 | 1 |
| 2495 | M | 7/1/1994 | 7/6/1994 | 3/21/2013 |  | 7/1/1994 | 5/31/2010 | 1 |
| 2496 | F | 1/1/2001 | 4/10/2003 | 4/15/2013 |  | 4/10/2003 | 5/31/2010 | 1 |
| 2497 | M | 4/1/2006 | 12/3/2006 | 4/15/2013 |  | 12/3/2006 | 5/31/2010 | 1 |
| 2511 | M | 1/1/1973 | 10/11/1991 | 10/20/2001 | 4/10/2002 | 10/10/1994 | 4/10/2002 | 0 |
| 2512 | M | 1/1/1964 | 10/17/1990 | 5/13/2010 | 1/6/2011 | 10/16/1993 | 5/31/2010 | 1 |
| 2516 | F | 1/1/1966 | 4/26/1992 | 5/26/2012 |  | 4/26/1995 | 5/31/2010 | 1 |
| 2517 | F | 1/1/1975 | 4/26/1992 | 5/26/2012 |  | 4/26/1995 | 5/31/2010 | 1 |
| 2518 | M | 1/1/1987 | 3/1/1996 | 1/15/2013 |  | 3/1/1996 | 5/31/2010 | 1 |
| 2525 | M | 4/1/1990 | 4/26/1992 | 5/26/2012 |  | 4/26/1995 | 5/31/2010 | 1 |
| 2526 | M | 4/15/2001 | 4/18/2001 | 5/26/2012 |  | 4/15/2001 | 5/31/2010 | 1 |
| 2527 | M | 1/1/1941 | 2/22/1991 | 12/11/1998 | 8/30/1999 | 2/21/1994 | 8/30/1999 | 0 |
| 2530 | F | 1/1/1950 | 1/3/1993 | 1/13/2003 | 9/18/2003 | 1/3/1996 | 9/18/2003 | 0 |
| 2531 | F | 1/1/1989 | 1/3/1993 | 3/27/2002 | 7/20/2003 | 1/3/1996 | 7/20/2003 | 1 |
| 2532 | F | 1/1/1990 | 1/3/1993 | 1/15/2010 | 12/25/2010 | 1/3/1996 | 5/31/2010 | 1 |
| 2537 | F | 1/1/1975 | 12/1/1990 | 12/28/2012 |  | 11/30/1993 | 5/31/2010 | 1 |
| 2538 | F | 10/10/1992 | 12/20/1993 | 12/28/2012 |  | 12/20/1993 | 5/31/2010 | 1 |
| 2539 | F | 3/31/2002 | 9/13/2002 | 12/28/2012 |  | 9/13/2002 | 5/31/2010 | 1 |
| 2542 | M | 1/1/1963 | 10/17/1993 | 1/3/2000 | 12/8/2000 | 10/16/1996 | 12/8/2000 | 0 |
| 2547 | M | 1/1/1966 | 1/8/1991 | 2/6/1998 | 6/6/1998 | 1/7/1994 | 6/6/1998 | 0 |
| 2558 | M | 1/1/1969 | 6/24/1994 | 6/14/2002 | 2/23/2003 | 6/23/1997 | 2/23/2003 | 0 |
| 2562 | F | 7/7/2007 | 1/25/2008 | 3/6/2013 |  | 1/25/2008 | 5/31/2010 | 1 |
| 2564 | F | 1/1/1963 | 11/15/1992 | 7/27/2009 | 11/22/2009 | 11/15/1995 | 11/22/2009 | 0 |
| 2565 | M | 10/1/1986 | 11/15/1992 | 8/6/1997 | 2/24/1998 | 11/15/1995 | 2/24/1998 | 1 |
| 2566 | M | 10/1/1989 | 11/15/1992 | 6/14/2008 | 11/29/2008 | 11/15/1995 | 11/29/2008 | 1 |
| 2567 | F | 2/1/1997 | 5/29/1997 | 7/27/2009 | 11/30/2009 | 5/29/1997 | 11/30/2009 | 1 |
| 2568 | M | 7/1/2003 | 4/27/2004 | 7/27/2009 | 2/26/2010 | 4/27/2004 | 2/26/2010 | 1 |
| 2569 | F | 1/1/1967 | 11/5/1991 | 10/30/1995 | 2/21/1997 | 11/4/1994 | 2/21/1997 | 0 |
| 2570 | M | 11/1/1990 | 11/5/1991 | 10/21/1993 | 10/8/1994 | 11/5/1991 | 10/8/1994 | 0 |
| 2578 | F | 1/1/1975 | 1/14/1991 | 1/18/2013 |  | 1/13/1994 | 5/31/2010 | 1 |
| 2579 | M | 9/1/1999 | 11/2/2000 | 1/18/2013 |  | 11/2/2000 | 5/31/2010 | 1 |
| 2580 | F | 2/1/2007 | 7/24/2007 | 1/18/2013 |  | 7/24/2007 | 5/31/2010 | 1 |
| 2597 | M | 1/1/1951 | 8/13/1991 | 4/10/1996 | 7/5/1996 | 8/12/1994 | 7/5/1996 | 0 |
| 2598 | F | 1/1/1989 | 1/14/1991 | 1/5/2007 | 4/3/2007 | 1/13/1994 | 4/3/2007 | 0 |
| 2602 | F | 1/1/1964 | 7/8/2000 | 10/27/2010 | 6/20/2011 | 7/8/2003 | 5/31/2010 | 1 |
| 2603 | F | 4/1/1996 | 7/8/2000 | 9/13/2009 | 5/12/2011 | 7/8/2000 | 5/31/2010 | 1 |
| 2604 | F | 1/20/2002 | 5/8/2002 | 11/5/2009 | 7/3/2010 | 5/8/2002 | 5/31/2010 | 1 |
| 2605 | F | 1/16/2006 | 7/3/2006 | 10/27/2010 | 4/23/2011 | 7/3/2006 | 5/31/2010 | 1 |
| 2609 | F | 1/1/1967 | 1/20/1992 | 2/28/2013 |  | 1/19/1995 | 5/31/2010 | 1 |
| 2610 | F | 1/1/1992 | 1/20/1992 | 2/7/2013 |  | 1/1/1992 | 5/31/2010 | 1 |
| 2611 | F | 6/18/1998 | 12/20/1998 | 2/7/2013 |  | 12/20/1998 | 5/31/2010 | 1 |
| 2612 | F | 1/1/2003 | 12/29/2005 | 2/28/2013 |  | 12/29/2005 | 5/31/2010 | 1 |
| 2618 | M | 1/1/1968 | 5/9/2006 | 10/7/2012 |  | 5/8/2009 | 5/31/2010 | 1 |
| 2620 | F | 1/1/1964 | 10/27/1992 | 7/23/2002 | 11/18/2002 | 10/27/1995 | 11/18/2002 | 0 |
| 2622 | F | 1/10/1998 | 4/21/1998 | 7/23/2002 | 10/21/2002 | 4/21/1998 | 10/21/2002 | 0 |
| 2634 | F | 1/1/1963 | 1/10/1991 | 5/6/1999 | 9/9/1999 | 1/9/1994 | 9/9/1999 | 0 |
| 2635 | F | 5/1/1983 | 1/10/1991 | 2/26/1999 | 7/10/1999 | 1/9/1994 | 7/10/1999 | 1 |
| 2636 | F | 10/28/1991 | 7/14/1992 | 5/6/1999 | 8/22/1999 | 7/14/1992 | 8/22/1999 | 1 |
| 2651 | M | 1/1/1961 | 9/6/2002 | 10/4/2012 |  | 9/5/2005 | 5/31/2010 | 1 |
| 2655 | M | 1/1/1965 | 11/1/1992 | 1/18/2013 |  | 11/1/1995 | 5/31/2010 | 1 |
| 2665 | M | 1/1/1930 | 10/15/1990 | 10/20/1993 | 7/9/1994 | 10/14/1993 | 7/9/1994 | 0 |
| 2666 | F | 1/1/1966 | 10/27/1991 | 3/28/1996 | 5/29/1996 | 10/26/1994 | 5/29/1996 | 0 |
| 2667 | F | 10/1/1990 | 10/27/1991 | 12/22/1996 | 3/2/1997 | 10/27/1991 | 12/22/1996 | 1 |
| 2670 | M | 1/1/1982 | 1/15/1998 | 3/12/2013 |  | 1/14/2001 | 5/31/2010 | 1 |
| 2673 | M | 3/1/2001 | 3/5/2007 | 2/8/2013 |  | 3/5/2007 | 5/31/2010 | 1 |
| 2674 | F | 1/1/1959 | 8/8/2004 | 1/3/2011 | 8/21/2011 | 8/8/2007 | 5/31/2010 | 1 |
| 2675 | F | 1/1/1992 | 8/8/2004 | 11/15/2012 |  | 8/8/2004 | 5/31/2010 | 1 |
| 2676 | F | 1/1/1999 | 8/8/2004 | 1/3/2011 | 8/21/2011 | 8/8/2004 | 5/31/2010 | 1 |
| 2679 | M | 5/2/2006 | 8/8/2006 | 1/3/2011 | 9/24/2011 | 8/8/2006 | 5/31/2010 | 1 |
| 2681 | M | 1/1/1983 | 10/12/2005 | 11/3/2013 |  | 10/11/2008 | 5/31/2010 | 1 |
| 2713 | F | 1/1/1951 | 4/24/1992 | 3/1/2007 | 1/3/2008 | 4/24/1995 | 1/3/2008 | 0 |
| 2716 | M | 6/1/1993 | 3/15/1995 | 2/21/2004 | 12/27/2004 | 3/15/1995 | 12/27/2004 | 1 |
| 2718 | F | 2/15/1996 | 3/10/1996 | 12/30/2006 | 9/18/2007 | 2/15/1996 | 9/18/2007 | 0 |
| 2719 | F | 11/8/2002 | 12/22/2002 | 5/8/2006 | 1/14/2008 | 11/8/2002 | 1/14/2008 | 0 |
| 2723 | F | 1/1/1975 | 12/28/1998 | 1/16/2013 |  | 12/27/2001 | 5/31/2010 | 1 |
| 2724 | F | 1/1/1994 | 12/28/1998 | 1/15/2013 |  | 12/28/1998 | 5/31/2010 | 1 |
| 2725 | F | 5/1/2002 | 6/17/2002 | 10/26/2012 |  | 5/1/2002 | 5/31/2010 | 1 |
| 2729 | M | 5/1/2007 | 11/5/2007 | 1/16/2013 |  | 11/5/2007 | 5/31/2010 | 1 |
| 2732 | F | 1/1/1964 | 8/20/1992 | 3/21/2013 |  | 8/20/1995 | 5/31/2010 | 1 |
| 2733 | F | 8/1/1992 | 8/20/1992 | 3/11/2013 |  | 8/1/1992 | 5/31/2010 | 1 |
| 2734 | F | 3/1/1997 | 7/26/1997 | 9/29/2012 |  | 7/26/1997 | 5/31/2010 | 1 |
| 2736 | M | 7/29/2002 | 9/16/2002 | 9/27/2012 |  | 7/29/2002 | 5/31/2010 | 1 |
| 2737 | M | 2/15/2007 | 3/22/2007 | 3/21/2013 |  | 2/15/2007 | 5/31/2010 | 1 |
| 2764 | F | 1/1/1971 | 12/18/2001 | 1/28/2012 |  | 12/17/2004 | 5/31/2010 | 1 |
| 2765 | M | 1/1/1990 | 3/20/2002 | 12/22/2011 |  | 12/17/2004 | 5/31/2010 | 1 |
| 2766 | M | 1/1/1995 | 12/18/2001 | 1/28/2012 |  | 12/18/2001 | 5/31/2010 | 1 |
| 2767 | M | 12/1/2000 | 12/18/2001 | 1/28/2012 |  | 12/18/2001 | 5/31/2010 | 1 |
| 2774 | F | 1/1/1956 | 12/31/1990 | 1/27/1999 | 6/2/1999 | 12/30/1993 | 6/2/1999 | 0 |
| 2775 | M | 1/1/1977 | 12/31/1990 | 7/18/2005 | 11/13/2005 | 12/30/1993 | 11/13/2005 | 0 |
| 2777 | F | 5/20/1995 | 7/29/1995 | 1/27/1999 | 5/29/1999 | 7/29/1995 | 5/29/1999 | 0 |
| 2781 | F | 9/4/2003 | 5/9/2006 | 1/18/2013 |  | 5/9/2006 | 5/31/2010 | 1 |
| 2791 | F | 1/1/1960 | 10/11/1991 | 1/27/2013 |  | 10/10/1994 | 5/31/2010 | 1 |
| 2792 | F | 1/1/1994 | 12/14/2000 | 10/11/2012 |  | 12/14/2000 | 5/31/2010 | 1 |
| 2793 | F | 5/1/2009 | 5/1/2009 | 10/11/2012 |  | 5/1/2009 | 5/31/2010 | 1 |
| 2794 | M | 1/1/1988 | 10/11/1991 | 8/13/2003 | 11/7/2003 | 10/10/1994 | 11/7/2003 | 0 |
| 2795 | M | 9/30/1993 | 10/18/1993 | 11/3/2006 | 1/18/2007 | 9/30/1993 | 1/18/2007 | 0 |
| 2796 | M | 3/1/1999 | 5/28/1999 | 3/9/2010 | 6/5/2010 | 5/28/1999 | 5/31/2010 | 1 |
| 2797 | F | 4/1/2003 | 9/8/2003 | 1/27/2013 |  | 9/8/2003 | 5/31/2010 | 1 |
| 2798 | F | 5/15/2007 | 7/2/2007 | 1/27/2013 |  | 5/15/2007 | 5/31/2010 | 1 |
| 2811 | F | 1/1/1948 | 2/11/1993 | 1/2/2003 | 7/21/2003 | 2/11/1996 | 7/21/2003 | 0 |
| 2812 | F | 1/1/1975 | 2/11/1993 | 10/16/2004 | 6/6/2006 | 2/11/1996 | 6/6/2006 | 0 |
| 2813 | M | 1/1/1990 | 2/11/1993 | 2/5/2001 | 8/12/2001 | 2/11/1996 | 8/12/2001 | 1 |
| 2817 | F | 10/30/2000 | 1/26/2001 | 1/2/2003 | 9/29/2003 | 1/26/2001 | 9/29/2003 | 0 |
| 2818 | F | 7/28/2001 | 1/12/2002 | 1/2/2003 | 6/21/2003 | 1/12/2002 | 6/21/2003 | 0 |
| 2819 | F | 1/1/1970 | 6/10/1991 | 3/6/1998 | 10/25/2010 | 6/9/1994 | 5/31/2010 | 1 |
| 2820 | M | 1/1/1984 | 6/10/1991 | 2/19/2011 | 10/13/2011 | 6/9/1994 | 5/31/2010 | 1 |
| 2821 | F | 1/1/1991 | 7/17/1992 | 12/3/2012 |  | 7/17/1992 | 5/31/2010 | 1 |
| 2824 | F | 3/16/1997 | 5/18/1997 | 12/20/1998 | 5/29/1999 | 5/18/1997 | 5/29/1999 | 0 |
| 2830 | M | 1/1/1961 | 9/19/1991 | 11/12/1995 | 4/2/1996 | 9/18/1994 | 4/2/1996 | 0 |
| 2877 | F | 1/1/1992 | 8/23/1998 | 12/23/2011 |  | 8/23/1998 | 5/31/2010 | 1 |
| 2899 | M | 1/1/1959 | 2/1/1992 | 12/30/2012 |  | 1/31/1995 | 5/31/2010 | 1 |
| 2909 | F | 1/1/1958 | 2/1/1993 | 4/15/2013 |  | 2/1/1996 | 5/31/2010 | 1 |
| 2911 | F | 1/1/1981 | 3/15/1993 | 4/15/2013 |  | 2/1/1996 | 5/31/2010 | 1 |
| 2912 | M | 1/1/1980 | 2/1/1993 | 4/8/2001 | 9/29/2001 | 2/1/1996 | 9/29/2001 | 1 |
| 2916 | M | 6/25/1999 | 10/25/1999 | 10/25/2008 | 7/26/2009 | 10/25/1999 | 7/26/2009 | 1 |
| 2917 | M | 4/5/2003 | 4/11/2003 | 4/15/2013 |  | 4/5/2003 | 5/31/2010 | 1 |
| 2921 | F | 1/1/1938 | 5/4/1993 | 4/9/1998 | 8/21/1998 | 5/3/1996 | 8/21/1998 | 0 |
| 2935 | M | 1/1/1946 | 1/4/1991 | 8/19/1999 | 2/1/2000 | 1/3/1994 | 2/1/2000 | 0 |
| 2944 | M | 1/1/1957 | 12/31/1992 | 2/13/2013 |  | 12/31/1995 | 5/31/2010 | 1 |
| 2950 | M | 1/1/1970 | 8/21/1991 | 1/21/2010 | 7/18/2010 | 8/20/1994 | 5/31/2010 | 1 |
| 2952 | F | 1/1/1960 | 4/12/1993 | 1/27/2013 |  | 4/11/1996 | 5/31/2010 | 1 |
| 2953 | F | 1/1/1981 | 4/12/1993 | 12/29/2012 |  | 4/11/1996 | 5/31/2010 | 1 |
| 2954 | F | 4/12/1993 | 4/12/1993 | 1/19/2013 |  | 4/12/1993 | 5/31/2010 | 1 |
| 2961 | F | 8/1/2001 | 10/17/2001 | 11/9/2012 |  | 10/17/2001 | 5/31/2010 | 1 |
| 2990 | M | 1/1/1967 | 3/22/1992 | 1/13/2002 | 11/29/2002 | 3/22/1995 | 11/29/2002 | 0 |
| 2991 | F | 1/1/1991 | 7/1/1998 | 4/15/2013 |  | 7/1/1998 | 5/31/2010 | 1 |
| 2993 | F | 12/2/2005 | 5/8/2006 | 5/18/2006 | 5/19/2006 | 5/8/2006 | 5/19/2006 | 0 |
| 2998 | M | 1/1/1979 | 9/28/1991 | 8/25/1999 | 2/15/2000 | 9/27/1994 | 2/15/2000 | 0 |
| 3000 | F | 1/1/1985 | 1/14/1991 | 2/24/2013 |  | 1/13/1994 | 5/31/2010 | 1 |
| 3006 | M | 1/1/1964 | 10/4/1991 | 3/4/2010 | 1/12/2011 | 10/3/1994 | 5/31/2010 | 1 |
| 3012 | M | 1/1/1981 | 1/23/1991 | 2/10/2013 |  | 1/22/1994 | 5/31/2010 | 1 |
| 3013 | M | 1/1/1950 | 11/9/1990 | 10/11/1996 | 4/15/1997 | 11/8/1993 | 4/15/1997 | 0 |
| 3015 | F | 1/1/1941 | 1/14/1991 | 6/5/1999 | 2/22/2000 | 1/13/1994 | 2/22/2000 | 0 |
| 3017 | M | 10/13/2001 | 10/17/2001 | 12/15/2011 |  | 10/13/2001 | 5/31/2010 | 1 |
| 3026 | F | 1/1/1973 | 8/31/1992 | 2/23/2013 |  | 8/31/1995 | 5/31/2010 | 1 |
| 3028 | M | 1/1/1985 | 8/31/1992 | 3/26/2012 |  | 8/31/1995 | 5/31/2010 | 1 |
| 3029 | F | 1/1/1989 | 8/31/1992 | 1/18/2013 |  | 8/31/1995 | 5/31/2010 | 1 |
| 3034 | F | 6/1/1997 | 8/3/1997 | 11/4/2012 |  | 8/3/1997 | 5/31/2010 | 1 |
| 3035 | M | 8/1/2002 | 12/6/2002 | 2/3/2011 | 7/7/2011 | 12/6/2002 | 5/31/2010 | 1 |
| 3036 | M | 2/1/2007 | 3/10/2007 | 11/11/2012 |  | 2/1/2007 | 5/31/2010 | 1 |
| 3045 | F | 1/1/1994 | 7/31/2007 | 4/15/2013 |  | 7/31/2007 | 5/31/2010 | 1 |
| 3046 | M | 6/3/2008 | 10/15/2008 | 4/15/2013 |  | 10/15/2008 | 5/31/2010 | 1 |
| 3048 | F | 1/1/1970 | 8/31/1992 | 3/22/2013 |  | 8/31/1995 | 5/31/2010 | 1 |
| 3053 | F | 1/1/1999 | 5/11/1999 | 11/21/2002 | 6/3/2006 | 5/11/1999 | 6/3/2006 | 0 |
| 3054 | F | 1/1/1987 | 8/31/1992 | 3/22/2013 |  | 8/31/1995 | 5/31/2010 | 1 |
| 3055 | F | 6/1/1991 | 8/31/1992 | 3/22/2013 |  | 8/31/1992 | 5/31/2010 | 1 |
| 3058 | F | 8/1/1999 | 3/26/2000 | 3/22/2013 |  | 3/26/2000 | 5/31/2010 | 1 |
| 3059 | F | 8/15/2001 | 10/5/2001 | 3/22/2013 |  | 8/15/2001 | 5/31/2010 | 1 |
| 3061 | M | 1/1/1984 | 12/22/2001 | 3/13/2013 |  | 12/21/2004 | 5/31/2010 | 1 |
| 3069 | F | 1/1/1938 | 5/9/1993 | 5/12/2004 | 12/10/2004 | 5/8/1996 | 12/10/2004 | 0 |
| 3071 | F | 5/1/1992 | 5/9/1993 | 5/6/2012 |  | 5/9/1993 | 5/31/2010 | 1 |
| 3074 | F | 1/1/1979 | 4/30/1994 | 4/17/1999 | 9/22/1999 | 4/29/1997 | 9/22/1999 | 0 |
| 3077 | M | 1/1/1951 | 9/27/1991 | 8/2/1995 | 6/5/1996 | 9/26/1994 | 6/5/1996 | 0 |
| 3080 | F | 1/1/1963 | 6/4/1991 | 6/2/2006 | 12/23/2006 | 6/3/1994 | 12/23/2006 | 0 |
| 3081 | F | 1/1/1989 | 6/4/1991 | 4/7/2004 | 5/25/2006 | 6/3/1994 | 5/25/2006 | 1 |
| 3082 | F | 12/26/1993 | 3/3/1994 | 6/2/2006 | 1/6/2007 | 3/3/1994 | 1/6/2007 | 1 |
| 3083 | M | 1/12/2000 | 6/28/2000 | 6/2/2006 | 5/22/2007 | 6/28/2000 | 5/22/2007 | 1 |
| 3093 | F | 1/1/1958 | 4/5/1993 | 1/6/2013 |  | 4/4/1996 | 5/31/2010 | 1 |
| 3095 | F | 1/1/1976 | 4/6/2002 | 3/21/2013 |  | 4/5/2005 | 5/31/2010 | 1 |
| 3099 | F | 1/1/1997 | 4/6/2002 | 9/18/2011 |  | 4/6/2002 | 5/31/2010 | 1 |
| 3100 | M | 1/1/2000 | 4/6/2002 | 3/24/2010 |  | 4/6/2002 | 3/24/2010 | 1 |
| 3103 | F | 6/10/2006 | 6/11/2006 | 3/21/2013 |  | 6/10/2006 | 5/31/2010 | 1 |
| 3107 | M | 1/1/1985 | 1/1/1995 | 3/13/2013 |  | 4/4/1996 | 5/31/2010 | 1 |
| 3108 | M | 4/1/1989 | 4/5/1993 | 2/20/2012 |  | 4/4/1996 | 5/31/2010 | 1 |
| 3111 | F | 12/20/1999 | 4/16/2000 | 1/6/2013 |  | 4/16/2000 | 5/31/2010 | 1 |
| 3112 | M | 7/29/2004 | 9/17/2005 | 2/24/2012 |  | 9/17/2005 | 5/31/2010 | 1 |
| 3145 | F | 1/1/1998 | 7/27/2007 | 1/19/2013 |  | 7/27/2007 | 5/31/2010 | 1 |
| 3147 | M | 1/1/1965 | 11/4/1990 | 4/19/1998 | 8/9/1998 | 11/3/1993 | 8/9/1998 | 0 |
| 3155 | F | 1/1/1962 | 8/8/1997 | 7/23/2002 | 3/20/2003 | 8/7/2000 | 3/20/2003 | 0 |
| 3156 | M | 8/1/1991 | 8/8/1997 | 8/11/1999 | 1/18/2000 | 8/8/1997 | 1/18/2000 | 0 |
| 3157 | F | 12/28/1997 | 1/9/1998 | 7/23/2002 | 7/26/2003 | 12/28/1997 | 7/26/2003 | 0 |
| 3191 | M | 1/1/1953 | 11/4/1990 | 4/17/2008 | 7/28/2008 | 11/3/1993 | 7/28/2008 | 0 |
| 3199 | F | 1/1/1968 | 5/14/1998 | 4/15/2013 |  | 5/13/2001 | 5/31/2010 | 1 |
| 3201 | F | 1/1/1986 | 5/14/1998 | 5/18/2005 | 4/29/2006 | 5/13/2001 | 5/18/2005 | 1 |
| 3202 | F | 1/1/1980 | 5/26/1998 | 1/19/2013 |  | 5/13/2001 | 5/31/2010 | 1 |
| 3209 | F | 9/1/1999 | 7/20/2001 | 5/22/2012 |  | 7/20/2001 | 5/31/2010 | 1 |
| 3210 | M | 6/22/2005 | 7/29/2005 | 4/15/2013 |  | 6/22/2005 | 5/31/2010 | 1 |
| 3217 | M | 1/1/1945 | 12/1/1990 | 2/28/1998 | 10/10/1998 | 11/30/1993 | 10/10/1998 | 0 |
| 3220 | M | 1/1/1945 | 11/8/1990 | 10/7/1997 | 11/27/1998 | 11/7/1993 | 11/27/1998 | 0 |
| 3224 | M | 1/1/1956 | 2/1/1991 | 2/18/2004 | 12/6/2004 | 1/31/1994 | 12/6/2004 | 0 |
| 3230 | F | 1/1/1937 | 9/23/1992 | 11/25/1998 | 7/7/1999 | 9/23/1995 | 7/7/1999 | 0 |
| 3231 | F | 9/1/1980 | 9/23/1992 | 9/6/1998 | 6/21/1999 | 9/23/1995 | 6/21/1999 | 0 |
| 3237 | F | 1/1/1946 | 2/25/1992 | 8/22/2006 | 10/25/2006 | 2/24/1995 | 10/25/2006 | 0 |
| 3239 | F | 1/1/1982 | 2/25/1992 | 12/1/2012 |  | 2/24/1995 | 5/31/2010 | 1 |
| 3240 | F | 2/1/1991 | 2/25/1992 | 6/4/2003 | 8/23/2003 | 2/25/1992 | 8/23/2003 | 0 |
| 3246 | F | 6/24/1996 | 10/3/1996 | 5/17/2006 | 8/13/2006 | 10/3/1996 | 8/13/2006 | 0 |
| 3247 | F | 4/1/2002 | 9/6/2002 | 8/22/2006 | 10/31/2006 | 9/6/2002 | 10/31/2006 | 0 |
| 3252 | F | 1/1/1976 | 7/21/1992 | 3/22/2013 |  | 7/21/1995 | 5/31/2010 | 1 |
| 3253 | M | 5/1/2004 | 9/20/2005 | 11/29/2011 |  | 9/20/2005 | 5/31/2010 | 1 |
| 3255 | M | 1/1/1946 | 1/3/1991 | 3/17/1995 | 10/7/1995 | 1/2/1994 | 10/7/1995 | 0 |
| 3256 | M | 1/1/1953 | 10/19/1990 | 10/3/2007 | 6/17/2008 | 10/18/1993 | 6/17/2008 | 0 |
| 3281 | M | 1/1/1997 | 3/3/2007 | 3/6/2010 | 12/17/2010 | 3/3/2007 | 5/31/2010 | 1 |
| 3283 | F | 1/1/1959 | 7/10/1991 | 3/30/2003 | 6/20/2003 | 7/9/1994 | 6/20/2003 | 0 |
| 3284 | F | 7/1/1988 | 7/10/1991 | 3/30/2003 | 10/20/2003 | 7/9/1994 | 10/20/2003 | 1 |
| 3285 | M | 1/1/1993 | 1/3/1993 | 3/30/2003 | 6/10/2003 | 1/1/1993 | 6/10/2003 | 1 |
| 3286 | M | 2/21/2001 | 4/8/2001 | 2/22/2002 | 3/4/2002 | 2/21/2001 | 3/4/2002 | 0 |
| 3292 | M | 1/1/1970 | 9/10/2005 | 1/28/2013 |  | 9/9/2008 | 5/31/2010 | 1 |
| 3295 | F | 1/1/1985 | 3/30/1991 | 3/24/2000 | 9/30/2000 | 3/29/1994 | 9/30/2000 | 0 |
| 3302 | M | 1/1/1969 | 3/2/1991 | 1/17/2004 | 9/23/2004 | 3/1/1994 | 9/23/2004 | 0 |
| 3303 | M | 1/1/1956 | 1/1/1991 | 3/7/1994 | 7/3/1994 | 12/31/1993 | 7/3/1994 | 0 |
| 3322 | F | 1/1/1952 | 9/4/1992 | 7/13/2001 | 10/10/2002 | 9/4/1995 | 10/10/2002 | 0 |
| 3323 | F | 9/1/1986 | 9/6/1993 | 8/25/1999 | 10/24/2000 | 9/4/1995 | 10/24/2000 | 1 |
| 3324 | F | 9/1/1990 | 9/4/1992 | 7/13/2001 | 10/10/2002 | 9/4/1992 | 10/10/2002 | 1 |
| 3325 | M | 5/6/1994 | 11/22/1994 | 7/13/2001 | 11/1/2002 | 11/22/1994 | 11/1/2002 | 1 |
| 3329 | F | 1/1/1959 | 2/20/1992 | 10/26/2012 |  | 2/19/1995 | 5/31/2010 | 1 |
| 3331 | F | 7/1/2001 | 11/24/2001 | 4/22/2008 | 10/5/2008 | 11/24/2001 | 10/5/2008 | 1 |
| 3334 | M | 9/25/2006 | 10/2/2006 | 3/21/2010 | 8/8/2010 | 9/25/2006 | 5/31/2010 | 0 |
| 3337 | F | 1/1/1961 | 8/9/1991 | 9/23/2012 |  | 8/8/1994 | 5/31/2010 | 1 |
| 3338 | M | 1/1/1976 | 8/9/1991 | 7/27/2000 | 3/26/2001 | 8/8/1994 | 3/26/2001 | 1 |
| 3339 | M | 1/1/1988 | 8/9/1991 | 7/24/2007 | 6/12/2008 | 8/8/1994 | 7/24/2007 | 1 |
| 3340 | F | 5/4/1991 | 10/15/1991 | 9/18/2010 | 7/29/2011 | 10/15/1991 | 5/31/2010 | 1 |
| 3341 | M | 5/1/2006 | 7/26/2006 | 9/18/2010 |  | 7/26/2006 | 5/31/2010 | 1 |
| 3343 | M | 3/12/1995 | 9/15/1995 | 10/9/2011 |  | 9/15/1995 | 5/31/2010 | 1 |
| 3344 | F | 1/1/1999 | 7/29/1999 | 9/23/2012 |  | 7/29/1999 | 5/31/2010 | 1 |
| 3345 | M | 1/1/2002 | 9/11/2003 | 9/23/2012 |  | 9/11/2003 | 5/31/2010 | 1 |
| 3346 | F | 1/1/1960 | 8/15/1992 | 9/17/2012 |  | 8/15/1995 | 5/31/2010 | 1 |
| 3347 | F | 1/1/1980 | 8/15/1992 | 6/22/2008 | 6/17/2009 | 8/15/1995 | 6/17/2009 | 0 |
| 3348 | F | 11/2/1998 | 4/23/1999 | 6/22/2008 |  | 4/23/1999 | 5/31/2010 | 1 |
| 3349 | M | 1/1/1991 | 8/15/1992 | 10/9/2011 |  | 8/15/1992 | 5/31/2010 | 1 |
| 3350 | M | 1/20/2000 | 7/21/2000 | 10/26/2002 | 5/20/2003 | 7/21/2000 | 5/20/2003 | 0 |
| 3351 | F | 6/10/2005 | 7/15/2006 | 10/19/2011 |  | 7/15/2006 | 5/31/2010 | 1 |
| 3356 | M | 1/1/1961 | 2/10/1991 | 1/2/2006 | 8/10/2006 | 2/9/1994 | 8/10/2006 | 0 |
| 3365 | M | 1/1/1980 | 10/2/1992 | 5/2/1997 | 11/10/1997 | 10/2/1995 | 11/10/1997 | 0 |
| 3366 | F | 1/1/1987 | 6/9/2002 | 3/22/2012 |  | 6/8/2005 | 5/31/2010 | 1 |
| 3371 | F | 4/1/2006 | 5/7/2006 | 3/22/2012 |  | 4/1/2006 | 5/31/2010 | 1 |
| 3372 | F | 1/1/1961 | 6/10/1991 | 1/21/2013 |  | 6/9/1994 | 5/31/2010 | 1 |
| 3373 | F | 1/1/1980 | 6/10/1991 | 1/21/2013 |  | 6/9/1994 | 5/31/2010 | 1 |
| 3374 | F | 1/1/1985 | 6/10/1991 | 3/8/2013 |  | 6/9/1994 | 5/31/2010 | 1 |
| 3382 | F | 10/10/1994 | 12/6/1994 | 12/23/2011 | 5/3/2012 | 10/10/1994 | 5/31/2010 | 1 |
| 3383 | M | 4/1/1999 | 7/25/1999 | 3/28/2008 | 9/28/2008 | 7/25/1999 | 9/28/2008 | 1 |
| 3384 | F | 3/31/2002 | 6/27/2002 | 1/10/2013 |  | 6/27/2002 | 5/31/2010 | 1 |
| 3385 | F | 9/1/2006 | 10/11/2006 | 1/21/2013 |  | 9/1/2006 | 5/31/2010 | 1 |
| 3387 | F | 1/1/1974 | 5/30/1995 | 1/3/2013 |  | 5/29/1998 | 5/31/2010 | 1 |
| 3388 | F | 1/1/1968 | 8/15/2003 | 11/2/2012 |  | 8/14/2006 | 5/31/2010 | 1 |
| 3389 | F | 1/1/1994 | 8/15/2003 | 11/2/2012 |  | 8/15/2003 | 5/31/2010 | 1 |
| 3390 | F | 9/9/2004 | 10/9/2004 | 11/2/2012 |  | 9/9/2004 | 5/31/2010 | 1 |
| 3391 | M | 1/1/2007 | 11/13/2007 | 11/2/2012 |  | 11/13/2007 | 5/31/2010 | 1 |
| 3392 | F | 5/1/1993 | 5/30/1995 | 6/11/2008 | 11/16/2008 | 5/30/1995 | 11/16/2008 | 1 |
| 3393 | M | 5/18/1997 | 9/15/1997 | 12/1/2010 | 6/9/2011 | 9/15/1997 | 5/31/2010 | 1 |
| 3394 | M | 4/1/2002 | 10/16/2002 | 2/24/2013 |  | 10/16/2002 | 5/31/2010 | 1 |
| 3395 | F | 1/1/1966 | 9/20/1991 | 4/14/2009 | 11/14/2009 | 9/19/1994 | 11/14/2009 | 0 |
| 3396 | M | 1/1/1985 | 9/20/1991 | 12/18/1998 | 10/18/1999 | 9/19/1994 | 10/18/1999 | 1 |
| 3398 | F | 11/1/1994 | 11/8/1994 | 11/16/2012 |  | 11/1/1994 | 5/31/2010 | 1 |
| 3400 | M | 1/1/2001 | 10/28/2006 | 4/15/2013 |  | 10/28/2006 | 5/31/2010 | 1 |
| 3406 | M | 1/1/1980 | 8/8/1992 | 11/17/2000 | 6/7/2001 | 8/8/1995 | 6/7/2001 | 0 |
| 3410 | F | 1/1/1965 | 1/11/1991 | 1/18/2013 |  | 1/10/1994 | 5/31/2010 | 1 |
| 3412 | M | 1/1/1988 | 1/11/1991 | 1/28/2013 |  | 1/10/1994 | 5/31/2010 | 1 |
| 3413 | M | 2/9/1992 | 4/15/1992 | 4/13/2007 | 9/2/2007 | 4/15/1992 | 9/2/2007 | 1 |
| 3414 | F | 4/8/1997 | 4/10/1997 | 1/18/2013 |  | 4/8/1997 | 5/31/2010 | 1 |
| 3415 | F | 7/17/2003 | 10/8/2003 | 1/18/2013 |  | 10/8/2003 | 5/31/2010 | 1 |
| 3416 | M | 1/15/2009 | 2/27/2009 | 11/15/2012 |  | 1/15/2009 | 5/31/2010 | 1 |
| 3417 | M | 1/1/1971 | 3/19/1991 | 5/8/1999 | 2/2/2000 | 3/18/1994 | 2/2/2000 | 0 |
| 3423 | F | 1/1/1985 | 1/13/1991 | 1/3/2013 |  | 1/12/1994 | 5/31/2010 | 1 |
| 3424 | F | 11/1/1996 | 12/22/1996 | 1/3/2013 |  | 11/1/1996 | 5/31/2010 | 1 |
| 3427 | F | 1/1/1970 | 3/19/1991 | 2/23/2013 |  | 3/18/1994 | 5/31/2010 | 1 |
| 3428 | F | 1/1/1985 | 3/19/1991 | 3/19/2013 |  | 3/18/1994 | 5/31/2010 | 1 |
| 3429 | M | 7/1/1993 | 1/21/1994 | 7/5/2007 | 9/29/2007 | 1/21/1994 | 9/29/2007 | 0 |
| 3436 | F | 7/19/1999 | 8/14/1999 | 11/28/2012 |  | 7/19/1999 | 5/31/2010 | 1 |
| 3437 | M | 5/20/2007 | 7/18/2007 | 2/23/2013 |  | 5/20/2007 | 5/31/2010 | 1 |
| 3439 | F | 1/1/1965 | 2/20/1992 | 10/10/2011 | 6/4/2012 | 2/19/1995 | 5/31/2010 | 1 |
| 3440 | F | 1/1/1986 | 2/20/1992 | 1/13/2013 |  | 2/19/1995 | 5/31/2010 | 1 |
| 3441 | F | 4/1/2000 | 7/15/2001 | 1/13/2013 |  | 7/15/2001 | 5/31/2010 | 1 |
| 3442 | M | 3/7/2007 | 7/13/2007 | 1/13/2013 |  | 7/13/2007 | 5/31/2010 | 1 |
| 3443 | F | 1/1/1989 | 2/20/1992 | 1/7/2008 | 10/27/2008 | 2/19/1995 | 10/27/2008 | 1 |
| 3446 | M | 11/2/1996 | 1/21/1997 | 4/25/2009 | 4/14/2010 | 1/21/1997 | 4/14/2010 | 1 |
| 3447 | F | 5/14/2004 | 9/14/2004 | 10/10/2011 | 6/30/2012 | 9/14/2004 | 5/31/2010 | 1 |
| 3463 | F | 1/1/1980 | 1/3/1999 | 1/18/2013 |  | 1/2/2002 | 5/31/2010 | 1 |
| 3464 | F | 11/25/1999 | 5/7/2000 | 1/18/2013 |  | 5/7/2000 | 5/31/2010 | 1 |
| 3465 | M | 3/4/2005 | 1/16/2006 | 1/15/2013 |  | 1/16/2006 | 5/31/2010 | 1 |
| 3467 | M | 1/1/1964 | 5/7/1994 | 1/27/2013 |  | 5/6/1997 | 5/31/2010 | 1 |
| 3470 | M | 1/1/1955 | 2/6/1991 | 2/4/2013 |  | 2/5/1994 | 5/31/2010 | 1 |
| 3472 | F | 11/1/1988 | 2/20/1992 | 12/27/2010 | 12/14/2011 | 2/19/1995 | 5/31/2010 | 1 |
| 3482 | M | 1/1/1964 | 11/27/1994 | 6/30/2002 | 1/24/2003 | 11/26/1997 | 1/24/2003 | 0 |
| 3497 | M | 1/1/1981 | 2/15/1994 | 4/10/2008 | 10/25/2008 | 2/14/1997 | 10/25/2008 | 0 |
| 3498 | F | 1/1/1970 | 9/27/1991 | 1/14/1997 | 6/25/1997 | 9/26/1994 | 6/25/1997 | 0 |
| 3500 | M | 3/1/1989 | 9/27/1991 | 3/21/1995 | 7/7/1995 | 9/26/1994 | 7/7/1995 | 1 |
| 3501 | F | 7/30/1994 | 8/1/1994 | 1/14/1997 | 11/4/1997 | 7/30/1994 | 11/4/1997 | 0 |
| 3503 | M | 1/1/1982 | 3/15/2004 | 3/1/2012 |  | 3/15/2007 | 5/31/2010 | 1 |
| 3504 | M | 1/1/1974 | 8/8/1992 | 8/27/1996 | 12/29/1996 | 8/8/1995 | 12/29/1996 | 0 |
| 3506 | F | 1/1/1970 | 5/8/1993 | 1/15/2010 | 10/22/2010 | 5/7/1996 | 5/31/2010 | 1 |
| 3507 | M | 1/1/1990 | 5/8/1993 | 1/29/2013 |  | 5/7/1996 | 5/31/2010 | 1 |
| 3508 | F | 4/29/1997 | 5/3/1997 | 1/15/2010 | 11/5/2010 | 4/29/1997 | 5/31/2010 | 1 |
| 3509 | F | 4/6/1999 | 8/18/1999 | 1/15/2010 | 11/23/2010 | 8/18/1999 | 5/31/2010 | 1 |
| 3510 | M | 1/3/2005 | 5/5/2005 | 1/15/2010 | 5/18/2011 | 5/5/2005 | 5/31/2010 | 1 |
| 3511 | M | 5/8/2006 | 9/9/2006 | 1/13/2010 | 12/23/2010 | 9/9/2006 | 5/31/2010 | 0 |
| 3516 | F | 1/1/1954 | 12/28/1990 | 12/7/2005 | 7/7/2006 | 12/27/1993 | 7/7/2006 | 0 |
| 3517 | M | 1/1/1987 | 12/28/1990 | 11/18/2005 | 5/10/2007 | 12/27/1993 | 5/10/2007 | 1 |
| 3518 | M | 12/31/1995 | 8/9/1996 | 12/7/2005 | 6/25/2006 | 8/9/1996 | 6/25/2006 | 1 |
| 3530 | M | 1/1/1953 | 8/26/1993 | 12/8/2002 | 3/26/2003 | 8/25/1996 | 3/26/2003 | 0 |
| 3537 | F | 1/1/1975 | 4/12/1992 | 5/9/1996 | 10/2/1996 | 4/12/1995 | 10/2/1996 | 0 |
| 3538 | F | 4/1/1980 | 4/12/1992 | 5/9/1996 | 10/12/1996 | 4/12/1995 | 10/12/1996 | 1 |
| 3539 | M | 4/1/1988 | 4/12/1992 | 5/9/1996 | 10/4/1996 | 4/12/1995 | 10/4/1996 | 1 |
| 3540 | F | 11/1/1995 | 3/16/1996 | 5/9/1996 | 5/25/1996 | 3/16/1996 | 5/25/1996 | 0 |
| 3549 | M | 1/1/1945 | 11/1/1990 | 1/7/2001 | 10/10/2001 | 10/31/1993 | 10/10/2001 | 0 |
| 3550 | F | 1/1/1964 | 6/28/1991 | 1/7/2013 |  | 6/27/1994 | 5/31/2010 | 1 |
| 3552 | F | 1/1/1979 | 6/28/1991 | 1/7/2013 |  | 6/27/1994 | 5/31/2010 | 1 |
| 3553 | F | 1/1/1983 | 6/28/1991 | 1/6/2013 |  | 6/27/1994 | 5/31/2010 | 1 |
| 3566 | M | 7/3/1994 | 11/13/1994 | 12/21/2004 | 9/5/2005 | 11/13/1994 | 9/5/2005 | 0 |
| 3567 | F | 5/4/1999 | 7/17/1999 | 1/7/2013 |  | 7/17/1999 | 5/31/2010 | 1 |
| 3568 | M | 5/1/2003 | 8/27/2003 | 1/7/2013 |  | 8/27/2003 | 5/31/2010 | 1 |
| 3575 | F | 1/1/1959 | 9/9/1993 | 4/18/2006 | 11/24/2006 | 9/8/1996 | 11/24/2006 | 0 |
| 3576 | F | 1/1/1976 | 9/9/1993 | 10/23/2007 | 6/7/2008 | 9/8/1996 | 6/7/2008 | 0 |
| 3577 | F | 6/1/1993 | 9/9/1993 | 3/17/2013 |  | 9/9/1993 | 5/31/2010 | 1 |
| 3582 | F | 7/1/1997 | 10/2/1997 | 10/16/2007 | 7/16/2008 | 10/2/1997 | 7/16/2008 | 0 |
| 3583 | M | 8/23/2005 | 8/23/2005 | 4/18/2006 | 9/23/2006 | 8/23/2005 | 9/23/2006 | 0 |
| 3586 | M | 1/1/1969 | 2/6/1994 | 12/6/1998 | 7/26/1999 | 2/5/1997 | 7/26/1999 | 0 |
| 3588 | M | 1/1/1962 | 12/12/1997 | 10/17/2001 | 4/3/2002 | 12/11/2000 | 4/3/2002 | 0 |
| 3590 | F | 1/1/1970 | 1/8/1995 | 6/8/2006 | 2/19/2007 | 1/7/1998 | 2/19/2007 | 0 |
| 3591 | F | 1/1/1983 | 1/8/1995 | 6/8/2006 | 3/9/2007 | 1/7/1998 | 3/9/2007 | 0 |
| 3592 | M | 1/1/1985 | 1/8/1995 | 5/13/2001 | 7/5/2002 | 1/7/1998 | 7/5/2002 | 1 |
| 3596 | M | 1/1/1993 | 1/8/1995 | 11/20/2005 | 1/8/2007 | 1/8/1995 | 1/8/2007 | 1 |
| 3597 | M | 10/26/2001 | 4/10/2002 | 6/8/2006 | 12/11/2006 | 4/10/2002 | 12/11/2006 | 0 |
| 3598 | F | 1/1/1972 | 12/2/1992 | 12/14/2000 | 6/6/2001 | 12/2/1995 | 6/6/2001 | 0 |
| 3599 | F | 12/1/1989 | 12/13/1992 | 11/25/1999 | 12/15/2000 | 12/2/1995 | 12/15/2000 | 1 |
| 3600 | F | 4/5/1994 | 5/6/1994 | 12/14/2000 | 7/2/2001 | 4/5/1994 | 7/2/2001 | 1 |
| 3603 | M | 1/1/1954 | 10/18/1990 | 1/9/2008 | 10/31/2008 | 10/17/1993 | 10/31/2008 | 0 |
| 3608 | F | 1/1/1973 | 8/31/1992 | 12/20/2010 | 4/17/2011 | 8/31/1995 | 5/31/2010 | 1 |
| 3610 | M | 8/12/1993 | 11/24/1993 | 2/1/2012 | 5/11/2012 | 11/24/1993 | 5/31/2010 | 1 |
| 3611 | M | 1/1/2000 | 3/8/2000 | 11/14/2012 |  | 3/8/2000 | 5/31/2010 | 1 |
| 3612 | F | 2/20/2006 | 5/15/2006 | 4/20/2009 | 1/7/2010 | 5/15/2006 | 1/7/2010 | 0 |
| 3613 | M | 1/1/1977 | 1/27/1991 | 7/14/1998 | 3/23/1999 | 1/28/1994 | 3/23/1999 | 0 |
| 3614 | M | 1/1/1969 | 8/2/1994 | 7/15/2001 | 9/8/2002 | 8/1/1997 | 9/8/2002 | 0 |
| 3616 | M | 1/1/1977 | 4/12/1992 | 9/12/1998 | 12/20/1999 | 4/12/1995 | 12/20/1999 | 0 |
| 3634 | M | 1/1/1959 | 3/22/1991 | 11/9/2012 |  | 3/21/1994 | 5/31/2010 | 1 |
| 3637 | M | 1/1/1959 | 10/19/1990 | 2/13/2013 |  | 10/18/1993 | 5/31/2010 | 1 |
| 3643 | M | 1/1/1958 | 3/9/1992 | 3/12/2013 |  | 3/9/1995 | 5/31/2010 | 1 |
| 3645 | M | 1/1/1961 | 9/11/1991 | 12/25/2000 | 9/9/2001 | 9/10/1994 | 9/9/2001 | 0 |
| 3648 | F | 1/1/1961 | 7/3/1991 | 4/15/2013 |  | 7/2/1994 | 5/31/2010 | 1 |
| 3649 | M | 3/1/1997 | 4/29/1997 | 9/5/2009 | 7/18/2010 | 3/1/1997 | 9/5/2009 | 1 |
| 3650 | F | 12/1/2002 | 3/30/2003 | 1/27/2013 |  | 3/30/2003 | 5/31/2010 | 1 |
| 3651 | F | 3/1/2007 | 4/1/2007 | 4/15/2013 |  | 3/1/2007 | 5/31/2010 | 1 |
| 3661 | F | 1/1/1963 | 7/23/1993 | 7/10/1998 | 2/15/1999 | 7/22/1996 | 2/15/1999 | 0 |
| 3663 | F | 1/1/1981 | 7/23/1993 | 7/10/1998 | 4/8/1999 | 7/22/1996 | 4/8/1999 | 1 |
| 3664 | M | 1/1/1990 | 7/23/1993 | 3/30/2000 | 1/6/2001 | 7/22/1996 | 3/30/2000 | 1 |
| 3665 | F | 12/30/1996 | 7/19/1997 | 7/10/1998 | 2/27/1999 | 7/19/1997 | 2/27/1999 | 0 |
| 3677 | F | 1/1/1956 | 6/7/1991 | 5/21/1998 | 10/8/1998 | 6/6/1994 | 10/8/1998 | 0 |
| 3679 | M | 3/1/1987 | 6/7/1991 | 1/6/1998 | 8/16/1998 | 6/6/1994 | 8/16/1998 | 1 |
| 3680 | M | 3/1/1992 | 8/21/1992 | 5/21/1998 | 9/28/1998 | 8/21/1992 | 9/28/1998 | 1 |
| 3681 | F | 1/1/1980 | 3/15/1995 | 5/11/2004 | 3/9/2005 | 3/14/1998 | 3/9/2005 | 0 |
| 3712 | F | 1/1/1952 | 3/14/1992 | 12/26/2009 | 6/20/2010 | 3/14/1995 | 5/31/2010 | 1 |
| 3713 | F | 1/1/1989 | 3/14/1992 | 5/26/2012 | 9/3/2012 | 3/14/1995 | 5/31/2010 | 1 |
| 3714 | M | 4/1/1992 | 4/23/1992 | 12/26/2009 | 11/3/2010 | 4/1/1992 | 5/31/2010 | 1 |
| 3719 | F | 9/2/1999 | 6/3/2000 | 5/24/2004 | 8/24/2004 | 6/3/2000 | 8/24/2004 | 0 |
| 3736 | F | 1/1/1968 | 2/3/1991 | 10/25/2004 | 12/28/2004 | 2/2/1994 | 12/28/2004 | 0 |
| 3738 | F | 1/1/1973 | 7/30/1996 | 2/11/2013 |  | 7/30/1999 | 5/31/2010 | 1 |
| 3741 | F | 1/1/1992 | 7/30/1996 | 10/9/2012 |  | 7/30/1996 | 5/31/2010 | 1 |
| 3742 | F | 1/1/1996 | 7/30/1996 | 10/11/2012 |  | 7/30/1996 | 5/31/2010 | 1 |
| 3743 | F | 4/23/2003 | 5/7/2003 | 10/11/2012 |  | 4/23/2003 | 5/31/2010 | 1 |
| 3745 | F | 6/1/2008 | 11/25/2008 | 2/11/2013 |  | 11/25/2008 | 5/31/2010 | 1 |
| 3746 | M | 1/1/1984 | 2/3/1991 | 11/15/2012 |  | 2/2/1994 | 5/31/2010 | 1 |
| 3747 | F | 1/1/1988 | 2/3/1991 | 1/4/2013 |  | 2/2/1994 | 5/31/2010 | 1 |
| 3748 | F | 10/9/1994 | 11/13/1994 | 1/8/2013 |  | 10/9/1994 | 5/31/2010 | 1 |
| 3750 | M | 7/15/1999 | 8/7/1999 | 10/28/2003 | 1/12/2004 | 7/15/1999 | 1/12/2004 | 0 |
| 3756 | F | 1/1/1955 | 10/3/1991 | 1/10/2013 |  | 10/2/1994 | 5/31/2010 | 1 |
| 3758 | F | 1/1/1985 | 10/3/1991 | 2/23/2013 |  | 10/2/1994 | 5/31/2010 | 1 |
| 3759 | M | 1/1/1987 | 10/3/1991 | 5/26/1999 | 3/23/2000 | 10/2/1994 | 3/23/2000 | 1 |
| 3765 | F | 1/1/1999 | 8/14/2004 | 2/23/2013 |  | 8/14/2004 | 5/31/2010 | 1 |
| 3766 | M | 8/7/1992 | 8/15/1992 | 4/15/2013 |  | 8/7/1992 | 5/31/2010 | 1 |
| 3767 | F | 12/30/1999 | 5/6/2000 | 1/10/2013 |  | 5/6/2000 | 5/31/2010 | 1 |
| 3769 | F | 1/1/1975 | 11/9/1992 | 3/15/2013 |  | 11/9/1995 | 5/31/2010 | 1 |
| 3770 | F | 8/1/2000 | 11/21/2000 | 3/15/2013 |  | 11/21/2000 | 5/31/2010 | 1 |
| 3784 | M | 1/1/1962 | 7/26/1992 | 7/19/1999 | 5/10/2000 | 7/26/1995 | 5/10/2000 | 0 |
| 3785 | F | 1/1/1970 | 11/10/1990 | 12/6/1998 | 6/20/1999 | 11/9/1993 | 6/20/1999 | 0 |
| 3786 | F | 11/1/1986 | 11/10/1990 | 8/4/1995 | 12/10/1996 | 11/9/1993 | 12/10/1996 | 1 |
| 3787 | F | 1/1/1959 | 1/8/1995 | 1/28/2012 |  | 1/7/1998 | 5/31/2010 | 1 |
| 3788 | F | 9/1/1993 | 1/8/1995 | 12/19/2011 |  | 1/8/1995 | 5/31/2010 | 1 |
| 3789 | M | 4/1/1999 | 8/10/1999 | 7/24/2009 | 4/28/2010 | 8/10/1999 | 4/28/2010 | 1 |
| 3790 | M | 8/19/2007 | 1/25/2008 | 1/28/2012 |  | 1/25/2008 | 5/31/2010 | 1 |
| 3796 | F | 1/1/1972 | 4/13/1993 | 11/4/2012 |  | 4/12/1996 | 5/31/2010 | 1 |
| 3798 | F | 6/1/1992 | 4/13/1993 | 1/27/2008 | 7/29/2008 | 4/13/1993 | 7/29/2008 | 1 |
| 3799 | M | 8/21/2000 | 2/4/2001 | 12/11/2011 |  | 2/4/2001 | 5/31/2010 | 1 |
| 3800 | M | 8/24/2006 | 11/23/2006 | 11/4/2012 |  | 11/23/2006 | 5/31/2010 | 1 |
| 3801 | M | 1/1/1957 | 9/6/1992 | 8/16/2003 | 11/16/2003 | 9/6/1995 | 11/16/2003 | 0 |
| 3809 | M | 1/1/1971 | 2/9/1994 | 3/21/2013 |  | 2/8/1997 | 5/31/2010 | 1 |
| 3817 | F | 1/1/1955 | 3/25/1991 | 3/21/2013 |  | 3/24/1994 | 5/31/2010 | 1 |
| 3818 | M | 1/1/1975 | 3/25/1991 | 4/28/2003 | 9/7/2003 | 3/24/1994 | 9/7/2003 | 0 |
| 3819 | F | 3/1/1989 | 3/25/1991 | 3/21/2013 |  | 3/24/1994 | 5/31/2010 | 1 |
| 3820 | F | 6/1/1994 | 7/27/1994 | 12/3/2012 |  | 6/1/1994 | 5/31/2010 | 1 |
| 3821 | M | 4/1/2001 | 3/8/2002 | 12/3/2012 |  | 3/8/2002 | 5/31/2010 | 1 |
| 3822 | F | 9/10/2006 | 10/22/2006 | 1/27/2013 |  | 9/10/2006 | 5/31/2010 | 1 |
| 3830 | M | 1/1/1951 | 2/26/1991 | 3/5/1997 | 1/19/1998 | 2/25/1994 | 1/19/1998 | 0 |
| 3831 | F | 1/1/1958 | 1/22/1991 | 5/21/2010 | 8/9/2010 | 1/21/1994 | 5/31/2010 | 1 |
| 3833 | F | 5/1/1992 | 10/27/1992 | 5/21/2010 | 8/17/2010 | 10/27/1992 | 5/31/2010 | 1 |
| 3834 | F | 8/4/1996 | 9/20/1996 | 5/21/2010 | 8/9/2010 | 8/4/1996 | 5/31/2010 | 1 |
| 3835 | M | 5/6/2000 | 6/21/2000 | 5/15/2010 | 9/26/2010 | 5/6/2000 | 5/31/2010 | 1 |
| 3836 | M | 8/1/2003 | 8/18/2003 | 5/21/2010 | 8/25/2010 | 8/1/2003 | 5/31/2010 | 1 |
| 3837 | F | 10/1/2006 | 11/29/2006 | 5/21/2010 | 9/8/2010 | 10/1/2006 | 5/31/2010 | 0 |
| 3843 | F | 1/1/1970 | 7/30/1996 | 2/26/2013 |  | 7/30/1999 | 5/31/2010 | 1 |
| 3844 | F | 5/15/1994 | 7/30/1996 | 2/26/2013 |  | 7/30/1996 | 5/31/2010 | 1 |
| 3860 | M | 1/1/1971 | 9/27/1991 | 1/6/1997 | 8/18/1997 | 9/26/1994 | 8/18/1997 | 0 |
| 3862 | F | 1/1/1946 | 1/13/1991 | 2/15/1998 | 10/15/1998 | 1/12/1994 | 10/15/1998 | 0 |
| 3863 | F | 1/1/1980 | 1/13/1991 | 2/25/1996 | 10/8/1996 | 1/12/1994 | 10/8/1996 | 1 |
| 3864 | M | 1/1/1990 | 1/13/1991 | 1/24/1997 | 9/7/1997 | 1/12/1994 | 9/7/1997 | 1 |
| 3867 | M | 1/1/1974 | 1/8/1991 | 1/12/1997 | 4/10/1997 | 1/7/1994 | 4/10/1997 | 0 |
| 3870 | F | 1/1/1964 | 2/22/1991 | 3/21/2013 |  | 2/21/1994 | 5/31/2010 | 1 |
| 3877 | F | 1/1/1979 | 9/23/1991 | 12/18/2011 | 3/23/2012 | 2/21/1994 | 5/31/2010 | 1 |
| 3878 | M | 1/1/1986 | 2/22/1991 | 5/19/2010 | 8/9/2010 | 2/21/1994 | 5/31/2010 | 1 |
| 3883 | F | 1/29/1997 | 2/16/1997 | 3/21/2013 |  | 1/29/1997 | 5/31/2010 | 1 |
| 3884 | F | 5/1/2001 | 5/4/2001 | 3/21/2013 |  | 5/1/2001 | 5/31/2010 | 1 |
| 3885 | F | 4/1/2006 | 4/14/2006 | 3/21/2013 |  | 4/1/2006 | 5/31/2010 | 1 |
| 3886 | F | 1/1/1969 | 7/31/1992 | 11/15/2012 |  | 7/31/1995 | 5/31/2010 | 1 |
| 3887 | F | 1/1/1992 | 7/31/1992 | 10/24/2012 |  | 7/31/1992 | 5/31/2010 | 1 |
| 3888 | F | 10/1/2000 | 10/28/2000 | 11/15/2012 |  | 10/1/2000 | 5/31/2010 | 1 |
| 3890 | F | 1/12/2000 | 4/14/2007 | 11/15/2012 |  | 4/14/2007 | 5/31/2010 | 1 |
| 3891 | F | 6/1/2006 | 5/4/2007 | 11/15/2012 |  | 5/4/2007 | 5/31/2010 | 1 |
| 3892 | F | 1/1/1985 | 1/22/1991 | 9/5/1998 | 9/6/1999 | 1/21/1994 | 9/6/1999 | 0 |
| 3898 | F | 1/1/1965 | 1/13/1991 | 2/13/2013 |  | 1/12/1994 | 5/31/2010 | 1 |
| 3899 | F | 1/1/1987 | 12/12/1991 | 3/15/2013 |  | 1/12/1994 | 5/31/2010 | 1 |
| 3900 | F | 4/1/2007 | 12/6/2007 | 3/15/2013 |  | 12/6/2007 | 5/31/2010 | 1 |
| 3901 | F | 9/1/1990 | 1/13/1991 | 2/13/2013 |  | 1/13/1991 | 5/31/2010 | 1 |
| 3902 | F | 7/9/1993 | 11/29/1997 | 2/20/2012 |  | 11/29/1997 | 5/31/2010 | 1 |
| 3903 | F | 1/1/1942 | 4/21/1992 | 1/17/2000 | 4/4/2000 | 4/21/1995 | 4/4/2000 | 0 |
| 3904 | F | 1/1/1980 | 12/31/1992 | 3/22/2013 |  | 4/21/1995 | 5/31/2010 | 1 |
| 3905 | F | 9/7/1996 | 4/25/1997 | 3/22/2013 |  | 4/25/1997 | 5/31/2010 | 1 |
| 3906 | F | 7/18/2002 | 9/19/2002 | 3/22/2013 |  | 9/19/2002 | 5/31/2010 | 1 |
| 3907 | M | 5/22/2008 | 6/1/2008 | 2/12/2013 |  | 5/22/2008 | 5/31/2010 | 1 |
| 3928 | F | 6/20/1999 | 1/27/2001 | 12/30/2012 |  | 1/27/2001 | 5/31/2010 | 1 |
| 3929 | M | 7/1/2003 | 11/15/2003 | 2/20/2012 |  | 11/15/2003 | 5/31/2010 | 1 |
| 3933 | M | 1/1/1955 | 10/20/1990 | 5/22/1996 | 9/25/1996 | 10/19/1993 | 9/25/1996 | 0 |
| 3936 | M | 1/1/1997 | 10/24/2002 | 7/14/2009 | 2/7/2010 | 10/24/2002 | 2/7/2010 | 0 |
| 3943 | M | 1/1/1957 | 9/4/1992 | 9/24/1995 | 5/1/1996 | 9/4/1995 | 5/1/1996 | 0 |
| 3959 | M | 1/1/1984 | 6/16/1994 | 9/6/2002 | 3/9/2003 | 6/15/1997 | 3/9/2003 | 0 |
| 3961 | F | 1/1/1960 | 1/14/1991 | 1/11/2003 | 6/2/2003 | 1/13/1994 | 6/2/2003 | 0 |
| 3962 | F | 1/1/1975 | 1/24/1993 | 5/23/2001 | 9/26/2001 | 1/13/1994 | 9/26/2001 | 0 |
| 3963 | F | 9/1/1990 | 1/14/1991 | 2/21/1996 | 5/30/1997 | 1/14/1991 | 5/30/1997 | 1 |
| 3966 | M | 7/19/1994 | 5/23/1995 | 7/2/2002 | 1/25/2004 | 5/23/1995 | 1/25/2004 | 1 |
| 3967 | M | 10/5/1998 | 1/6/2000 | 1/11/2003 | 4/7/2003 | 1/6/2000 | 4/7/2003 | 0 |
| 3969 | F | 1/1/1954 | 1/28/1992 | 3/4/2004 | 2/1/2005 | 1/27/1995 | 2/1/2005 | 0 |
| 3970 | F | 1/1/1980 | 1/28/1992 | 2/7/1999 | 7/1/2000 | 1/27/1995 | 7/1/2000 | 1 |
| 3971 | M | 1/1/1985 | 1/28/1992 | 4/8/2002 | 10/30/2003 | 1/27/1995 | 10/30/2003 | 0 |
| 3972 | M | 7/1/1988 | 1/28/1992 | 1/24/1999 | 4/30/2000 | 1/27/1995 | 4/30/2000 | 1 |
| 3973 | M | 9/5/1992 | 2/9/1993 | 3/20/2002 | 8/10/2003 | 2/9/1993 | 8/10/2003 | 1 |
| 3974 | F | 1/18/1997 | 2/11/1998 | 2/11/2004 | 6/5/2005 | 2/11/1998 | 6/5/2005 | 1 |
| 3975 | M | 10/2/2000 | 2/20/2002 | 3/4/2004 | 9/18/2004 | 2/20/2002 | 9/18/2004 | 0 |
| 3982 | F | 1/1/1960 | 12/13/1991 | 7/22/2001 | 10/20/2001 | 12/12/1994 | 10/20/2001 | 0 |
| 3983 | M | 1/1/1991 | 12/13/1991 | 11/3/1999 | 2/25/2000 | 12/13/1991 | 2/25/2000 | 0 |
| 3984 | F | 12/12/1997 | 3/25/1998 | 7/22/2001 | 10/16/2001 | 3/25/1998 | 10/16/2001 | 0 |
| 3986 | F | 1/1/1958 | 3/29/1992 | 6/6/2002 | 10/14/2002 | 3/29/1995 | 10/14/2002 | 0 |
| 3987 | M | 1/1/1980 | 3/29/1992 | 5/19/1999 | 2/29/2000 | 3/29/1995 | 2/29/2000 | 1 |
| 3988 | F | 1/1/1990 | 3/29/1992 | 11/22/2006 | 5/15/2007 | 3/29/1995 | 5/15/2007 | 0 |
| 3990 | F | 3/1/1996 | 3/25/1996 | 4/16/2003 | 7/27/2003 | 3/1/1996 | 4/16/2003 | 1 |
| 3992 | M | 1/1/1968 | 8/2/1993 | 2/28/2003 | 7/22/2003 | 8/1/1996 | 7/22/2003 | 0 |
| 4009 | F | 1/1/1955 | 9/9/1991 | 11/13/2007 | 5/15/2008 | 9/8/1994 | 5/15/2008 | 0 |
| 4010 | F | 9/1/1988 | 9/9/1991 | 10/28/2001 | 5/24/2002 | 9/8/1994 | 5/24/2002 | 1 |
| 4011 | F | 1/1/1987 | 7/29/1994 | 3/14/2013 |  | 9/8/1994 | 5/31/2010 | 1 |
| 4013 | M | 8/26/1999 | 11/29/1999 | 12/23/2010 |  | 11/29/1999 | 5/31/2010 | 1 |
| 4023 | F | 1/1/1937 | 3/29/1992 | 10/6/1997 | 5/14/1998 | 3/29/1995 | 5/14/1998 | 0 |
| 4029 | M | 1/1/1960 | 5/1/1990 | 10/6/1996 | 6/23/1997 | 4/30/1993 | 6/23/1997 | 0 |
| 4030 | F | 1/1/1959 | 1/12/1992 | 2/28/2013 |  | 1/11/1995 | 5/31/2010 | 1 |
| 4031 | F | 1/1/1961 | 3/5/1991 | 12/3/2012 |  | 3/4/1994 | 5/31/2010 | 1 |
| 4032 | F | 1/1/1989 | 3/5/1991 | 5/12/2001 | 3/28/2002 | 3/4/1994 | 3/28/2002 | 0 |
| 4033 | F | 9/1/1991 | 9/22/1991 | 4/9/2012 |  | 9/1/1991 | 5/31/2010 | 1 |
| 4036 | F | 4/1/1998 | 5/3/1998 | 12/3/2012 |  | 4/1/1998 | 5/31/2010 | 1 |
| 4037 | M | 5/1/2003 | 1/28/2004 | 12/3/2012 |  | 1/28/2004 | 5/31/2010 | 1 |
| 4039 | M | 1/1/2001 | 9/29/2005 | 2/25/2010 | 7/9/2010 | 9/29/2005 | 5/31/2010 | 1 |
| 4040 | M | 1/1/1996 | 11/4/2001 | 1/18/2013 |  | 11/4/2001 | 5/31/2010 | 1 |
| 4050 | F | 1/1/1966 | 6/4/1991 | 4/26/1999 | 8/8/1999 | 6/3/1994 | 8/8/1999 | 0 |
| 4054 | F | 1/1/1985 | 6/4/1991 | 5/13/2010 | 8/13/2010 | 6/3/1994 | 5/31/2010 | 1 |
| 4056 | F | 1/10/1993 | 3/24/1993 | 3/27/2010 | 7/7/2010 | 3/24/1993 | 5/31/2010 | 1 |
| 4070 | F | 1/1/1965 | 1/25/1993 | 12/3/2012 |  | 1/25/1996 | 5/31/2010 | 1 |
| 4072 | F | 1/1/1988 | 1/25/1993 | 5/19/2010 |  | 1/25/1996 | 5/31/2010 | 1 |
| 4073 | M | 1/1/1992 | 1/25/1993 | 11/23/2011 |  | 1/25/1993 | 5/31/2010 | 1 |
| 4074 | F | 7/14/1999 | 7/31/1999 | 12/3/2012 |  | 7/14/1999 | 5/31/2010 | 1 |
| 4075 | M | 4/1/2006 | 7/4/2006 | 12/3/2012 |  | 7/4/2006 | 5/31/2010 | 1 |
| 4083 | F | 1/1/1980 | 12/20/2000 | 4/15/2013 |  | 12/20/2003 | 5/31/2010 | 1 |
| 4084 | M | 5/20/2009 | 6/28/2009 | 4/15/2013 |  | 5/20/2009 | 5/31/2010 | 1 |
| 4087 | F | 1/1/1963 | 3/12/1992 | 4/15/2013 |  | 3/12/1995 | 5/31/2010 | 1 |
| 4088 | F | 1/1/1986 | 3/12/1992 | 1/4/2013 |  | 3/12/1995 | 5/31/2010 | 1 |
| 4089 | M | 2/1/1998 | 4/7/1998 | 10/16/2006 | 4/30/2007 | 4/7/1998 | 4/30/2007 | 1 |
| 4094 | M | 4/1/2007 | 4/13/2007 | 4/15/2013 |  | 4/1/2007 | 5/31/2010 | 1 |
| 4099 | F | 1/1/1959 | 11/9/1991 | 4/15/2013 |  | 11/8/1994 | 5/31/2010 | 1 |
| 4102 | F | 12/1/1987 | 11/9/1991 | 3/1/2007 | 10/25/2007 | 11/8/1994 | 10/25/2007 | 1 |
| 4103 | M | 11/1/1992 | 11/19/1992 | 10/31/2002 | 4/5/2003 | 11/1/1992 | 4/5/2003 | 1 |
| 4104 | F | 5/8/1996 | 8/10/1997 | 1/1/2013 |  | 8/10/1997 | 5/31/2010 | 1 |
| 4105 | M | 5/1/2002 | 6/13/2002 | 4/15/2013 |  | 5/1/2002 | 5/31/2010 | 1 |
| 4106 | M | 1/1/1940 | 11/9/1990 | 6/29/2002 | 3/8/2003 | 11/8/1993 | 3/8/2003 | 0 |
| 4109 | F | 1/1/1983 | 1/7/2002 | 3/22/2013 |  | 1/6/2005 | 5/31/2010 | 1 |
| 4112 | F | 1/1/1965 | 3/14/1992 | 5/4/2012 | 8/22/2012 | 3/14/1995 | 5/31/2010 | 1 |
| 4115 | M | 1/1/1989 | 3/14/1992 | 3/19/2013 |  | 3/14/1995 | 5/31/2010 | 1 |
| 4116 | M | 4/3/1997 | 4/4/1997 | 2/29/2012 |  | 4/3/1997 | 5/31/2010 | 1 |
| 4117 | M | 4/1/2003 | 4/7/2004 | 5/4/2012 |  | 4/7/2004 | 5/31/2010 | 1 |
| 4125 | F | 1/1/1943 | 4/15/1992 | 11/11/1995 | 11/13/1996 | 4/15/1995 | 11/13/1996 | 0 |
| 4126 | M | 1/1/1990 | 4/15/1992 | 11/11/1995 | 4/14/1997 | 4/15/1995 | 4/14/1997 | 1 |
| 4127 | M | 8/10/1993 | 2/17/1994 | 6/21/1994 | 10/17/1994 | 2/17/1994 | 10/17/1994 | 0 |
| 4129 | M | 1/1/1956 | 9/14/1991 | 10/5/1996 | 6/20/1997 | 9/13/1994 | 6/20/1997 | 0 |
| 4134 | M | 1/1/1974 | 4/28/1995 | 11/30/2012 |  | 4/27/1998 | 5/31/2010 | 1 |
| 4140 | F | 1/1/1983 | 12/8/1997 | 5/16/2003 | 8/6/2003 | 12/7/2000 | 8/6/2003 | 0 |
| 4141 | M | 1/8/2000 | 1/12/2000 | 5/16/2003 | 7/15/2003 | 1/8/2000 | 7/15/2003 | 0 |
| 4148 | F | 1/1/1958 | 10/24/1992 | 5/21/2009 | 9/26/2009 | 10/24/1995 | 9/26/2009 | 0 |
| 4149 | F | 1/1/1983 | 10/24/1992 | 10/18/2003 | 2/1/2004 | 10/24/1995 | 2/1/2004 | 0 |
| 4150 | F | 3/1/1998 | 4/19/1998 | 6/27/2006 | 3/24/2007 | 3/1/1998 | 3/24/2007 | 0 |
| 4151 | F | 1/1/1987 | 10/24/1992 | 5/4/2003 | 8/24/2003 | 10/24/1995 | 8/24/2003 | 0 |
| 4152 | F | 5/6/2002 | 5/10/2002 | 11/8/2003 | 2/4/2004 | 5/6/2002 | 2/4/2004 | 0 |
| 4153 | F | 1/3/1994 | 4/16/1994 | 5/2/2006 | 8/28/2006 | 4/16/1994 | 8/28/2006 | 0 |
| 4154 | F | 6/1/2003 | 10/16/2003 | 11/20/2008 | 5/25/2009 | 10/16/2003 | 5/25/2009 | 1 |
| 4156 | F | 1/1/1966 | 1/2/1991 | 12/27/2002 | 5/28/2003 | 1/1/1994 | 5/28/2003 | 0 |
| 4157 | F | 1/1/1985 | 1/2/1991 | 9/11/2009 | 1/21/2010 | 1/1/1994 | 1/21/2010 | 0 |
| 4158 | M | 10/10/1999 | 3/4/2000 | 9/11/2009 | 1/23/2010 | 3/4/2000 | 1/23/2010 | 0 |
| 4159 | M | 12/1/2004 | 1/3/2005 | 9/11/2009 | 1/25/2010 | 12/1/2004 | 1/25/2010 | 0 |
| 4160 | F | 3/1/1992 | 8/31/1992 | 3/28/2010 | 9/10/2010 | 8/31/1992 | 5/31/2010 | 1 |
| 4162 | M | 5/5/1999 | 5/17/1999 | 6/5/2000 | 9/15/2001 | 5/5/1999 | 9/15/2001 | 0 |
| 4169 | F | 1/1/1971 | 4/17/1992 | 3/22/2013 |  | 4/17/1995 | 5/31/2010 | 1 |
| 4170 | M | 1/1/1991 | 4/17/1992 | 9/17/2005 | 1/17/2006 | 4/17/1992 | 1/17/2006 | 0 |
| 4171 | M | 6/1/2002 | 10/21/2002 | 3/22/2013 |  | 10/21/2002 | 5/31/2010 | 1 |
| 4177 | M | 1/1/1970 | 1/14/1995 | 7/14/1998 | 9/5/1999 | 1/13/1998 | 9/5/1999 | 0 |
| 4181 | M | 1/1/1958 | 1/12/1993 | 3/20/1997 | 9/2/1997 | 1/12/1996 | 9/2/1997 | 0 |
| 4184 | F | 1/1/1986 | 5/1/1990 | 4/15/2013 |  | 4/30/1993 | 5/31/2010 | 1 |
| 4185 | F | 1/1/1985 | 5/1/1990 | 3/22/2013 |  | 4/30/1993 | 5/31/2010 | 1 |
| 4186 | F | 8/1/2002 | 9/13/2002 | 3/22/2013 |  | 8/1/2002 | 5/31/2010 | 1 |
| 4187 | F | 3/1/2008 | 3/22/2008 | 3/22/2013 |  | 3/1/2008 | 5/31/2010 | 1 |
| 4188 | F | 5/1/1988 | 10/24/1990 | 3/22/2013 |  | 4/30/1993 | 5/31/2010 | 1 |
| 4189 | M | 12/21/2006 | 3/20/2007 | 12/9/2011 |  | 3/20/2007 | 5/31/2010 | 1 |
| 4190 | F | 3/10/2009 | 3/10/2009 | 3/22/2013 |  | 3/10/2009 | 5/31/2010 | 1 |
| 4191 | M | 6/12/1994 | 8/10/1994 | 2/29/2012 | 7/16/2012 | 6/12/1994 | 5/31/2010 | 1 |
| 4192 | M | 6/26/1999 | 8/26/1999 | 2/29/2012 | 6/28/2012 | 8/26/1999 | 5/31/2010 | 1 |
| 4193 | M | 6/27/2003 | 9/2/2003 | 11/18/2012 |  | 9/2/2003 | 5/31/2010 | 1 |
| 4205 | F | 1/1/1961 | 4/21/1993 | 3/17/2013 |  | 4/20/1996 | 5/31/2010 | 1 |
| 4206 | F | 4/1/1981 | 4/21/1993 | 3/10/2009 |  | 4/20/1996 | 5/31/2010 | 1 |
| 4207 | M | 2/13/1995 | 2/7/1997 | 3/10/2009 | 9/11/2010 | 2/7/1997 | 5/31/2010 | 1 |
| 4208 | M | 8/29/2001 | 3/7/2002 | 2/26/2013 |  | 3/7/2002 | 5/31/2010 | 1 |
| 4212 | F | 1/1/1957 | 3/5/1992 | 2/11/2005 | 8/16/2005 | 3/5/1995 | 8/16/2005 | 0 |
| 4213 | F | 12/13/1995 | 3/27/1996 | 2/11/2005 | 10/3/2005 | 3/27/1996 | 10/3/2005 | 1 |
| 4214 | F | 6/1/2003 | 3/1/2004 | 2/11/2005 | 12/16/2005 | 3/1/2004 | 12/16/2005 | 0 |
| 4243 | F | 1/1/1941 | 7/31/1991 | 8/2/2000 | 6/28/2001 | 7/30/1994 | 6/28/2001 | 0 |
| 4244 | F | 1/1/1988 | 7/31/1991 | 8/2/2000 | 6/30/2001 | 7/30/1994 | 6/30/2001 | 1 |
| 4245 | F | 1/1/1952 | 6/5/1991 | 5/17/2009 | 8/31/2009 | 6/4/1994 | 8/31/2009 | 0 |
| 4246 | F | 1/1/1967 | 5/30/1993 | 11/25/2012 |  | 6/4/1994 | 5/31/2010 | 1 |
| 4247 | F | 2/1/1996 | 3/6/1996 | 11/25/2012 |  | 2/1/1996 | 5/31/2010 | 1 |
| 4248 | F | 4/18/2001 | 5/14/2001 | 11/25/2012 |  | 4/18/2001 | 5/31/2010 | 1 |
| 4249 | F | 1/1/1985 | 6/5/1991 | 11/25/2012 |  | 6/4/1994 | 5/31/2010 | 1 |
| 4250 | M | 1/17/2000 | 4/10/2000 | 11/25/2012 |  | 4/10/2000 | 5/31/2010 | 1 |
| 4251 | F | 4/1/2005 | 9/20/2005 | 11/16/2012 |  | 9/20/2005 | 5/31/2010 | 1 |
| 4252 | F | 11/16/2003 | 5/5/2004 | 11/25/2012 |  | 5/5/2004 | 5/31/2010 | 1 |
| 4254 | F | 3/22/1992 | 8/5/1992 | 4/15/2013 |  | 8/5/1992 | 5/31/2010 | 1 |
| 4259 | M | 1/1/1976 | 4/4/1994 | 10/16/2006 | 8/18/2007 | 4/3/1997 | 8/18/2007 | 0 |
| 4265 | F | 1/1/1962 | 9/23/1991 | 12/29/2012 |  | 9/22/1994 | 5/31/2010 | 1 |
| 4266 | F | 1/1/1990 | 9/23/1991 | 6/25/2001 | 1/23/2002 | 9/22/1994 | 6/25/2001 | 1 |
| 4267 | M | 10/1/1994 | 11/16/1994 | 6/25/2001 | 11/24/2001 | 10/1/1994 | 11/24/2001 | 1 |
| 4268 | M | 7/29/1998 | 12/27/1998 | 10/28/2001 | 2/7/2002 | 12/27/1998 | 2/7/2002 | 0 |
| 4271 | F | 1/1/1953 | 1/13/1994 | 8/21/2009 | 3/19/2010 | 1/12/1997 | 3/19/2010 | 0 |
| 4272 | F | 1/1/1990 | 1/13/1994 | 2/24/2013 |  | 1/12/1997 | 5/31/2010 | 1 |
| 4273 | M | 4/1/1994 | 5/19/1996 | 1/29/2005 | 11/9/2005 | 5/19/1996 | 11/9/2005 | 0 |
| 4278 | M | 3/28/2000 | 6/19/2000 | 12/9/2008 | 5/1/2010 | 6/19/2000 | 5/1/2010 | 1 |
| 4279 | M | 12/1/2005 | 12/27/2005 | 8/21/2009 | 3/7/2010 | 12/1/2005 | 3/7/2010 | 0 |
| 4280 | F | 1/1/1937 | 3/18/1992 | 5/31/1995 | 3/14/1996 | 3/18/1995 | 3/14/1996 | 0 |
| 4281 | F | 11/1/1980 | 12/16/1993 | 5/26/1995 | 5/8/1996 | 3/18/1995 | 5/8/1996 | 1 |
| 4282 | M | 1/1/1990 | 3/18/1992 | 5/31/1995 | 3/14/1996 | 3/18/1995 | 3/14/1996 | 1 |
| 4285 | M | 1/1/1973 | 12/12/1994 | 1/19/2013 |  | 12/11/1997 | 5/31/2010 | 1 |
| 4290 | M | 1/1/1980 | 9/19/1992 | 8/29/1997 | 5/12/1998 | 9/19/1995 | 5/12/1998 | 0 |
| 4298 | M | 1/1/1957 | 8/31/1992 | 8/8/2003 | 5/10/2004 | 8/31/1995 | 5/10/2004 | 0 |
| 4299 | M | 1/1/1935 | 10/15/1990 | 9/5/1996 | 4/7/1997 | 10/14/1993 | 4/7/1997 | 0 |
| 4303 | F | 1/1/1964 | 11/24/1992 | 5/23/2001 | 3/13/2002 | 11/24/1995 | 3/13/2002 | 0 |
| 4304 | F | 4/1/1991 | 11/24/1992 | 2/5/1999 | 8/24/2000 | 11/24/1992 | 8/24/2000 | 1 |
| 4305 | F | 2/1/1995 | 8/10/1995 | 4/25/1996 | 6/5/1996 | 8/10/1995 | 6/5/1996 | 0 |
| 4306 | M | 5/14/1997 | 3/9/1998 | 5/23/2001 | 1/8/2002 | 3/9/1998 | 1/8/2002 | 0 |
| 4312 | M | 1/1/1964 | 1/18/1991 | 12/14/2007 | 1/19/2009 | 1/17/1994 | 1/19/2009 | 0 |
| 4318 | F | 1/1/1930 | 9/7/1991 | 3/3/1996 | 8/2/1996 | 9/6/1994 | 8/2/1996 | 0 |
| 4327 | M | 1/1/1963 | 2/27/1992 | 1/28/2013 |  | 2/26/1995 | 5/31/2010 | 1 |
| 4331 | F | 1/1/1980 | 1/15/2004 | 8/22/2009 | 7/22/2010 | 1/14/2007 | 5/31/2010 | 1 |
| 4332 | F | 1/1/1992 | 5/4/2004 | 1/22/2008 |  | 5/4/2004 | 5/31/2010 | 1 |
| 4333 | M | 1/1/1996 | 8/28/2004 | 8/22/2009 | 4/14/2011 | 8/28/2004 | 5/31/2010 | 1 |
| 4339 | F | 1/1/1981 | 11/7/2005 | 1/28/2013 |  | 11/6/2008 | 5/31/2010 | 1 |
| 4340 | M | 5/18/2007 | 11/5/2007 | 1/28/2013 |  | 11/5/2007 | 5/31/2010 | 1 |
| 4345 | F | 1/1/1956 | 12/28/1993 | 11/12/2012 |  | 12/27/1996 | 5/31/2010 | 1 |
| 4348 | F | 3/21/2002 | 3/21/2002 | 2/26/2013 |  | 3/21/2002 | 5/31/2010 | 1 |
| 4350 | M | 1/1/1989 | 12/28/1993 | 10/21/2012 |  | 12/27/1996 | 5/31/2010 | 1 |
| 4351 | F | 4/15/1999 | 7/12/1999 | 11/12/2012 |  | 7/12/1999 | 5/31/2010 | 1 |
| 4356 | F | 1/1/1965 | 10/12/1992 | 10/6/1997 | 2/5/1998 | 10/12/1995 | 2/5/1998 | 0 |
| 4358 | F | 1/1/1978 | 10/12/1992 | 5/24/1997 | 3/22/1998 | 10/12/1995 | 3/22/1998 | 1 |
| 4359 | M | 1/1/1986 | 10/12/1992 | 5/24/1997 | 10/15/1997 | 10/12/1995 | 10/15/1997 | 1 |
| 4360 | F | 10/1/1990 | 10/12/1992 | 10/6/1997 | 2/23/1998 | 10/12/1992 | 2/23/1998 | 1 |
| 4362 | F | 1/1/1963 | 9/6/1991 | 12/29/1996 | 7/25/1997 | 9/5/1994 | 7/25/1997 | 0 |
| 4363 | M | 1/1/1979 | 9/6/1991 | 12/29/1996 | 7/15/1997 | 9/5/1994 | 12/29/1996 | 1 |
| 4364 | F | 1/1/1990 | 9/6/1991 | 3/27/1996 | 10/1/1996 | 9/5/1994 | 10/1/1996 | 1 |
| 4365 | M | 4/5/1994 | 4/18/1994 | 12/29/1996 | 8/2/1997 | 4/5/1994 | 8/2/1997 | 0 |
| 4406 | F | 1/1/1990 | 12/13/2004 | 4/15/2013 |  | 12/13/2007 | 5/31/2010 | 1 |
| 4428 | F | 1/1/1971 | 4/10/1999 | 12/3/2012 |  | 4/9/2002 | 5/31/2010 | 1 |
| 4756 | F | 3/15/2004 | 4/8/2004 | 12/3/2012 |  | 3/15/2004 | 5/31/2010 | 1 |
| 4758 | M | 8/9/2007 | 9/20/2007 | 9/20/2007 | 9/20/2007 | 8/9/2007 | 9/20/2007 | 0 |
| 4759 | M | 6/1/2008 | 12/2/2008 | 9/17/2012 |  | 12/2/2008 | 5/31/2010 | 1 |
| 4762 | F | 5/1/1993 | 9/22/1993 | 10/9/1996 | 1/31/1997 | 9/22/1993 | 1/31/1997 | 0 |
| 4775 | F | 4/1/1999 | 4/5/1999 | 10/11/2010 |  | 4/1/1999 | 5/31/2010 | 1 |
| 4776 | M | 11/1/2003 | 7/7/2005 | 2/28/2012 | 8/30/2012 | 7/7/2005 | 5/31/2010 | 1 |
| 4777 | M | 2/1/2009 | 4/5/2009 | 3/22/2013 |  | 4/5/2009 | 5/31/2010 | 1 |
| 4778 | F | 10/1/2002 | 3/15/2003 | 3/12/2013 |  | 3/15/2003 | 5/31/2010 | 1 |
| 4779 | M | 8/1/2007 | 8/4/2007 | 3/12/2013 |  | 8/1/2007 | 5/31/2010 | 1 |
| 4780 | M | 9/11/1999 | 10/30/1999 | 4/17/2000 | 4/26/2000 | 9/11/1999 | 4/26/2000 | 0 |
| 4781 | M | 5/1/2003 | 5/15/2003 | 3/6/2013 |  | 5/1/2003 | 5/31/2010 | 1 |
| 4793 | F | 4/1/2004 | 12/13/2004 | 4/15/2013 |  | 12/13/2004 | 5/31/2010 | 1 |
| 4795 | M | 7/17/2003 | 9/4/2003 | 4/9/2008 | 2/3/2009 | 7/17/2003 | 2/3/2009 | 0 |
| 4798 | F | 12/23/2000 | 1/24/2002 | 4/10/2002 | 9/9/2002 | 1/24/2002 | 9/9/2002 | 0 |
| 4804 | F | 1/1/2002 | 10/24/2002 | 11/26/2012 |  | 10/24/2002 | 5/31/2010 | 1 |
| 4805 | F | 9/8/2004 | 9/9/2004 | 11/26/2012 |  | 9/8/2004 | 5/31/2010 | 1 |
| 4811 | F | 7/4/2003 | 9/27/2003 | 3/9/2013 |  | 9/27/2003 | 5/31/2010 | 1 |
| 4812 | M | 9/1/2005 | 9/22/2005 | 3/9/2013 |  | 9/1/2005 | 5/31/2010 | 1 |
| 4814 | M | 1/18/2008 | 11/5/2008 | 10/13/2012 |  | 11/5/2008 | 5/31/2010 | 1 |
| 4815 | M | 9/1/2007 | 10/19/2007 | 1/8/2013 |  | 9/1/2007 | 5/31/2010 | 1 |
| 4816 | F | 10/1/1994 | 4/21/1995 | 11/14/2012 |  | 4/21/1995 | 5/31/2010 | 1 |
| 4817 | M | 10/4/2003 | 9/24/2004 | 3/23/2013 |  | 9/24/2004 | 5/31/2010 | 1 |
| 4818 | F | 1/26/1995 | 3/15/1995 | 8/19/1995 | 8/23/1995 | 1/26/1995 | 8/23/1995 | 0 |
| 4825 | M | 10/7/1998 | 11/25/1998 | 5/24/2001 | 10/17/2001 | 10/7/1998 | 10/17/2001 | 0 |
| 4826 | M | 6/1/2000 | 12/3/2000 | 3/2/2012 |  | 12/3/2000 | 5/31/2010 | 1 |
| 4827 | M | 10/3/2007 | 10/17/2007 | 11/20/2012 |  | 10/3/2007 | 5/31/2010 | 1 |
| 4828 | F | 4/8/1999 | 7/10/1999 | 11/4/2012 |  | 7/10/1999 | 5/31/2010 | 1 |
| 4829 | F | 1/1/2004 | 2/7/2004 | 11/4/2012 |  | 1/1/2004 | 5/31/2010 | 1 |
| 4830 | M | 10/1/2008 | 12/10/2008 | 11/4/2012 |  | 12/10/2008 | 5/31/2010 | 1 |
| 4831 | M | 11/7/2001 | 4/9/2002 | 12/8/2011 |  | 4/9/2002 | 5/31/2010 | 1 |
| 4832 | F | 9/16/2007 | 3/30/2008 | 2/22/2012 |  | 3/30/2008 | 5/31/2010 | 1 |
| 4834 | F | 4/1/2006 | 5/30/2006 | 11/12/2012 |  | 4/1/2006 | 5/31/2010 | 1 |
| 4836 | M | 6/1/1999 | 8/23/1999 | 3/11/2013 |  | 8/23/1999 | 5/31/2010 | 1 |
| 4837 | M | 1/1/1995 | 3/9/1995 | 2/24/2000 | 7/3/2000 | 3/9/1995 | 7/3/2000 | 1 |
| 4838 | F | 2/23/1999 | 5/16/1999 | 5/5/2011 |  | 5/16/1999 | 5/31/2010 | 1 |
| 4839 |  | 5/26/2005 | 12/29/2005 | 12/29/2005 | 2/20/2006 | 12/29/2005 | 2/20/2006 | 0 |
| 4840 | F | 4/1/2000 | 11/2/2000 | 3/8/2013 |  | 11/2/2000 | 5/31/2010 | 1 |
| 4841 | M | 8/1/2006 | 11/22/2006 | 3/15/2013 |  | 11/22/2006 | 5/31/2010 | 1 |
| 4843 | F | 4/1/2002 | 10/27/2002 | 11/18/2012 |  | 10/27/2002 | 5/31/2010 | 1 |
| 4844 | F | 5/12/2008 | 8/12/2008 | 11/18/2012 |  | 8/12/2008 | 5/31/2010 | 1 |
| 4847 | M | 4/1/1997 | 4/28/1997 | 7/19/2005 | 3/11/2002 | 4/1/1997 | 3/11/2002 | 1 |
| 4848 | M | 8/1/2005 | 6/2/2006 | 5/28/2011 |  | 6/2/2006 | 5/31/2010 | 1 |
| 4850 | M | 9/20/1991 | 1/17/1993 | 12/27/1999 | 11/1/2000 | 1/17/1993 | 11/1/2000 | 1 |
| 4851 | F | 4/5/2008 | 11/20/2008 | 1/15/2010 | 2/15/2011 | 11/20/2008 | 5/31/2010 | 0 |
| 4855 | M | 11/8/2007 | 1/25/2008 | 11/15/2012 |  | 1/25/2008 | 5/31/2010 | 1 |
| 4858 | M | 7/26/2005 | 12/30/2006 | 12/30/2006 | 12/30/2006 | 12/30/2006 | 12/30/2006 | 0 |
| 4859 | F | 2/20/2009 | 5/12/2009 | 1/15/2013 |  | 5/12/2009 | 5/31/2010 | 1 |
| 4860 | M | 5/29/2009 | 7/19/2009 | 3/11/2013 |  | 5/29/2009 | 5/31/2010 | 1 |
| 4862 | F | 11/7/1997 | 1/13/1998 | 2/10/2001 | 12/9/2001 | 1/13/1998 | 12/9/2001 | 0 |
| 4863 | M | 10/27/2000 | 1/26/2001 | 10/16/2004 |  | 1/26/2001 | 5/31/2010 | 0 |
| 4864 | M | 3/1/2008 | 1/7/2009 | 12/3/2012 |  | 1/7/2009 | 5/31/2010 | 1 |
| 4866 | F | 1/1/2004 | 3/21/2004 | 4/15/2013 |  | 3/21/2004 | 5/31/2010 | 1 |
| 4870 | F | 8/1/2003 | 8/26/2003 | 12/29/2012 |  | 8/1/2003 | 5/31/2010 | 1 |
| 4871 | F | 5/30/2009 | 7/12/2009 | 12/29/2012 |  | 5/30/2009 | 5/31/2010 | 1 |
| 4872 | F | 6/1/2007 | 10/27/2007 | 1/19/2013 |  | 10/27/2007 | 5/31/2010 | 1 |
| 4875 | M | 2/1/2007 | 9/16/2007 | 2/24/2013 |  | 9/16/2007 | 5/31/2010 | 1 |
| 4877 | M | 6/1/2007 | 7/1/2007 | 1/18/2013 |  | 6/1/2007 | 5/31/2010 | 1 |
| 4879 | M | 1/1/2007 | 4/23/2007 | 3/22/2013 |  | 4/23/2007 | 5/31/2010 | 1 |
| 4883 | M | 7/17/2003 | 8/26/2003 | 1/10/2013 |  | 7/17/2003 | 5/31/2010 | 1 |
| 4885 | M | 3/21/2002 | 4/6/2002 | 11/29/2011 | 3/12/2012 | 3/21/2002 | 5/31/2010 | 1 |
| 4889 | F | 6/10/2000 | 6/12/2000 | 1/21/2013 |  | 6/10/2000 | 5/31/2010 | 1 |
| 4890 | F | 4/12/2006 | 6/29/2006 | 1/21/2013 |  | 6/29/2006 | 5/31/2010 | 1 |
| 4891 | F | 6/15/2006 | 7/3/2006 | 3/8/2013 |  | 6/15/2006 | 5/31/2010 | 1 |
| 4892 | F | 4/1/2006 | 4/16/2007 | 1/3/2013 |  | 4/16/2007 | 5/31/2010 | 1 |
| 4895 | F | 6/8/2000 | 6/24/2000 | 3/19/2013 |  | 6/8/2000 | 5/31/2010 | 1 |
| 4896 | M | 2/1/2006 | 5/25/2006 | 3/19/2013 |  | 5/25/2006 | 5/31/2010 | 1 |
| 4900 | F | 1/1/1998 | 6/27/1998 | 1/7/2013 |  | 6/27/1998 | 5/31/2010 | 1 |
| 4901 | F | 12/1/2004 | 12/20/2004 | 1/7/2013 |  | 12/1/2004 | 5/31/2010 | 1 |
| 4903 | F | 11/1/2001 | 6/3/2002 | 1/6/2013 |  | 6/3/2002 | 5/31/2010 | 1 |
| 4904 | M | 5/1/2007 | 7/9/2007 | 1/6/2013 |  | 7/9/2007 | 5/31/2010 | 1 |
| 4905 | F | 12/31/2001 | 10/13/2002 | 10/23/2007 | 6/17/2008 | 10/13/2002 | 6/17/2008 | 1 |
| 4906 | M | 9/8/2002 | 2/5/2003 | 6/8/2006 | 11/25/2006 | 2/5/2003 | 11/25/2006 | 0 |
| 4910 | M | 7/5/2001 | 2/21/2004 | 5/11/2004 | 10/16/2004 | 2/21/2004 | 10/16/2004 | 0 |
| 4912 | F | 3/26/2002 | 5/29/2002 | 5/26/2012 | 8/10/2012 | 5/29/2002 | 5/31/2010 | 1 |
| 4913 | M | 7/1/2008 | 10/10/2008 | 5/26/2012 | 7/31/2012 | 10/10/2008 | 5/31/2010 | 1 |
| 4914 | M | 8/12/2007 | 8/28/2007 | 2/23/2013 |  | 8/12/2007 | 5/31/2010 | 1 |
| 4916 | M | 4/4/1997 | 12/4/1998 | 12/6/1998 | 12/6/1998 | 12/4/1998 | 12/6/1998 | 0 |
| 4919 | F | 4/30/2002 | 5/9/2002 | 10/28/2010 | 12/6/2011 | 4/30/2002 | 5/31/2010 | 1 |
| 4920 | F | 8/28/2004 | 12/15/2004 | 12/18/2011 | 7/1/2012 | 12/15/2004 | 5/31/2010 | 1 |
| 4926 | F | 9/1/1997 | 11/28/1997 | 5/23/2001 | 8/23/2001 | 11/28/1997 | 8/23/2001 | 0 |
| 4929 | F | 7/9/1998 | 11/27/1998 | 12/26/1998 | 3/1/1999 | 11/27/1998 | 3/1/1999 | 0 |
| 4930 | F | 12/1/2005 | 1/3/2006 | 4/9/2012 |  | 12/1/2005 | 5/31/2010 | 1 |
| 4931 | F | 8/31/2000 | 11/1/2000 | 5/13/2010 | 8/15/2010 | 11/1/2000 | 5/31/2010 | 1 |
| 4932 | F | 6/1/2006 | 7/8/2006 | 5/13/2010 | 8/9/2010 | 6/1/2006 | 5/31/2010 | 0 |
| 4933 | M | 3/15/2008 | 5/8/2008 | 3/27/2010 | 10/1/2010 | 3/15/2008 | 5/31/2010 | 0 |
| 4934 | F | 2/1/2006 | 5/2/2006 | 1/4/2013 |  | 5/2/2006 | 5/31/2010 | 1 |
| 4936 | M | 9/1/2008 | 2/28/2009 | 4/15/2013 |  | 2/28/2009 | 5/31/2010 | 1 |
| 4938 | M | 5/11/1999 | 5/19/1999 | 5/19/1999 | 5/21/1999 | 5/11/1999 | 5/21/1999 | 0 |
| 4939 | F | 2/1/2003 | 8/15/2003 | 2/24/2013 |  | 8/15/2003 | 5/31/2010 | 1 |
| 5415 | F | 5/1/1989 | 5/1/1990 | 12/30/1993 | 10/24/1994 | 4/30/1993 | 10/24/1994 | 0 |
| 5538 | F | 11/15/2007 | 3/22/2008 | 12/23/2011 | 3/18/2012 | 3/22/2008 | 5/31/2010 | 1 |
| 5674 | F | 6/1/1995 | 10/30/1995 | 10/30/1995 | 11/8/1997 | 10/30/1995 | 11/8/1997 | 0 |
| 5786 | M | 1/1/1960 | 10/9/1990 | 9/18/1998 | 3/29/1999 | 10/8/1993 | 3/29/1999 | 0 |
| 5829 | F | 1/1/1985 | 2/3/2003 | 3/18/2013 |  | 2/2/2006 | 5/31/2010 | 1 |
| 5831 | M | 2/26/1993 | 4/30/1993 | 4/30/1993 | 4/30/1993 | 4/30/1993 | 4/30/1993 | 0 |
| 5834 | M | 9/7/1995 | 9/23/1995 | 6/5/2000 | 7/14/2003 | 9/7/1995 | 7/14/2003 | 0 |
| 5939 | F | 5/1/1992 | 7/25/1992 | 10/20/1996 | 3/13/1997 | 7/25/1992 | 3/13/1997 | 0 |
