# Supplementary material for: Demography of a forest elephant population
Source: PLoS One. 2018 Feb 15;13(2):e0192777. doi: 10.1371/journal.pone.0192777 (PMC5813957; doi:10.1371/journal.pone.0192777)
Supplement: S3 Table — (DOCX) [file pone.0192777.s004.docx]

**Supporting Information**

**Demography of a Forest Elephant population**

Andrea K. Turkalo, Peter H. Wrege, George Wittemyer

S4 Table. Individual based inter-calf interval data

| momid | mombirth | calf1 | birth1 | sex1 | calf2 | birth2 | sex2 | censored | interval | calf1DieDate |
| --- | --- | --- | --- | --- | --- | --- | --- | --- | --- | --- |
| 4 | 1/1/1935 | 7 | 4/25/1994 | F |  |  |  | 1 | 40 | 10/15/1997 |
| 8 | 1/1/1964 | 15 | 7/1/1997 | F |  |  |  | 1 | 60 | 1/15/2010 |
| 8 | 1/1/1964 | 13 | 12/9/1990 | M | 14 | 4/19/1994 | F | 0 | 40 | 4/2/2002 |
| 22 | 1/1/1969 | 25 | 6/15/1997 | F |  |  |  | 1 | 35 | 4/26/2001 |
| 22 | 1/1/1969 | 24 | 9/3/1991 | F | 25 | 6/15/1997 | F | 0 | 69 |  |
| 52 | 1/1/1967 | 54 | 4/1/2000 | M | 55 | 8/1/2006 | F | 1 | 37 |  |
| 52 | 1/1/1967 | 55 | 8/1/2006 | F | 6002 | 2/1/2012 | M | 0 | 66 |  |
| 69 | 1/10/1960 | 71 | 12/1/1992 | F | 75 | 9/15/1998 | F | 0 | 70 | 4/30/2011 |
| 69 | 1/10/1960 | 75 | 9/15/1998 | F | 76 | 3/1/2003 | M | 0 | 54 |  |
| 69 | 1/10/1960 | 76 | 3/1/2003 | M | 77 | 6/23/2007 | M | 0 | 52 | 4/16/2012 |
| 69 | 1/10/1960 | 77 | 6/23/2007 | M | 5998 | 3/1/2012 | F | 0 | 56 |  |
| 87 | 1/1/1965 | 98 | 1/1/2007 | F |  |  |  | 1 | 71 | 6/15/2012 |
| 87 | 1/1/1965 | 193 | 4/5/1995 | M | 97 | 5/3/2002 | F | 0 | 85 | 5/12/2012 |
| 87 | 1/1/1965 | 97 | 5/3/2002 | F | 98 | 1/1/2007 | F | 0 | 56 |  |
| 90 | 1/1/1983 | 93 | 11/21/2009 | F |  |  |  | 1 | 40 |  |
| 90 | 1/1/1983 | 92 | 7/1/2004 | M | 93 | 11/21/2009 | F | 0 | 65 |  |
| 101 | 1/1/1976 | 103 | 3/16/2000 | M | 105 | 5/3/2002 | F | 0 | 26 | 6/10/2000 |
| 102 | 12/1/1994 | 104 | 4/19/2009 | F |  |  |  | 1 | 48 |  |
| 134 | 1/1/1958 | 143 | 2/1/2000 | F | 144 | 5/8/2006 | F | 0 | 75 |  |
| 145 | 1/1/1968 | 4759 | 6/1/2008 | M |  |  |  | 1 | 28 |  |
| 145 | 1/1/1968 | 153 | 8/1/2005 | M | 4759 | 6/1/2008 | M | 0 | 34 |  |
| 165 | 1/1/1955 | 174 | 8/31/1996 | F | 175 | 6/30/2001 | M | 0 | 58 | 1/11/2012 |
| 165 | 1/1/1955 | 175 | 6/30/2001 | M | 176 | 5/30/2006 | F | 0 | 59 |  |
| 165 | 1/1/1955 | 176 | 5/30/2006 | F | 6004 | 11/1/2012 | F | 0 | 77 |  |
| 191 | 1/1/1977 | 4762 | 5/1/1993 | F |  |  |  | 1 | 41 | 1/31/1997 |
| 196 | 1/1/1982 | 197 | 10/1/1999 | F | 4758 | 8/9/2007 | M | 0 | 94 | 10/25/2012 |
| 204 | 1/1/1968 | 212 | 12/11/1997 | F | 213 | 4/1/2003 | M | 0 | 64 | 9/4/2008 |
| 204 | 1/1/1968 | 213 | 4/1/2003 | M | 214 | 4/30/2007 | M | 0 | 49 | 9/19/2008 |
| 207 | 1/1/1983 | 209 | 12/18/2000 | F |  |  |  | 1 | 78 | 2/6/2009 |
| 264 | 1/1/1964 | 287 | 5/5/2007 | M |  |  |  | 1 | 37 |  |
| 264 | 1/1/1964 | 284 | 2/28/1994 | F | 285 | 10/1/1998 | M | 0 | 55 | 6/21/2012 |
| 264 | 1/1/1964 | 285 | 10/1/1998 | M | 286 | 8/1/2003 | F | 0 | 58 | 7/12/2012 |
| 264 | 1/1/1964 | 286 | 8/1/2003 | F | 287 | 5/5/2007 | M | 0 | 45 |  |
| 266 | 1/1/1975 | 276 | 12/21/1995 | M | 277 | 2/7/2001 | M | 0 | 62 |  |
| 266 | 1/1/1975 | 277 | 2/7/2001 | M | 278 | 1/1/2005 | F | 0 | 47 |  |
| 266 | 1/1/1975 | 278 | 1/1/2005 | F | 279 | 10/26/2007 | F | 0 | 34 |  |
| 266 | 1/1/1975 | 279 | 10/26/2007 | F | 5336 | 7/23/2010 | F | 0 | 33 |  |
| 269 | 1/1/1987 | 274 | 5/14/2007 | F |  |  |  | 1 | 45 | 9/11/2010 |
| 269 | 1/1/1987 | 271 | 6/13/2003 | F | 274 | 5/14/2007 | F | 0 | 47 | 10/5/2010 |
| 311 | 1/1/1968 | 325 | 5/13/2009 | F |  |  |  | 1 | 46 |  |
| 311 | 1/1/1968 | 323 | 6/1/1998 | M | 324 | 11/11/2002 | M | 0 | 53 | 7/13/2012 |
| 311 | 1/1/1968 | 324 | 11/11/2002 | M | 325 | 5/13/2009 | F | 0 | 78 |  |
| 313 | 1/1/1984 | 319 | 6/1/2006 | F |  |  |  | 1 | 55 |  |
| 313 | 1/1/1984 | 321 | 4/25/2002 | F | 319 | 6/1/2006 | F | 0 | 49 |  |
| 314 | 1/1/1990 | 317 | 11/20/2009 | F |  |  |  | 1 | 40 |  |
| 314 | 1/1/1990 | 316 | 8/1/2005 | F | 317 | 11/20/2009 | F | 0 | 52 |  |
| 347 | 1/1/1953 | 355 | 11/7/1997 | M |  |  |  | 1 | 121 | 8/15/2011 |
| 372 | 1/1/1971 | 381 | 3/1/2005 | F |  |  |  | 1 | 36 | 6/5/2008 |
| 463 | 1/1/1974 | 472 | 4/13/2010 | F |  |  |  | 1 | 31 |  |
| 568 | 1/1/1966 | 573 | 6/1/2007 | M |  |  |  | 1 | 60 |  |
| 568 | 1/1/1966 | 570 | 12/21/1999 | M | 573 | 6/1/2007 | M | 0 | 89 | 3/25/2012 |
| 569 | 1/1/1990 | 571 | 6/1/2007 | F |  |  |  | 1 | 60 |  |
| 578 | 1/1/1971 | 583 | 7/1/2007 | F |  |  |  | 1 | 41 | 7/19/2012 |
| 590 | 1/1/1952 | 596 | 2/1/2000 | M |  |  |  | 1 | 121 | 11/5/2010 |
| 611 | 1/1/1968 | 617 | 1/22/2010 | F |  |  |  | 1 | 27 |  |
| 611 | 1/1/1968 | 616 | 7/1/2004 | F | 617 | 1/22/2010 | F | 0 | 67 | 3/22/2011 |
| 613 | 11/8/1993 | 4768 | 7/1/2009 | F |  |  |  | 1 | 41 |  |
| 619 | 1/1/1973 | 624 | 10/1/2009 | F |  |  |  | 1 | 39 |  |
| 619 | 1/1/1973 | 621 | 5/1/1997 | M | 622 | 4/1/2003 | F | 0 | 71 |  |
| 619 | 1/1/1973 | 622 | 4/1/2003 | F | 624 | 10/1/2009 | F | 0 | 78 |  |
| 656 | 1/1/1984 | 660 | 8/1/2003 | F |  |  |  | 1 | 114 |  |
| 670 | 1/1/1964 | 672 | 2/25/2010 | F |  |  |  | 1 | 33 |  |
| 683 | 1/1/1966 | 688 | 1/1/1997 | F |  |  |  | 1 | 43 | 10/19/2000 |
| 713 | 1/1/1970 | 718 | 7/1/2008 | F |  |  |  | 1 | 56 |  |
| 713 | 1/1/1970 | 716 | 1/15/1995 | M | 717 | 1/5/2002 | F | 0 | 84 |  |
| 713 | 1/1/1970 | 717 | 1/5/2002 | F | 718 | 7/1/2008 | F | 0 | 78 |  |
| 736 | 1/1/1972 | 741 | 3/13/2009 | F |  |  |  | 1 | 48 |  |
| 736 | 1/1/1972 | 739 | 6/30/1998 | F | 740 | 1/1/2005 | F | 0 | 78 |  |
| 736 | 1/1/1972 | 740 | 1/1/2005 | F | 741 | 3/13/2009 | F | 0 | 50 |  |
| 773 | 1/1/1970 | 776 | 4/6/2009 | M |  |  |  | 1 | 47 |  |
| 773 | 1/1/1970 | 778 | 4/11/1995 | F | 779 | 8/21/2000 | M | 0 | 64 | 8/13/2010 |
| 799 | 1/1/1983 | 801 | 7/1/2009 | F |  |  |  | 1 | 45 | 8/12/2012 |
| 818 | 1/1/1986 | 4777 | 2/1/2009 | M |  |  |  | 1 | 50 |  |
| 818 | 1/1/1986 | 4775 | 4/1/1999 | F | 4776 | 11/1/2003 | M | 0 | 55 |  |
| 818 | 1/1/1986 | 4776 | 11/1/2003 | M | 4777 | 2/1/2009 | M | 0 | 63 | 8/30/2012 |
| 843 | 1/1/1974 | 4778 | 10/1/2002 | F | 4779 | 8/1/2007 | M | 0 | 58 |  |
| 856 | 1/1/1962 | 4780 | 9/11/1999 | M |  |  |  | 1 | 38 | 4/26/2000 |
| 866 | 1/1/1970 | 882 | 6/1/2009 | M |  |  |  | 1 | 44 |  |
| 866 | 1/1/1970 | 869 | 4/27/1991 | M | 879 | 10/9/1996 | F | 0 | 66 | 1/5/2011 |
| 866 | 1/1/1970 | 879 | 10/9/1996 | F | 880 | 9/28/2000 | M | 0 | 48 | 1/1/2012 |
| 866 | 1/1/1970 | 881 | 9/25/2005 | M | 882 | 6/1/2009 | M | 0 | 44 |  |
| 868 | 1/1/1985 | 4782 | 6/1/2009 | M |  |  |  | 1 | 45 |  |
| 868 | 1/1/1985 | 4781 | 5/1/2003 | M | 4782 | 6/1/2009 | M | 0 | 73 |  |
| 897 | 1/1/1948 | 899 | 5/18/1999 | M |  |  |  | 1 | 48 | 9/17/2003 |
| 929 | 1/1/1971 | 932 | 11/8/1997 | F |  |  |  | 1 | 29 | 6/18/2000 |
| 929 | 1/1/1971 | 931 | 10/24/1992 | M | 932 | 11/8/1997 | F | 0 | 61 | 5/24/2000 |
| 951 | 1/1/1975 | 959 | 11/1/2008 | F |  |  |  | 1 | 49 |  |
| 951 | 1/1/1975 | 958 | 4/1/2004 | M | 959 | 11/1/2008 | F | 0 | 55 |  |
| 952 | 1/1/1991 | 954 | 3/21/2007 | F |  |  |  | 1 | 32 |  |
| 965 | 1/1/1962 | 971 | 6/28/1995 | F | 972 | 6/1/1998 | M | 0 | 35 | 9/18/2006 |
| 981 | 1/1/1966 | 986 | 7/1/2004 | F |  |  |  | 1 | 34 | 9/5/2009 |
| 993 | 1/1/1989 | 995 | 4/4/2008 | F |  |  |  | 1 | 50 |  |
| 1002 | 1/1/1966 | 1005 | 4/28/2004 | F |  |  |  | 1 | 102 |  |
| 1009 | 1/1/1970 | 1014 | 9/3/2009 | M |  |  |  | 1 | 38 |  |
| 1017 | 1/1/1955 | 1030 | 5/28/1999 |  | 1031 | 1/1/2004 | M | 0 | 55 |  |
| 1017 | 1/1/1955 | 1031 | 1/1/2004 |  | 1032 | 5/17/2008 | F | 0 | 53 |  |
| 1017 | 1/1/1955 | 1032 | 5/17/2008 |  | 6008 | 1/25/2013 | M | 0 | 56 |  |
| 1018 | 1/1/1984 | 4788 | 1/24/2006 | F |  |  |  | 1 | 71 |  |
| 1018 | 1/1/1984 | 4787 | 2/23/1999 |  | 4788 | 1/24/2006 | F | 0 | 83 |  |
| 1019 | 1/1/1991 | 4789 | 10/1/2008 | M |  |  |  | 1 | 53 |  |
| 1051 | 1/1/1972 | 1054 | 1/17/2000 | M | 1055 | 12/7/2006 | F | 0 | 83 |  |
| 1051 | 1/1/1972 | 1055 | 12/7/2006 | F | 6009 | 1/1/2013 | F | 1 | 46 |  |
| 1052 | 1/1/1988 | 1053 | 11/1/2002 | F | 6047 | 11/1/2011 | M | 0 | 108 |  |
| 1074 | 1/1/1969 | 1086 | 8/1/2007 | M | 6049 | 1/1/2013 |  | 0 | 65 |  |
| 1075 | 1/1/1973 | 1079 | 7/1/2007 | M |  |  |  | 1 | 68 |  |
| 1075 | 1/1/1973 | 1076 | 1/20/1995 | F | 1077 | 10/16/1999 | F | 0 | 57 |  |
| 1075 | 1/1/1973 | 1077 | 10/16/1999 | F | 1078 | 12/1/2003 | F | 0 | 50 |  |
| 1075 | 1/1/1973 | 1078 | 12/1/2003 | F | 1079 | 7/1/2007 | M | 0 | 43 |  |
| 1098 | 1/1/1984 | 1099 | 6/20/1998 | F |  |  |  | 1 | 62 | 12/14/2003 |
| 1114 | 1/1/1955 | 1116 | 4/29/2000 | M | 1117 | 4/14/2010 | F | 1 | 29 | 1/11/2011 |
| 1127 | 1/1/1970 | 1129 | 11/18/2000 | M | 6052 | 3/3/2012 | F | 0 | 136 |  |
| 1140 | 1/1/1985 | 1141 | 7/3/2009 | M |  |  |  | 1 | 44 |  |
| 1178 | 1/1/1973 | 1181 | 10/1/1998 | M |  |  |  | 1 | 37 | 12/17/2001 |
| 1178 | 1/1/1973 | 1180 | 9/1/1992 | M | 1181 | 10/1/1998 | M | 0 | 73 | 4/27/2005 |
| 1186 | 1/1/1967 | 1194 | 10/1/2007 | M |  |  |  | 1 | 64 |  |
| 1186 | 1/1/1967 | 1192 | 11/1/1999 | F | 1193 | 7/1/2003 | M | 0 | 44 |  |
| 1186 | 1/1/1967 | 1193 | 7/1/2003 | M | 1194 | 10/1/2007 | M | 0 | 51 |  |
| 1188 | 1/1/1986 | 1189 | 9/2/2005 | F | 5992 | 1/1/2012 | M | 0 | 76 |  |
| 1216 | 1/1/1955 | 1220 | 6/7/2000 | M |  |  |  | 1 | 36 | 1/8/2004 |
| 1216 | 1/1/1955 | 1219 | 5/6/1998 | M | 1220 | 6/7/2000 | M | 0 | 25 | 6/5/2005 |
| 1241 | 1/1/1968 | 1242 | 4/30/1996 | F | 1243 | 6/1/2000 | F | 0 | 49 |  |
| 1248 | 1/1/1957 | 1276 | 12/17/2000 | F | 1277 | 10/29/2007 | F | 0 | 82 |  |
| 1287 | 1/1/1976 | 1289 | 7/15/1992 | F | 1290 | 3/10/1997 | F | 0 | 56 |  |
| 1287 | 1/1/1976 | 1290 | 3/10/1997 | F | 1292 | 12/1/2001 | F | 0 | 57 |  |
| 1287 | 1/1/1976 | 1292 | 12/1/2001 | F | 1293 | 5/30/2006 | M | 0 | 54 |  |
| 1287 | 1/1/1976 | 1293 | 5/30/2006 | M | 5794 | 2/15/2012 | M | 0 | 69 | 7/14/2012 |
| 1317 | 1/1/1980 | 1318 | 1/8/2002 | F |  |  |  | 1 | 73 | 10/13/2012 |
| 1319 | 1/1/1988 | 1321 | 4/1/2007 | M |  |  |  | 1 | 71 |  |
| 1319 | 1/1/1988 | 1320 | 6/13/2003 | M | 1321 | 4/1/2007 | M | 0 | 46 |  |
| 1347 | 1/1/1976 | 1350 | 10/13/2008 | F |  |  |  | 1 | 30 |  |
| 1347 | 1/1/1976 | 1349 | 8/27/2004 | M | 1350 | 10/13/2008 | F | 0 | 50 |  |
| 1361 | 1/1/1970 | 1364 | 5/1/2000 | M |  |  |  | 1 | 24 | 12/4/2002 |
| 1396 | 1/1/1977 | 1401 | 4/1/2008 | M |  |  |  | 1 | 56 |  |
| 1397 | 1/1/1972 | 1399 | 7/1/1998 | F |  |  |  | 1 | 58 | 11/8/2003 |
| 1414 | 1/1/1968 | 1419 | 10/1/2008 | F |  |  |  | 1 | 52 |  |
| 1414 | 1/1/1968 | 1418 | 12/1/2000 | M | 1419 | 10/1/2008 | F | 0 | 94 |  |
| 1421 | 1/1/1992 | 1422 | 7/22/2008 | M |  |  |  | 1 | 28 | 3/28/2011 |
| 1431 | 1/1/1955 | 1434 | 12/1/2006 | M |  |  |  | 1 | 32 | 4/12/2010 |
| 1439 | 1/1/1964 | 1449 | 12/15/2004 | F |  |  |  | 1 | 96 |  |
| 1441 | 1/1/1983 | 4805 | 9/8/2004 | F | 5635 | 11/17/2010 | U | 0 | 74 |  |
| 1465 | 1/1/1971 | 1467 | 8/1/2000 | F | 1468 | 1/1/2006 | F | 0 | 65 |  |
| 1465 | 1/1/1971 | 1468 | 1/1/2006 | F | 5781 | 7/18/2010 | F | 0 | 55 |  |
| 1508 | 1/1/1976 | 1509 | 4/17/1999 | F |  |  |  | 1 | 48 | 7/11/2003 |
| 1528 | 1/1/1941 | 1529 | 7/8/1996 | M |  |  |  | 1 | 73 | 12/28/2002 |
| 1565 | 1/1/1978 | 1566 | 4/6/1997 | F | 1567 | 4/15/2002 | F | 0 | 60 |  |
| 1565 | 1/1/1978 | 1567 | 4/15/2002 | F | 1568 | 9/1/2007 | M | 0 | 65 |  |
| 1570 | 1/1/1955 | 1579 | 5/15/2008 | M |  |  |  | 1 | 48 | 10/14/2012 |
| 1570 | 1/1/1955 | 1578 | 11/21/2002 | F | 1579 | 5/15/2008 | M | 0 | 66 |  |
| 1571 | 1/1/1970 | 1574 | 6/1/2009 | F |  |  |  | 1 | 46 |  |
| 1576 | 1/4/1993 | 5461 | 5/1/2010 | F |  |  |  | 1 | 35 |  |
| 1597 | 1/1/1987 | 4812 | 9/1/2005 | M |  |  |  | 1 | 31 |  |
| 1673 | 1/1/1960 | 1678 | 6/27/2004 | F |  |  |  | 1 | 103 |  |
| 1674 | 1/1/1989 | 4815 | 9/1/2007 | M |  |  |  | 1 | 64 |  |
| 1725 | 1/1/1976 | 1727 | 11/19/2000 | F |  |  |  | 1 | 33 | 11/24/2003 |
| 1729 | 1/1/1960 | 4818 | 1/26/1995 | F |  |  |  | 1 | 178 | 8/23/1995 |
| 1729 | 1/1/1960 | 1731 | 3/1/1993 | F | 4818 | 1/26/1995 | F | 0 | 23 | 3/2/2006 |
| 1764 | 1/1/1959 | 1768 | 5/3/2001 | F |  |  |  | 1 | 29 | 2/9/2004 |
| 1764 | 1/1/1959 | 1767 | 1/1/1997 | M | 1768 | 5/3/2001 | F | 0 | 52 | 1/10/2004 |
| 1772 | 1/1/1973 | 1775 | 6/26/1996 | F | 1776 | 11/8/2002 | M | 0 | 77 |  |
| 1772 | 1/1/1973 | 1776 | 11/8/2002 | M | 1777 | 5/1/2007 | M | 0 | 54 |  |
| 1772 | 1/1/1973 | 1777 | 5/1/2007 | M | 6013 | 8/1/2012 | M | 0 | 63 | 7/23/2012 |
| 1779 | 1/1/1960 | 1789 | 4/1/1999 | F | 1790 | 8/1/2002 | F | 0 | 40 |  |
| 1825 | 1/1/1963 | 1832 | 9/1/1999 | M | 1833 | 6/6/2004 | F | 0 | 57 | 5/24/2011 |
| 1829 | 1/1/1990 | 1831 | 4/1/2007 | M |  |  |  | 1 | 36 |  |
| 1839 | 1/1/1952 | 5834 | 9/7/1995 | M |  |  |  | 1 | 24 | 7/14/2003 |
| 1893 | 1/1/1957 | 1896 | 4/23/2006 | M |  |  |  | 1 | 79 | 10/8/2006 |
| 1906 | 1/1/1964 | 1910 | 3/1/2001 | F | 1911 | 5/1/2003 | M | 0 | 26 |  |
| 1906 | 1/1/1964 | 1911 | 5/1/2003 | M | 1912 | 5/13/2006 | M | 0 | 36 | 2/14/2012 |
| 1906 | 1/1/1964 | 1912 | 5/13/2006 | M | 6059 | 7/4/2012 | M | 0 | 74 |  |
| 1913 | 1/1/1969 | 1918 | 11/1/1996 | F | 1919 | 9/1/2001 | M | 0 | 58 | 6/8/2011 |
| 1913 | 1/1/1969 | 1919 | 9/1/2001 | M | 1920 | 2/1/2006 | F | 0 | 53 |  |
| 1913 | 1/1/1969 | 1920 | 2/1/2006 | F | 6060 | 4/1/2012 |  | 0 | 74 |  |
| 1921 | 1/1/1965 | 1929 | 7/22/2008 | M |  |  |  | 1 | 30 | 6/26/2011 |
| 1921 | 1/1/1965 | 1928 | 11/25/2004 | M | 1929 | 7/22/2008 | M | 0 | 44 | 7/2/2011 |
| 1922 | 1/1/1985 | 1923 | 1/18/2007 | F |  |  |  | 1 | 72 |  |
| 1922 | 1/1/1985 | 4826 | 6/1/2000 | M | 1923 | 1/18/2007 | F | 0 | 80 |  |
| 1938 | 1/1/1968 | 1941 | 9/30/1998 | M |  |  |  | 1 | 31 | 7/10/2001 |
| 1938 | 1/1/1968 | 1940 | 2/1/1993 | M | 1941 | 9/30/1998 | M | 0 | 68 | 7/26/2001 |
| 1945 | 1/1/1971 | 1948 | 1/1/2006 | M |  |  |  | 1 | 25 |  |
| 1975 | 1/1/1970 | 1978 | 3/18/1996 | F | 1982 | 6/4/2000 | F | 0 | 51 |  |
| 1975 | 1/1/1970 | 1982 | 6/4/2000 | F | 1983 | 6/30/2004 | M | 0 | 49 | 3/26/2012 |
| 1975 | 1/1/1970 | 1983 | 6/30/2004 | M | 1984 | 5/1/2008 | M | 0 | 46 | 4/7/2012 |
| 1975 | 1/1/1970 | 1984 | 5/1/2008 | M | 6014 | 6/1/2012 | F | 0 | 49 |  |
| 1977 | 1/1/1986 | 4827 | 10/3/2007 | M |  |  |  | 1 | 24 |  |
| 1999 | 1/1/1966 | 2003 | 4/28/1997 | M |  |  |  | 1 | 51 |  |
| 2011 | 1/1/1951 | 2012 | 2/1/1994 | M |  |  |  | 1 | 88 | 3/27/2004 |
| 2038 | 1/1/1982 | 4830 | 10/1/2008 | M |  |  |  | 1 | 49 |  |
| 2038 | 1/1/1982 | 4828 | 4/8/1999 | F | 4829 | 1/1/2004 | F | 0 | 57 |  |
| 2038 | 1/1/1982 | 4829 | 1/1/2004 | F | 4830 | 10/1/2008 | M | 0 | 57 |  |
| 2071 | 1/1/1960 | 2084 | 8/19/1998 | F | 2085 | 9/1/2004 | F | 0 | 73 |  |
| 2071 | 1/1/1960 | 2085 | 9/1/2004 | F | 6077 | 11/1/2011 | M | 0 | 86 |  |
| 2073 | 6/1/1990 | 2075 | 2/1/2007 | F |  |  |  | 1 | 73 |  |
| 2090 | 1/1/1970 | 2096 | 2/9/2010 | F |  |  |  | 1 | 37 |  |
| 2090 | 1/1/1970 | 2092 | 2/1/1999 | F | 2095 | 3/1/2005 | M | 1 | 45 |  |
| 2090 | 1/1/1970 | 2095 | 3/1/2005 | M | 2096 | 2/9/2010 | F | 0 | 59 | 6/29/2012 |
| 2106 | 1/1/1960 | 2111 | 4/1/1998 | M |  |  |  | 1 | 48 |  |
| 2125 | 1/1/1961 | 2134 | 8/25/1999 | F | 2135 | 7/25/2003 | F | 0 | 47 | 8/1/2009 |
| 2223 | 1/1/1962 | 2235 | 8/15/1996 | M | 2237 | 7/13/2000 | F | 0 | 47 | 1/14/2012 |
| 2223 | 1/1/1962 | 2237 | 7/13/2000 | F | 2238 | 5/1/2003 | F | 0 | 34 |  |
| 2223 | 1/1/1962 | 2238 | 5/1/2003 | F | 2239 | 3/25/2008 | M | 0 | 59 | 10/15/2012 |
| 2224 | 1/1/1967 | 2228 | 3/27/2006 | M |  |  |  | 1 | 37 | 7/20/2009 |
| 2230 | 1/1/1991 | 4834 | 4/1/2006 | F | 5291 | 12/11/2010 | M | 0 | 56 |  |
| 2241 | 1/1/1960 | 2243 | 4/1/1995 | F | 2244 | 10/13/1999 | F | 0 | 54 |  |
| 2248 | 1/1/1969 | 2269 | 10/25/2008 | F |  |  |  | 1 | 53 |  |
| 2248 | 1/1/1969 | 2264 | 3/28/1991 | M | 2266 | 7/2/1995 | M | 0 | 51 | 6/6/2002 |
| 2248 | 1/1/1969 | 2266 | 7/2/1995 | M | 2267 | 10/28/1999 | F | 0 | 52 |  |
| 2248 | 1/1/1969 | 2268 | 7/8/2003 | F | 2269 | 10/25/2008 | F | 0 | 64 |  |
| 2249 | 1/1/1984 | 2251 | 10/31/2008 | M |  |  |  | 1 | 52 |  |
| 2249 | 1/1/1984 | 4836 | 6/1/1999 | M | 2250 | 9/19/2005 | M | 0 | 76 |  |
| 2249 | 1/1/1984 | 2250 | 9/19/2005 | M | 2251 | 10/31/2008 | M | 0 | 37 |  |
| 2291 | 1/1/1971 | 2296 | 6/1/2006 | M |  |  |  | 1 | 81 |  |
| 2291 | 1/1/1971 | 2295 | 11/2/1999 | M | 2296 | 6/1/2006 | M | 0 | 79 |  |
| 2311 | 1/1/1955 | 2317 | 2/3/1991 | M | 2318 | 5/24/1995 | F | 0 | 52 | 9/19/2002 |
| 2319 | 1/1/1983 | 2321 | 5/1/2007 | F |  |  |  | 1 | 70 |  |
| 2330 | 1/1/1980 | 2336 | 12/13/2008 | M | 5295 | 10/1/2010 | F | 0 | 22 |  |
| 2353 | 1/1/1960 | 2363 | 10/1/2006 | M |  |  |  | 1 | 76 |  |
| 2355 | 1/1/1979 | 4840 | 4/1/2000 | F | 4841 | 8/1/2006 | M | 0 | 76 |  |
| 2355 | 1/1/1979 | 4841 | 8/1/2006 | M | 6017 | 6/1/2012 | M | 0 | 70 |  |
| 2374 | 1/1/1973 | 2376 | 2/27/1999 | F | 2378 | 3/1/2003 | M | 0 | 48 |  |
| 2405 | 1/1/1966 | 2416 | 12/28/2007 | M |  |  |  | 1 | 59 |  |
| 2405 | 1/1/1966 | 2414 | 7/1/1999 | F | 2415 | 6/1/2003 | M | 0 | 47 |  |
| 2405 | 1/1/1966 | 2415 | 6/1/2003 | M | 2416 | 12/28/2007 | M | 0 | 55 |  |
| 2408 | 1/1/1986 | 4844 | 5/12/2008 | F |  |  |  | 1 | 54 |  |
| 2408 | 1/1/1986 | 4843 | 4/1/2002 | F | 4844 | 5/12/2008 | F | 0 | 73 |  |
| 2418 | 1/1/1964 | 2420 | 4/8/1994 | F |  |  |  | 1 | 60 |  |
| 2494 | 1/1/1973 | 5300 | 4/1/2010 | M |  |  |  | 1 | 37 |  |
| 2516 | 1/1/1966 | 4846 | 7/5/2009 | M |  |  |  | 1 | 35 |  |
| 2516 | 1/1/1966 | 2526 | 4/15/2001 | M | 4846 | 7/5/2009 | M | 1 | 26 |  |
| 2537 | 1/1/1975 | 2539 | 3/31/2002 | F |  |  |  | 1 | 66 |  |
| 2564 | 1/1/1963 | 2568 | 7/1/2003 | M |  |  |  | 1 | 73 | 2/26/2010 |
| 2578 | 1/1/1975 | 2579 | 9/1/1999 | M | 2580 | 2/1/2007 | F | 0 | 89 |  |
| 2578 | 1/1/1975 | 2580 | 2/1/2007 | F | 2581 | 7/1/2012 | F | 0 | 65 |  |
| 2620 | 1/1/1964 | 2622 | 1/10/1998 | F |  |  |  | 1 | 54 | 10/21/2002 |
| 2634 | 1/1/1963 | 2636 | 10/28/1991 | F |  |  |  | 1 | 90 | 8/22/1999 |
| 2713 | 1/1/1951 | 2718 | 2/15/1996 | F | 2719 | 11/8/2002 | F | 0 | 81 | 9/18/2007 |
| 2723 | 1/1/1975 | 2729 | 5/1/2007 | M |  |  |  | 1 | 69 |  |
| 2723 | 1/1/1975 | 2725 | 5/1/2002 | F | 2729 | 5/1/2007 | M | 0 | 60 |  |
| 2724 | 1/1/1994 | 4859 | 2/20/2009 | F |  |  |  | 1 | 47 |  |
| 2732 | 1/1/1964 | 2734 | 3/1/1997 | F | 2736 | 7/29/2002 | M | 0 | 65 |  |
| 2732 | 1/1/1964 | 2736 | 7/29/2002 | M | 2737 | 2/15/2007 | M | 0 | 55 |  |
| 2732 | 1/1/1964 | 2737 | 2/15/2007 | M | 5997 | 7/1/2012 | F | 0 | 65 |  |
| 2733 | 8/1/1992 | 4860 | 5/29/2009 | M |  |  |  | 1 | 45 |  |
| 2791 | 1/1/1960 | 2798 | 5/15/2007 | F |  |  |  | 1 | 69 |  |
| 2791 | 1/1/1960 | 2795 | 9/30/1993 | M | 2796 | 3/1/1999 | M | 0 | 65 | 1/18/2007 |
| 2791 | 1/1/1960 | 2796 | 3/1/1999 | M | 2797 | 4/1/2003 | F | 0 | 49 | 6/5/2010 |
| 2791 | 1/1/1960 | 2797 | 4/1/2003 | F | 2798 | 5/15/2007 | F | 0 | 50 |  |
| 2792 | 1/1/1994 | 2793 | 5/1/2009 | F |  |  |  | 1 | 41 |  |
| 2821 | 1/1/1991 | 4864 | 3/1/2008 | M |  |  |  | 1 | 57 |  |
| 2834 | 1/1/1963 | 2848 | 4/1/2002 | F |  |  |  | 1 | 127 |  |
| 2834 | 1/1/1963 | 2847 | 4/6/1996 |  | 2848 | 4/1/2002 | F | 0 | 72 |  |
| 2841 | 1/1/1980 | 2843 | 10/1/2009 |  |  |  |  | 1 | 36 |  |
| 2841 | 1/1/1980 | 2842 | 10/1/2002 |  | 2843 | 10/1/2009 |  | 0 | 84 |  |
| 2909 | 1/1/1958 | 2919 | 12/29/2009 | M |  |  |  | 1 | 40 |  |
| 2909 | 1/1/1958 | 2917 | 4/5/2003 | F | 2919 | 12/29/2009 | M | 0 | 81 |  |
| 2925 | 1/1/1966 | 5308 | 4/25/2009 | F |  |  |  | 1 | 43 |  |
| 2952 | 1/1/1960 | 2961 | 8/1/2001 | F |  |  |  | 1 | 138 |  |
| 2953 | 1/1/1981 | 4871 | 5/30/2009 | F |  |  |  | 1 | 43 |  |
| 2953 | 1/1/1981 | 4870 | 8/1/2003 | F | 4871 | 5/30/2009 | F | 0 | 70 |  |
| 2991 | 1/1/1991 | 2994 | 12/6/2009 | M |  |  |  | 1 | 40 |  |
| 3000 | 1/1/1985 | 3017 | 10/13/2001 | M | 4875 | 2/1/2007 | M | 0 | 64 |  |
| 3000 | 1/1/1985 | 4875 | 2/1/2007 | M | 5994 | 3/1/2012 | M | 0 | 61 |  |
| 3026 | 1/1/1973 | 3034 | 6/1/1997 | F | 3035 | 8/1/2002 | M | 0 | 62 |  |
| 3026 | 1/1/1973 | 3035 | 8/1/2002 | M | 3036 | 2/1/2007 | M | 0 | 54 | 7/7/2011 |
| 3026 | 1/1/1973 | 3036 | 2/1/2007 | M | 5999 | 3/1/2012 | M | 0 | 61 |  |
| 3029 | 1/1/1989 | 4877 | 6/1/2007 | M |  |  |  | 1 | 68 |  |
| 3048 | 1/1/1970 | 3059 | 8/15/2001 | F |  |  |  | 1 | 139 |  |
| 3048 | 1/1/1970 | 3058 | 8/1/1999 | F | 3059 | 8/15/2001 | F | 0 | 25 |  |
| 3054 | 1/1/1987 | 4879 | 1/1/2007 | F | 6025 | 12/1/2012 | M | 0 | 71 |  |
| 3080 | 1/1/1963 | 3083 | 1/12/2000 | M |  |  |  | 1 | 77 | 5/22/2007 |
| 3095 | 1/1/1976 | 3103 | 6/10/2006 | F |  |  |  | 1 | 81 |  |
| 3199 | 1/1/1968 | 3211 | 1/1/2010 | F |  |  |  | 1 | 39 |  |
| 3199 | 1/1/1968 | 3209 | 9/1/1999 | F | 3210 | 6/22/2005 | M | 0 | 70 |  |
| 3199 | 1/1/1968 | 3210 | 6/22/2005 | M | 3211 | 1/1/2010 | F | 0 | 54 |  |
| 3202 | 1/1/1980 | 4883 | 7/17/2003 | M |  |  |  | 1 | 78 |  |
| 3237 | 1/1/1946 | 3247 | 4/1/2002 | F |  |  |  | 1 | 53 | 10/31/2006 |
| 3237 | 1/1/1946 | 3246 | 6/24/1996 | F | 3247 | 4/1/2002 | F | 0 | 69 | 8/13/2006 |
| 3239 | 1/1/1982 | 4885 | 3/21/2002 | F | 5310 | 5/1/2011 | F | 0 | 109 | 3/12/2012 |
| 3329 | 1/1/1959 | 3334 | 9/25/2006 | M |  |  |  | 1 | 73 | 8/8/2010 |
| 3337 | 1/1/1961 | 3340 | 5/4/1991 | F | 3343 | 3/12/1995 | M | 0 | 46 | 7/29/2011 |
| 3337 | 1/1/1961 | 3343 | 3/12/1995 | M | 3344 | 1/1/1999 | F | 0 | 46 |  |
| 3337 | 1/1/1961 | 3344 | 1/1/1999 | F | 3345 | 1/1/2002 | F | 0 | 36 |  |
| 3337 | 1/1/1961 | 3345 | 1/1/2002 | F | 6001 | 11/1/2011 | M | 1 | 79 |  |
| 3340 | 5/4/1991 | 3341 | 5/1/2006 | M |  |  |  | 1 | 27 |  |
| 3357 | 1/1/1965 | 3363 | 12/1/2009 | F |  |  |  | 1 | 38 |  |
| 3357 | 1/1/1965 | 3362 | 7/1/2002 | M | 3363 | 12/1/2009 | F | 1 | 31 |  |
| 3366 | 1/1/1987 | 3371 | 4/1/2006 | F |  |  |  | 1 | 26 |  |
| 3372 | 1/1/1961 | 4888 | 6/20/2009 | M |  |  |  | 1 | 43 |  |
| 3372 | 1/1/1961 | 3382 | 10/10/1994 | F | 3383 | 4/1/1999 | M | 0 | 54 | 5/3/2012 |
| 3372 | 1/1/1961 | 3383 | 4/1/1999 | M | 3384 | 3/31/2002 | F | 0 | 36 | 9/28/2008 |
| 3372 | 1/1/1961 | 3384 | 3/31/2002 | F | 3385 | 9/1/2006 | M | 0 | 53 |  |
| 3372 | 1/1/1961 | 3385 | 9/1/2006 | M | 4888 | 6/20/2009 | M | 0 | 34 |  |
| 3373 | 1/1/1980 | 4890 | 4/12/2006 | F |  |  |  | 1 | 81 |  |
| 3373 | 1/1/1980 | 4889 | 6/10/2000 | F | 4890 | 4/12/2006 | F | 0 | 70 |  |
| 3374 | 1/1/1985 | 4891 | 6/15/2006 | F | 5314 | 9/1/2010 | F | 0 | 51 |  |
| 3382 | 10/10/1994 | 5315 | 1/8/2010 | M |  |  |  | 1 | 23 |  |
| 3387 | 1/1/1974 | 4892 | 4/1/2006 | F |  |  |  | 1 | 81 |  |
| 3387 | 1/1/1974 | 3394 | 4/1/2002 | M | 4892 | 4/1/2006 | F | 0 | 48 |  |
| 3388 | 1/1/1968 | 3391 | 1/1/2007 | M |  |  |  | 1 | 70 |  |
| 3388 | 1/1/1968 | 3390 | 9/9/2004 | F | 3391 | 1/1/2007 | M | 0 | 28 |  |
| 3398 | 11/1/1994 | 4893 | 7/18/2008 | M |  |  |  | 1 | 52 |  |
| 3410 | 1/1/1965 | 3416 | 1/15/2009 | M |  |  |  | 1 | 48 |  |
| 3410 | 1/1/1965 | 3414 | 4/8/1997 | F | 3415 | 7/17/2003 | F | 0 | 75 |  |
| 3410 | 1/1/1965 | 3415 | 7/17/2003 | F | 3416 | 1/15/2009 | M | 0 | 66 |  |
| 3427 | 1/1/1970 | 3437 | 5/20/2007 | M |  |  |  | 1 | 69 |  |
| 3427 | 1/1/1970 | 3429 | 7/1/1993 | M | 3436 | 7/19/1999 | F | 0 | 73 | 9/29/2007 |
| 3427 | 1/1/1970 | 3436 | 7/19/1999 | F | 3437 | 5/20/2007 | M | 0 | 94 |  |
| 3428 | 1/1/1985 | 4895 | 6/8/2000 | F | 4896 | 2/1/2006 | M | 0 | 68 |  |
| 3428 | 1/1/1985 | 4896 | 2/1/2006 | M | 5317 | 10/1/2010 | F | 0 | 56 |  |
| 3439 | 1/1/1965 | 3447 | 5/14/2004 | F |  |  |  | 1 | 63 | 6/30/2012 |
| 3440 | 1/1/1986 | 3441 | 4/1/2000 | F |  |  |  | 1 | 154 |  |
| 3498 | 1/1/1970 | 3501 | 7/30/1994 | F |  |  |  | 1 | 30 | 11/4/1997 |
| 3550 | 1/1/1964 | 3568 | 5/1/2003 | M | 5320 | 10/1/2010 | F | 0 | 89 |  |
| 3552 | 1/1/1979 | 4900 | 1/1/1998 | F | 4901 | 12/1/2004 | F | 0 | 83 |  |
| 3553 | 1/1/1983 | 4904 | 5/1/2007 | M |  |  |  | 1 | 68 |  |
| 3553 | 1/1/1983 | 4903 | 11/1/2001 | F | 4904 | 5/1/2007 | M | 0 | 66 |  |
| 3575 | 1/1/1959 | 3582 | 7/1/1997 | F | 3583 | 8/23/2005 | M | 0 | 98 | 7/16/2008 |
| 3608 | 1/1/1973 | 3612 | 2/20/2006 | F |  |  |  | 1 | 24 | 1/7/2010 |
| 3608 | 1/1/1973 | 3611 | 1/1/2000 | M | 3612 | 2/20/2006 | F | 0 | 74 |  |
| 3648 | 1/1/1961 | 3651 | 3/1/2007 | F | 6097 | 1/1/2012 | M | 0 | 58 |  |
| 3713 | 1/1/1989 | 4913 | 7/1/2008 | M |  |  |  | 1 | 47 | 7/31/2012 |
| 3713 | 1/1/1989 | 4912 | 3/26/2002 | F | 4913 | 7/1/2008 | M | 0 | 75 | 8/10/2012 |
| 3736 | 1/1/1968 | 3750 | 7/15/1999 | M |  |  |  | 1 | 63 | 1/12/2004 |
| 3736 | 1/1/1968 | 3748 | 10/9/1994 | F | 3750 | 7/15/1999 | M | 0 | 57 |  |
| 3738 | 1/1/1973 | 3745 | 6/1/2008 | F |  |  |  | 1 | 56 |  |
| 3738 | 1/1/1973 | 3743 | 4/23/2003 | F | 3745 | 6/1/2008 | F | 0 | 61 |  |
| 3758 | 1/1/1985 | 4914 | 8/12/2007 | M | 5995 | 7/1/2012 | M | 0 | 59 |  |
| 3769 | 1/1/1975 | 3771 | 11/27/2009 | F |  |  |  | 1 | 40 |  |
| 3769 | 1/1/1975 | 3770 | 8/1/2000 | F | 3771 | 11/27/2009 | F | 0 | 112 |  |
| 3817 | 1/1/1955 | 3820 | 6/1/1994 | F | 3821 | 4/1/2001 | M | 0 | 82 |  |
| 3817 | 1/1/1955 | 3821 | 4/1/2001 | M | 3822 | 9/10/2006 | F | 1 | 41 |  |
| 3817 | 1/1/1955 | 3822 | 9/10/2006 | F | 6072 | 12/11/2011 | M | 0 | 63 |  |
| 3831 | 1/1/1958 | 3837 | 10/1/2006 | F |  |  |  | 1 | 44 | 9/8/2010 |
| 3831 | 1/1/1958 | 3834 | 8/4/1996 | F | 3835 | 5/6/2000 | M | 0 | 45 | 8/9/2010 |
| 3831 | 1/1/1958 | 3835 | 5/6/2000 | M | 3836 | 8/1/2003 | M | 0 | 39 | 9/26/2010 |
| 3831 | 1/1/1958 | 3836 | 8/1/2003 | M | 3837 | 10/1/2006 | F | 0 | 38 | 8/25/2010 |
| 3870 | 1/1/1964 | 3883 | 1/29/1997 | F | 3885 | 4/1/2006 | F | 0 | 110 |  |
| 3870 | 1/1/1964 | 3885 | 4/1/2006 | F | 5324 | 7/30/2010 | U | 0 | 52 |  |
| 3898 | 1/1/1965 | 3930 | 1/25/2010 | M |  |  |  | 1 | 37 |  |
| 3898 | 1/1/1965 | 3928 | 6/20/1999 | F | 3929 | 7/1/2003 | M | 0 | 48 |  |
| 3899 | 1/1/1987 | 3900 | 4/1/2007 | F |  |  |  | 1 | 72 |  |
| 3904 | 1/1/1980 | 3906 | 7/18/2002 | F | 3907 | 5/22/2008 | M | 0 | 70 |  |
| 3904 | 1/1/1980 | 3907 | 5/22/2008 | M | 6022 | 10/7/2012 | M | 0 | 53 |  |
| 3913 | 1/1/1959 | 3925 | 11/28/2001 | M |  |  |  | 1 | 93 |  |
| 3917 | 1/1/1985 | 4923 | 8/18/2003 | M |  |  |  | 1 | 113 |  |
| 3917 | 1/1/1985 | 4924 | 4/1/1999 |  | 4923 | 8/18/2003 | M | 0 | 53 |  |
| 3969 | 1/1/1954 | 3975 | 10/2/2000 | M |  |  |  | 1 | 41 | 9/18/2004 |
| 3986 | 1/1/1958 | 3990 | 3/1/1996 | F |  |  |  | 1 | 75 | 7/27/2003 |
| 4031 | 1/1/1961 | 4038 | 9/1/2009 | F |  |  |  | 1 | 39 |  |
| 4031 | 1/1/1961 | 4033 | 9/1/1991 | F | 4036 | 4/1/1998 | F | 0 | 79 |  |
| 4031 | 1/1/1961 | 4036 | 4/1/1998 | F | 4037 | 5/1/2003 | M | 0 | 61 |  |
| 4031 | 1/1/1961 | 4037 | 5/1/2003 | M | 4038 | 9/1/2009 | F | 0 | 76 |  |
| 4033 | 9/1/1992 | 4930 | 12/1/2005 | F | 6099 | 4/1/2011 | F | 1 | 35 |  |
| 4050 | 1/1/1966 | 4056 | 1/10/1993 | F |  |  |  | 1 | 76 | 7/7/2010 |
| 4054 | 1/1/1985 | 4932 | 6/1/2006 | F |  |  |  | 1 | 47 | 8/9/2010 |
| 4056 | 1/10/1993 | 4933 | 3/15/2008 | M |  |  |  | 1 | 24 | 10/1/2010 |
| 4070 | 1/1/1965 | 4074 | 7/14/1999 | F | 4075 | 4/1/2006 | M | 0 | 81 |  |
| 4070 | 1/1/1965 | 4075 | 4/1/2006 | M | 4076 | 5/18/2010 | F | 0 | 50 |  |
| 4070 | 1/1/1965 | 4076 | 5/18/2010 | F | 6000 | 7/1/2012 | M | 0 | 25 | 2/14/2011 |
| 4083 | 1/1/1980 | 4084 | 5/20/2009 | M |  |  |  | 1 | 47 |  |
| 4083 | 1/1/1980 | 4756 | 3/15/2004 | F | 4084 | 5/20/2009 | M | 0 | 62 |  |
| 4087 | 1/1/1963 | 4094 | 4/1/2007 | M |  |  |  | 1 | 73 |  |
| 4087 | 1/1/1963 | 4089 | 2/1/1998 | M | 4094 | 4/1/2007 | M | 0 | 110 | 4/30/2007 |
| 4088 | 1/1/1986 | 4934 | 2/1/2006 | F |  |  |  | 1 | 83 |  |
| 4099 | 1/1/1959 | 4103 | 11/1/1992 | M | 4104 | 5/8/1996 | F | 0 | 42 | 4/5/2003 |
| 4099 | 1/1/1959 | 4104 | 5/8/1996 | F | 4105 | 5/1/2002 | M | 0 | 72 |  |
| 4112 | 1/1/1965 | 4117 | 4/1/2003 | M |  |  |  | 1 | 60 |  |
| 4112 | 1/1/1965 | 4116 | 4/3/1997 | M | 4117 | 4/1/2003 | M | 0 | 72 |  |
| 4148 | 1/1/1958 | 4154 | 6/1/2003 | F |  |  |  | 1 | 72 | 5/25/2009 |
| 4149 | 1/1/1983 | 4150 | 3/1/1998 | F |  |  |  | 1 | 68 | 3/24/2007 |
| 4157 | 1/1/1985 | 4159 | 12/1/2004 | M |  |  |  | 1 | 43 | 1/25/2010 |
| 4157 | 1/1/1985 | 4158 | 10/10/1999 | M | 4159 | 12/1/2004 | M | 0 | 62 | 1/23/2010 |
| 4169 | 1/1/1971 | 4171 | 6/1/2002 | M |  |  |  | 1 | 130 |  |
| 4184 | 1/1/1986 | 4936 | 9/1/2008 | M |  |  |  | 1 | 55 |  |
| 4184 | 1/1/1986 | 4193 | 6/27/2003 | M | 4936 | 9/1/2008 | M | 0 | 62 |  |
| 4185 | 1/1/1985 | 4187 | 3/1/2008 | F |  |  |  | 1 | 61 |  |
| 4185 | 1/1/1985 | 4186 | 8/1/2002 | F | 4187 | 3/1/2008 | F | 0 | 67 |  |
| 4188 | 5/1/1988 | 4190 | 3/10/2009 | F |  |  |  | 1 | 48 |  |
| 4188 | 5/1/1988 | 4189 | 12/21/2006 | M | 4190 | 3/10/2009 | F | 0 | 27 |  |
| 4212 | 1/1/1957 | 4213 | 12/13/1995 | F | 4214 | 6/1/2003 | F | 0 | 90 | 10/3/2005 |
| 4245 | 1/1/1952 | 4254 | 3/22/1992 | F |  |  |  | 1 | 206 |  |
| 4246 | 1/1/1967 | 4938 | 5/11/1999 | M | 4248 | 4/18/2001 | F | 0 | 23 | 5/21/1999 |
| 4246 | 1/1/1967 | 4247 | 2/1/1996 | F | 4938 | 5/11/1999 | M | 0 | 39 |  |
| 4246 | 1/1/1967 | 4253 | 6/3/2009 | M | 5326 | 1/26/2011 | U | 0 | 20 | 1/25/2011 |
| 4249 | 1/1/1985 | 4250 | 1/17/2000 | M | 4251 | 4/1/2005 | F | 0 | 63 |  |
| 4249 | 1/1/1985 | 4251 | 4/1/2005 | F | 5835 | 10/30/2011 | M | 0 | 79 |  |
| 4271 | 1/1/1953 | 4279 | 12/1/2005 | M |  |  |  | 1 | 45 | 3/7/2010 |
| 4272 | 1/1/1990 | 4940 | 6/1/2009 | M |  |  |  | 1 | 31 |  |
| 4272 | 1/1/1990 | 4939 | 2/1/2003 | F | 4940 | 6/1/2009 | M | 0 | 76 |  |
| 4345 | 1/1/1956 | 4351 | 4/15/1999 | F |  |  |  | 1 | 55 |  |
| 4362 | 1/1/1963 | 4365 | 4/5/1994 | M |  |  |  | 1 | 33 | 8/2/1997 |
